# DNA of neutrophil extracellular traps promote NF-κB-dependent autoimmunity via cGAS/TLR9 in chronic obstructive pulmonary disease

**DOI:** 10.1038/s41392-024-01881-6

**Published:** 2024-06-17

**Authors:** Jun Chen, Tao Wang, Xiaoou Li, Lijuan Gao, Ke Wang, Mengxin Cheng, Zijian Zeng, Lei Chen, Yongchun Shen, Fuqiang Wen

**Affiliations:** Department of Respiratory and Critical Care Medicine, West China Hospital, West China School of Medicine, and Division of Pulmonary Diseases, State Key Laboratory of Biotherapy, Sichuan University, Chengdu, Sichuan 610041 China

**Keywords:** Inflammation, Respiratory tract diseases

## Abstract

Chronic obstructive pulmonary disease (COPD) is characterised by persistent airway inflammation even after cigarette smoking cessation. Neutrophil extracellular traps (NETs) have been implicated in COPD severity and acute airway inflammation induced by short-term cigarette smoke (CS). However, whether and how NETs contribute to sustained airway inflammation in COPD remain unclear. This study aimed to elucidate the immunoregulatory mechanism of NETs in COPD, employing human neutrophils, airway epithelial cells (AECs), dendritic cells (DCs), and a long-term CS-induced COPD mouse model, alongside cyclic guanosine monophosphate-adenosine monophosphate synthase and toll-like receptor 9 knockout mice (*cGAS*^*-−/−*^, *TLR9*^*−/−*^); Additionally, bronchoalveolar lavage fluid (BALF) of COPD patients was examined. Neutrophils from COPD patients released greater cigarette smoke extract (CSE)-induced NETs (CSE-NETs) due to mitochondrial respiratory chain dysfunction. These CSE-NETs, containing oxidatively-damaged DNA (NETs-DNA), promoted AECs proliferation, nuclear factor kappa B (NF-κB) activation, NF-κB-dependent cytokines and type-I interferons production, and DC maturation, which were ameliorated/reversed by silencing/inhibition of cGAS/TLR9. In the COPD mouse model, blocking NETs-DNA-sensing via *cGAS*^−*/−*^ and *TLR9*^−*/−*^ mice, inhibiting NETosis using mitoTEMPO, and degrading NETs-DNA with DNase-I, respectively, reduced NETs infiltrations, airway inflammation, NF-κB activation and NF-κB-dependent cytokines, but not type-I interferons due to IFN-α/β receptor degradation. Elevated NETs components (myeloperoxidase and neutrophil elastase activity) in BALF of COPD smokers correlated with disease severity and NF-κB-dependent cytokine levels, but not type-I interferon levels. In conclusion, NETs-DNA promotes NF-κB-dependent autoimmunity via cGAS/TLR9 in long-term CS exposure-induced COPD. Therefore, targeting NETs-DNA and cGAS/TLR9 emerges as a potential strategy to alleviate persistent airway inflammation in COPD.

## Introduction

Chronic obstructive pulmonary disease (COPD) is a leading cause of global morbidity and mortality,^1^ and is characterised by persistent airflow limitation owing to progressive airway remodelling and alveolar destruction. Cigarette smoke (CS) is the primary risk factor for COPD. CS induces an abnormal inflammatory response, characterised by increased neutrophils, macrophages and T and B lymphocytes recruited from the circulation. These inflammatory cells, together with lung structural cells, including airway epithelial cells (AECs), secrete numerous proinflammatory cytokines to induce chronic airway inflammation, which essentially drives tissue injury in COPD.^2^ Anti-inflammation is considered the mainstream strategy for COPD therapy. Bronchodilators, including long-acting muscarinic antagonists (LAMAs) and long-acting beta-2 agonists (LABAs), either as standalone agents or in combination with anti-inflammation agents (inhaled corticosteroids, ICS), constitute the cornerstone of pharmacological treatment for patients with COPD. Nevertheless, ICS administration alone does not decelerate the rate of lung function decline in patients with COPD.^3^ Moreover, in contrast to the transient acute airway inflammation induced by short-term CS exposure, the airway inflammation observed in COPD persists even after smoking cessation,^4,5^ suggesting that the efficacy of the current therapy is limited by persistent airway inflammation. However, researchers have not completely elucidated the underlying mechanism.

A key aspect of persistent airway inflammation lies in the delayed immunological reactions caused by a complex interplay between innate and adaptive immune systems, particularly in response to pathogen-associated molecular patterns (PAMPs) and damage-associated molecular patterns (DAMPs). DAMPs are notably augmented in COPD due to hazardous substances from CS exposure, oxidative stress-induced apoptosis, and impaired phagocytosis by alveolar macrophages.^6^ Pattern recognition receptors (PRRs), such as Toll-like receptors (TLRs) and cyclic guanosine monophosphate-adenosine monophosphate synthase (cGAS), recognise these DAMPs and initiate signalling cascades, often culminating in the nuclear factor kappa B (NF-κB) activation in COPD.^7,8^ Once activated, NF-κB translocates to the nucleus within AECs and alveolar macrophages,^9,10^ stimulating transcription of inflammatory cytokines genes, including C-X-C motif chemokine ligands (CXCL5), interleukins (such as IL-8, IL-1β), granulocyte-macrophage colony-stimulating factor (GM-CSF), tumour necrosis factor-alpha (TNF-α), and type-I interferons (IFNs, such as IFN-β). These cytokines and IFNs further perpetuate and exacerbate airway inflammation by recruiting and activating neutrophils, macrophages, and lymphocytes (including B cells, T cells and Natural Killer Cells).^2,11^ Additionally, viral infections, such as those caused by adenovirus, can amplify inflammation through their persistent DNA recognised by TLRs and cGAS, to further stimulate cytokines production through NF-κB pathway.^12,13^

On the other hand, there is evidence of autoimmune elements within the COPD pathology, where self-DNA released from apoptosis and necrosis of lung tissue may trigger an inappropriate immune response via TLRs and cGAS activation, thereby contributing to sustained airway inflammation.^14,15^ Recently, research has increasingly highlighted neutrophil extracellular traps (NETs), a web-like structure composed of self-DNA released by the neutrophils, as an active contributor to COPD pathogenesis^16^: (1) Clinically, the induced sputum of patients with both stable and exacerbated COPD shows increased level of NETs,^17,18^ which have been associated with the severity of airflow limitation and microbiota diversity;^19–21^ (2) In vitro, the CS extract (CSE) is capable of inducing NETosis, a releasing process of NETs, on both human^22,23^ and mouse^24^ neutrophils; (3) NETs stimulated by phorbol-12-myristate-13-acetate (PMA)^25,26^ and IL-8^27^ cause cytotoxicity in the human primary AECs and AECs cell lines; (4) PMA-,^25,28^ uric acid (UA)-, and monosodium urate crystal (MSU)-^29^ induced NETs promote, whereas IL-8-induced NETs prohibit,^27^ the expressions of IL-8 and IL-6 on AECs; (5) CSE-induced NETs (CSE-NETs) activate the maturation of myeloid dendritic cells (DCs) to initiate a T-cell-mediated immune response;^22,24^ and (6) In vivo, NETs or self-DNA have been implicated in the development of acute airway inflammation in short-term CS-treated mice.^23,30^ Therefore, NETs participate in short-term CS-induced acute airway inflammation by affecting the function of AECs and DCs. However, researchers have not yet investigated the contribution of DNA components in NETs (NETs-DNA) to persistent airway inflammation in COPD by initiating an autoimmune response.

In this study, we investigated whether and how NETs contribute to long-term CS-induced COPD, with a focus on the immunoregulatory effects of NETs-DNA on AECs, and the role of NETs and targeted pathways as therapeutic targets to alleviate persistent airway inflammation in COPD.

## Results

### CSE induces greater NETosis of neutrophils derived from patients with COPD; CSE-NETs contain chDNA and mtDNA

**Fig. 1:**
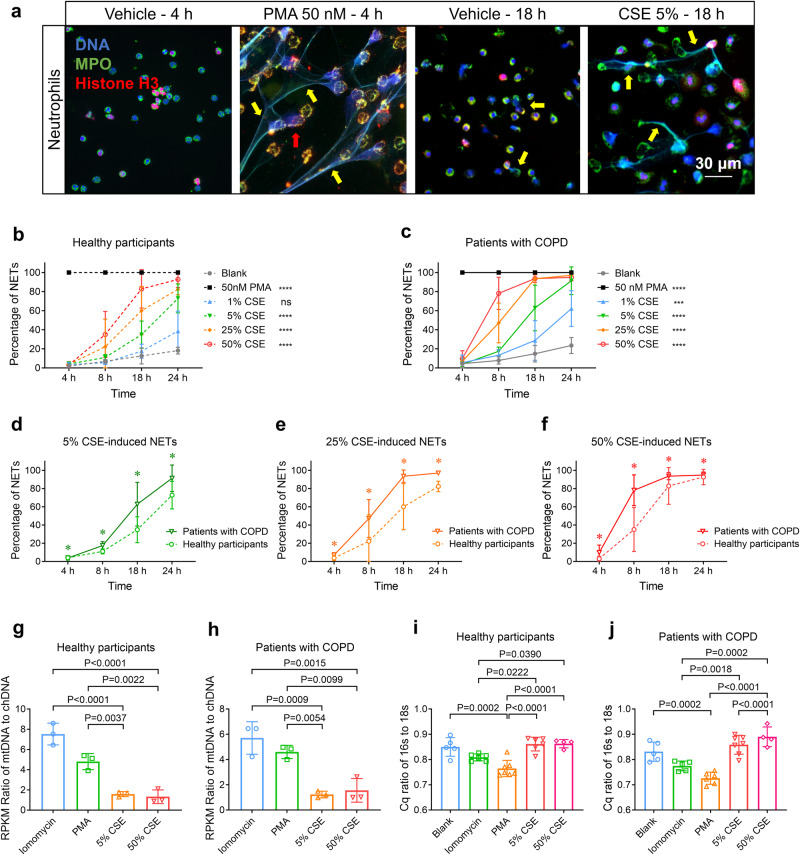
Cigarette smoke extract (CSE) induces human peripheral blood neutrophils (hPBNs) to release neutrophil extracellular traps (NETs) in a dose- and time-dependent manner; CSE-induced NETs contain both mitochondrial DNA (mtDNA) and chromatin DNA (chDNA). Statistical analysis: *n* = 3–11 for each point or each bar in (**b**–**j**) from 3–11 healthy participants or 3–10 patients with COPD, respectively, data were presented as the mean ± standard deviation; Differences are assessed by the (**b**–**f**) two-way or (**g**–**j**) one-way ANOVA analysis of variance, followed Tukey’s honest significant test; *P < **0.05**, ***P < 0.001 and ****P < 0.0001 represent a significant difference from the group of blank or group of healthy participants, the scattered samples and the *p* values are displayed in (**g**–**j**). **a** Representative immunofluorescence co-staining images display the NETs stimulated by 50 nM of phorbol-12-myristate-13-acetate (PMA) for 4 h and 5% CSE for 18 h; The NETs are costained with DNA (blue), MPO (myeloperoxidase, green) and histone H3 (red), and indicated by yellow arrows, scale bar: 30 μm. **b**, **c** The percentage of hPBNs, derived from **b** healthy participants and **c** patients with COPD, to release NETs upon stimulation with increasing dose of CSE and time of incubation, as charcterized by immunofluorescence staining of NETs components (supplementary Method 7); the stimulation of 50 nM of PMA as a positive control. **d**–**f** hPBNs derived from the patients with COPD release a higher percentage of NETs than those of healthy participants under the stimulation of **d**) 5% CSE, **e** 25% CSE, and **f** 50% CSE. **g**, **h** The ratio of the reads per kilobase per million mapped reads (RPKM) of mtDNA to the RPKM of chDNA in NETs induced by ionomycin (4 μM, incubated for 4 h), PMA (50 nM, for 4 h), 5% CSE (for 18 h) and 50% CSE (for 4 h) derived from **g** healthy participants and **h** patients with COPD, as assessed by next-generation sequencing (supplementary Method 10). **i**, **j** The ratio of the quantification cycle (Cq) value of 16 s to Cq of 18 s in spontaneous NETs (Blank) and those induced by ionomycin, PMA, 5% CSE and 50% CSE, derived from **i** healthy participants and **j** patients with COPD, as assessed by quantitative real-time polymerase chain reaction (qPCR) (supplementary Method 13)

CSE induces NETosis in human peripheral blood neutrophils (hPBNs);^22,23^ CS exposure also triggers chDNA and mtDNA release from lung tissue. We asked whether hPBNs from healthy participants and patients with COPD (supplementary Tables S1, S2) exhibit the different capacities of NETosis upon CSE stimulation, and whether the mtDNA:chDNA ratio differs between CSE-induced NETs and those produced by PMA or lomomycin, other known NETosis inducers. We quantified NETosis percentage in hPBNs treated with escalating doses of CSE and varying incubation time, then measured the mtDNA:chDNA ratio within NETs generated by CSE, PMA and iomomycin, respectively, by using next-generation sequencing, and assessing the 16s (mtDNA marker) to 18s (chDNA marker) ratio. We observed that hPBNs derived from patients with COPD (COPD-neutrophils) display a higher potency to release NETs upon stimulation with increasing CSE (Fig. 1a–f, Supplementary Figs. S2d–j, S3a and Supplementary Movie S1). CSE-induced NETs contained both mtDNA and chDNA at a ratio of approximately 1.3:1, whereas those stimulated by lomomycin and PMA contained a higher ratio of mtDNA to chDNA (Fig. 1g, h). These results were further corroborated by the quantification cycle (Cq) value ratio of *16s* to *18s* (Fig. 1i, j). Together, COPD-neutrophils show increased NETosis upon CSE stimulation, and CSE-NETs contain lower ratios of mtDNA:chDNA compared to those induced by PMA and lomomycin.

### CSE-induced NETosis requires mitochondrial ROS, but not NOX

**Fig. 2:**
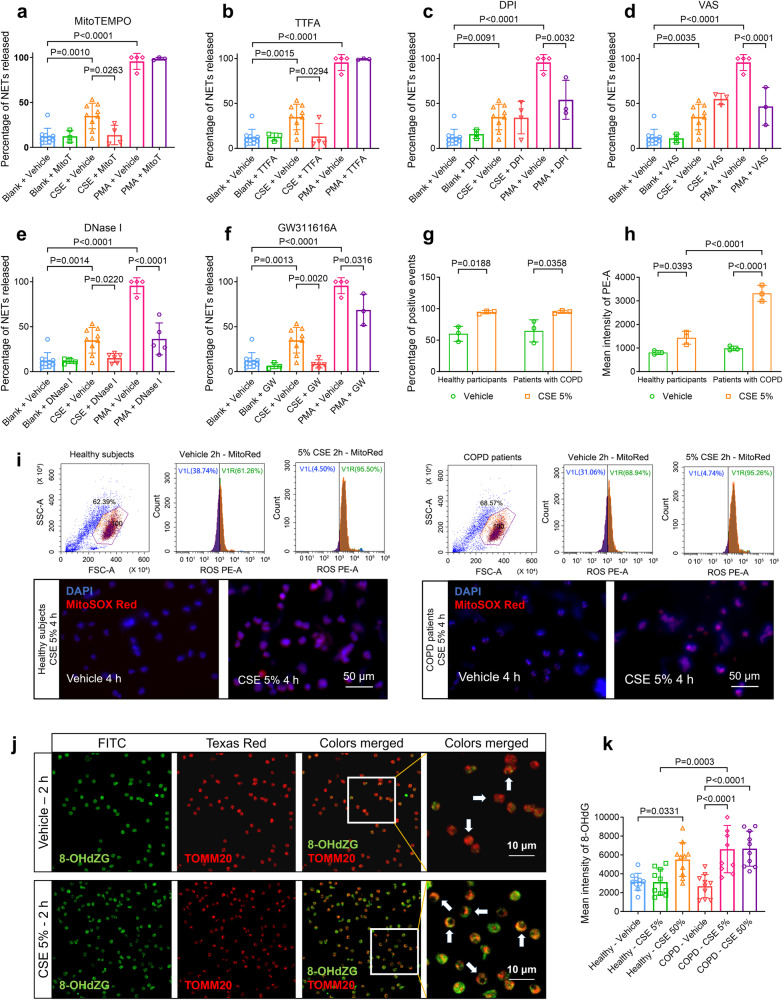
Cigarette smoke extract (CSE)-induced NETosis requires mitochondrial reactive oxygen species (ROS) and mitochondrial respiratory chain, but not nicotinamide adenine dinucleotide phosphate (NADPH) oxidase. Statistical analysis: *n* = 3–10 for each bar in (**a**–**f**, **h**, **i**, **k**), from at least three healthy participants or three patients with COPD, data were presented as the mean ± standard deviation; Differences are assessed by the (**a**–**f**, **k**) one-way or (**h**, **i**) two-way ANOVA analysis of variance, followed Tukey’s honest significant test; *P* < 0.05 represents a significant difference, the scattered samples and the *p* values are displayed in figures. **a**–**f** The effects of several chemicals on the percentage of NETosis of human peripheral blood neutrophils (hPBNs, derived from healthy participants), stimulated by 5% CSE and 50 nM of PMA for 18 h: **a** 50 μM of mitoTEMPO (a mitochondrially targeted antioxidant), **b** 50 μM of thenoyltrifluoroacetone (TTFA, a mitochondrial respiration inhibitor), **c** 50 μM of diphenyleneiodonium chloride (DPI, a NADPH oxidase inhibitor), **d** 50 μM of VAS2870 (VAS, a NADPH oxidase inhibitor), **e** 200 IU/mL of deoxyribonuclease-I (DNase-I, an endonuclease for single- and double-stranded DNA) and **f** 50 μM of GW311616A (a selective human neutrophil elastase inhibitor), as characterized by immunofluorescence staining of NETs components (supplementary Method 7). **g**–**i** The incubation of 5% CSE for 2 or 4 h induces the release of mitochondrial ROS (stained by mitoSOX Red, supplementary Method 8, 17) by hPBNs from both healthy participants and patients with COPD, as assessed by **i** flow cytometry and fluorescence staining (scale bar: 50 μm), and summarised in **g** the percentage of positive events and **h** the mean intensity of mitoSOX Red. **j** The immunofluorescence colocalization of 8-hydroxy-2’-deoxyguanosine (8-OHdG, a marker of oxidative stress to DNA, green) and translocase of the outer mitochondrial membrane complex subunit 20 (TOMM20, a mitochondrial outer membrane marker, red), with and without the treatment of 5% CSE (supplementary Method 8). Note: oxidative-damaged DNA are presented on the mitochondrial membrane following the treatment of 5% CSE for 2 h (scale bar: 10 μm). **k** The fluorescence intensity (mean) of 8-OHdZG on hPBNs treated with 5% CSE and 50% CSE from healthy participants and patients with COPD

NETosis triggered by PMA is nicotinamide adenine dinucleotide phosphate (NADPH) oxidase (NOX)-dependent, but the mechanism of NETosis induced by CSE remains elusive. Given that CSE-NETs exhibit heightened mtDNA content compared to PMA-NETs, we asked whether mitochondrial reactive oxygen species (mtROS) or NOX played a pivotal role in CSE-induced NETosis. We thus evaluated the inhibitory effects on NETosis induced by 5% CSE or PMA using respective inhibitors for mtROS (mitoTEMPO), mitochondrial respiration (thenoyltrifluoroacetone, TTFA), and NOX (diphenyleneiodonium chloride [DPI] and VAS2870 [VAS]). Inhibiting mtROS and mitochondrial respiration using mitoTEMPO and TTFA selectively suppressed 5% CSE-induced, but not PMA-induced, NETosis (Fig. 2a, b) in hPBNs derived from healthy participants, whereas NOX inhibitors DPI and VAS specifically attenuated PMA-induced, but not 5% CSE-induced NETosis (Fig. 2c, d); Deoxyribonuclease-I (DNase-I, endonuclease for single/double-stranded DNA) and GW311616A (GW, inhibitor of neutrophil elastase [NE]) treatments were effective against both 5% CSE- and PMA-induced NETosis (Fig. 2e, f and Supplementary Fig. S3b), with comparable results observed for COPD patient-derived hPBNs (Supplementary Fig. S4a–f). Treatment with 5% CSE resulted in elevated mtROS levels, particularly in hPBNs from COPD patients (Fig. 2g–i), with co-staining suggesting that mitochondria are the primary source of cellular ROS (supplementary Fig. S4g). Additionally, 5% CSE exposure triggered co-localisation of oxidative DNA damage marker 8-OHdZG (8-hydroxy-2’-deoxyguanosine) with mitochondrial membrane protein TOMM20 (outer mitochondrial membrane complex subunit 20) on the hPBNs (Fig. 2j) and enhanced 8-OHdZG staining in CSE-NETs derived from both healthy participants and patients with COPD (Fig. 2k and supplementary Fig. S4h). Together, CSE-induced NETosis relies on mtROS and mitochondrial respiration; CSE also provokes DNA damage within the mitochondrial membrane of hPBNs and in the formed CSE-NETs.

### CSE-NETs increase the proliferation and production of both NF-κB-dependent cytokines and type-I IFNs in human AECs (hAECs), and the maturation of human DCs (hDCs)

**Fig. 3:**
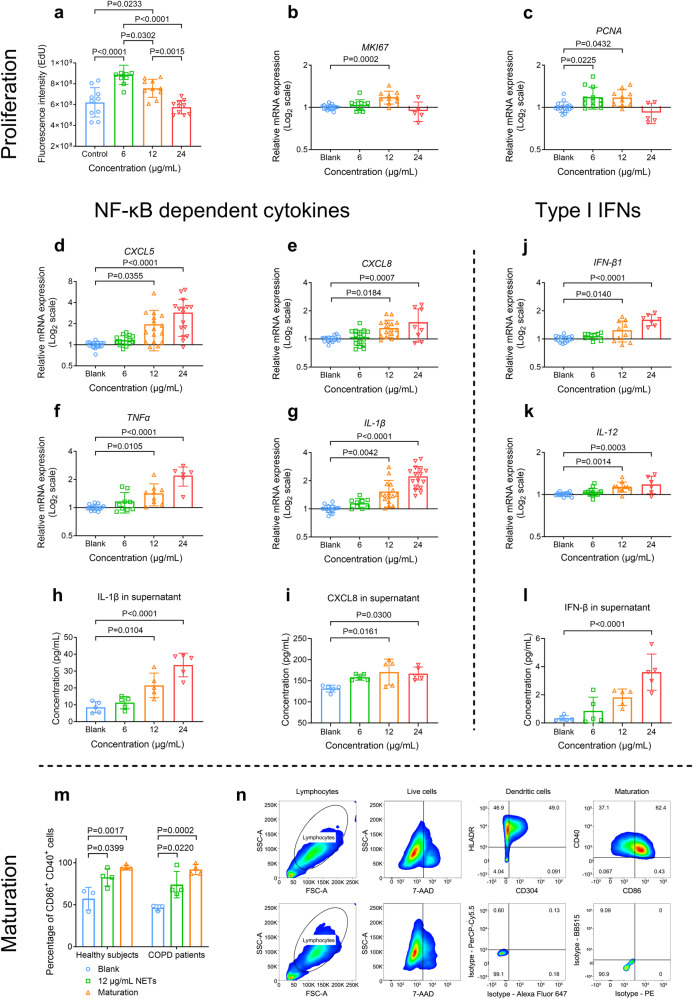
Neutrophil extracellular traps (NETs) induced by cigarette smoke extract (CSE-NETs) dose-dependently promote the proliferation and the gene expressions of both nuclear factor kappa B (NF-κB)-dependent inflammatory cytokines and type-I interferons (IFNs) in the human airway epithelial cells (hAECs); CSE-NETs promote the maturation of human dendritic cells (DCs). Statistical analysis: *n* = 3–16 for each bar in (**a**–**m**) from at least three independent experiments, data were presented as the mean ± standard deviation; Differences are assessed by the (**a**–**l**) one-way or (m) two-way ANOVA analysis of variance, followed Tukey’s honest significant test; *P* < 0.05 represents a significant difference, the scattered samples and the *p* values are displayed in figures. **a**–**c** Human peripheral neutrophils were stimulated by 5% CSE for 18 h to induce NETs, which were subsequently collected and quantified for DNA concentrations. Effects of 6, 12, and 24 μg/mL NETs (incubated for 48 h) on the proliferation of hAECs, as assessed by the **a** EdU proliferation assay (supplementary Method 11) and mRNA expressions of **b** *MKI67* and **c** *PCNA* (both are markers of proliferation, supplementary Method 13). **d**–**l** Effects of 6, 12, and 24 μg/mL NETs on the mRNA expressions and soluble levels of NF-κB-dependent inflammatory cytokines and type-I IFNs of hAECs (supplementary Method 13, 26): **d** mRNA expression of *CXCL5* (C-X-C motif chemokine ligand 5), **e** mRNA expression of *CXCL8*, **f** mRNA expression of *TNFα* (tumour necrosis factor-alpha), **g** mRNA expression of *IL-1β* (interleukin 1β), **h** soluble levels of IL-1β in cell-culture supernatants, **i** soluble levels of CXCL8 in cell-culture supernatants, **j** mRNA expression of *IFN-β1*, **k** mRNA expression of *IL-12* and **l** soluble levels of IFN-β in cell-culture supernatants. **m**, **n** 12 μg/mL of NETs promote the maturation of dendritic cells (differentiated from peripheral blood monocytes) from both healthy participants and patients with COPD (supplementary Method 15); the maturation is evaluated by **m** the percentage of CD86^+^ CD40^+^ cells, as assessed by flow cytometry with a gating strategy illustrated in (**n**) (Supplementary Method 17)

Previous studies have shown that PMA, IL-8, UA, and MSU-induced NETs can exert cytotoxic effects or modulate the expression of IL-8 and IL-6 on AECs;^25–29^ however, the impact of CSE-induced NETs on primary human AECs remains uncertain. To address this, we prepared 5% CSE-induced NETs from healthy participants (Supplementary Method 9 and Supplementary Fig. S5a), and treated hAECs with escalating doses of NETs-DNA. We found that both 6 μg/mL and 12 μg/mL, but not 24 μg/mL, CSE-NETs promoted hAECs proliferation (Fig. 3a–c). In hAEC, the NETs treatment led to a dose-dependent increase in gene expression and soluble protein levels of NF-κB-dependent cytokines (CXCL5, IL-8, TNFα and IL-1β), as well as type-I IFNs (IFN-β1 and IL-12, Fig. 3d–l). Additionally, NETs treatment facilitated NF-κB (P65 and P50) activation in hAECs (Supplementary Figs. S5b, S6a–e), and maturation of hDCs (Fig. 3m, n). Together, CSE-NETs promote the proliferation and production of NF-κB-dependent cytokines and type-I IFNs in hAECs, and enhance the maturation of hDCs.

### cGAS/TLR9 is required for CSE-NETs-induced proliferation and production of both NF-κB-dependent cytokines and type-I IFNs in hAECs, and maturation of hDCs

**Fig. 4:**
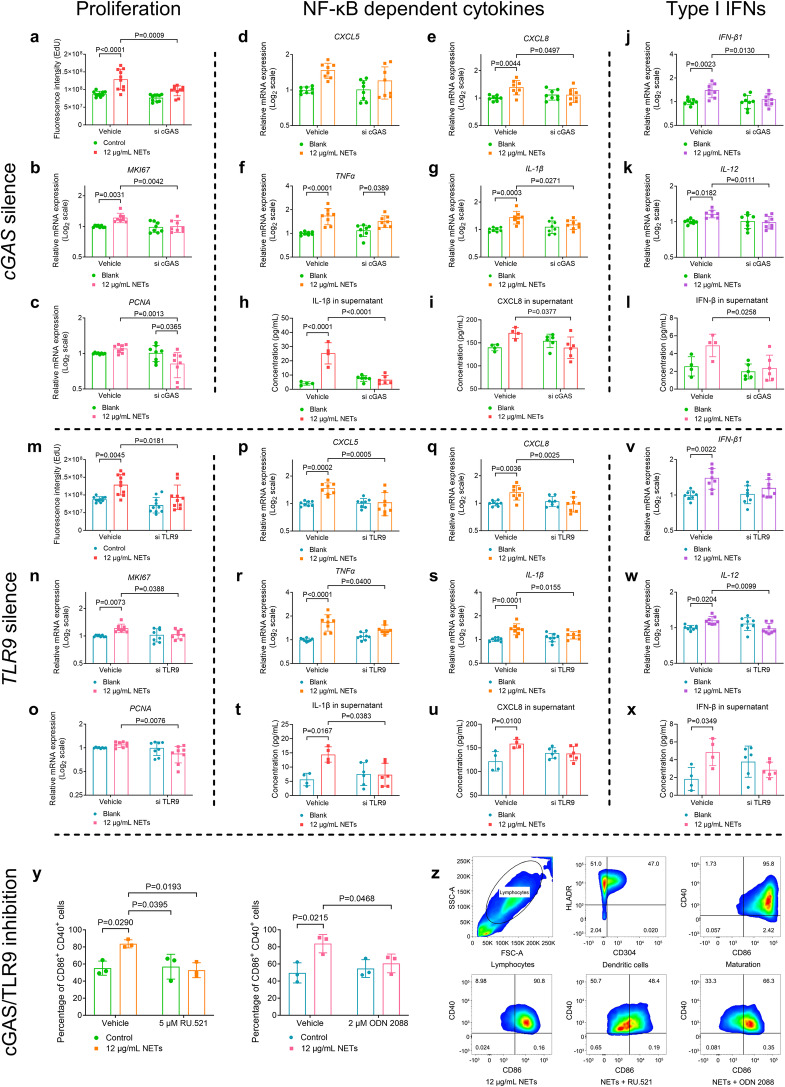
Cyclic guanosine monophosphate-adenosine monophosphate synthase (cGAS), and toll-like receptor 9 (TLR9) are required for the neutrophil extracellular traps (NETs)-stimulated proliferation, expressions of both nuclear factor kappa B (NF-κB)-dependent inflammatory cytokines and type-I interferons (IFNs) in human airway epithelial cells (hAECs), and maturation of human dendritic cells (hDCs). Statistical analysis: *n* = 6–10 for each bar in (**a**–**x**), *n* = *3* for each bar in **y** from at least three independent experiments, data were presented as the mean ± standard deviation; Differences are assessed by the **a**–**y** two-way ANOVA analysis of variance, followed Tukey’s honest significant test; *P* < 0.05 represents a significant difference, the scattered samples and the *p* values are displayed in figures. Effects of **a**–**c** *cGAS* **a**nd **m**–**o** *TLR9* silencing on 12 μg/mL of NETs-stimulated proliferation of hAECs, as assessed by the **a**, **m** EdU proliferation assay (supplementary Method 11), and the mRNA expression of **b**, **n** *MKI67* and **c**, **o** *PCNA* (both are markers of proliferation, supplementary Method 13). Effects of **d**–**l** *cGAS* and **p**, **x** *TLR9* silencing on 12 μg/mL of NETs-induced mRNA expression and soluble levels of NF-κB-dependent inflammatory cytokines and type-I interferons (IFNs) in hAECs (supplementary Method 13, 26): **d**, **p** mRNA expression of *CXCL5* (C-X-C motif chemokine ligand 5), **e**, **q** mRNA expression of *CXCL8*, **f**, **r** mRNA expression of *TNFα* (tumour necrosis factor-alpha), **g**, **s** mRNA expression of *IL-1β* (interleukin 1β), **h**, **t** soluble levels of IL-1β in cell-culture supernatants, **i**, **u** soluble levels of CXCL8 in cell-culture supernatants, **j**, **v** mRNA expression of *IFN-β1*, **k**, **w** mRNA expression of *IL-12* and **l**, **x** soluble levels of IFN-β in cell-culture supernatants. **y** The inhibition of cGAS and TLR9 by 5 μM of RU.521 and 2 μM of ODN 2088, respectively, reduces 12 μg/mL of NETs-mediated maturation of hDCs (supplementary Method 16). **z** Representative flow cytometry images display the gating strategy and maturation of DCs treated with either RU.521 or ODN 2088, as evaluated by the percentage of CD86^+^ CD40^+^ cells (supplementary Method 17)

cGAS/TLR9 senses mtDNA/chDNA to activate downstream signalling cascades. Given the presence of mtDNA and chDNA in CSE-NETs, we asked whether these receptors contribute to the proliferation and production of cytokines in hAECs treated with CSE-NETs. We found that silencing both *cGAS* and *TLR9* reduced NETs-mediated hAECs proliferation (Supplementary Fig. S5c–f and Fig. 4a–c, m–o). *cGAS* silencing ameliorated the NETs-induced gene expression and/or soluble protein levels of CXCL8, IL-1β, IFN-β1, and IL-12 (Fig. 4d–l), whereas *TLR9* silencing ameliorated NETs-induced gene expression and/or soluble protein levels of CXCL5, TNFα, IL-1β and IL-12 (Fig. 4p–x). Furthermore, NETs-induced NF-κB (P65 and P50) activation in hAECs was inhibited upon *cGAS* and *TLR9* silencing (Supplementary Fig. S6a–e). The inhibitors RU.521 and ODN 2088, targeting cGAS and TLR9, respectively, also decreased NETs-mediated DC maturation (Fig. 4y, z). Together, DNA sensor cGAS/TLR9 is essential for CSE-NETs-induced proliferation, production of NF-κB-dependent cytokines and type-I IFNs in hAECs, and hDCs maturation.

### Knockout of cGAS and TLR9, respectively alleviates airway inflammation, NETs infiltration and production of NF-κB-dependent cytokines, but not type-I IFNs, in a COPD mouse model

**Fig. 5:**
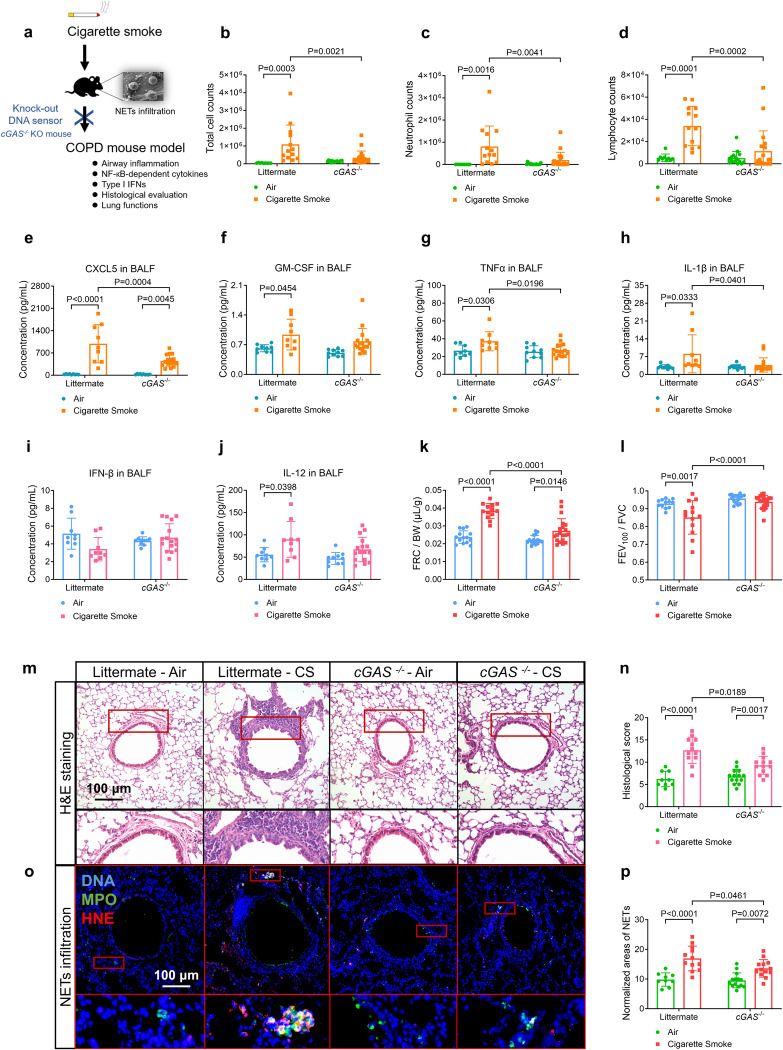
Guanosine monophosphate-adenosine monophosphate synthase (cGAS) knock-out (*cGAS*^*−/−*^) mice treated with cigarette smoke (CS) exposure display decreased productions of nuclear factor kappa B (NF-κB)-dependent inflammatory cytokines, but not type-I interferons (IFNs), alleviated airway inflammation, infiltration of neutrophil extracellular traps (NETs) and improved lung functions, as compared with CS-treated littermate. Statistical analysis: *n* = 9–20 for each bar in (**b**–**l**, **n**, **p**), data were presented as the mean ± standard deviation; Differences are assessed by the **b**–**l**, **n**, **p** two-way ANOVA analysis of variance, followed by Tukey’s honest significant test; *P* < 0.05 represents a significant difference, the scattered samples and the *p* values are displayed in figures. **a** A brief outline for the experiments of the COPD mouse model (supplementary Method 18, 19). **b**–**d** CS-treate**d** *cGAS*^*−/−*^ mice display alleviated airway inflammation as reflected by: **b** total cell counts, **c** neutrophil counts and **d** lymphocyte counts in the bronchoalveolar lavage fluid (BALF, supplementary Method 22). **e**–**h** CS-tr**e**ated *cGAS*^−*/−*^ mice reveal overall reduced productions of NF-κB-dependent inflammatory cytokines in the BALF (supplementary Method 22, 26): **e** C-X-C motif chemokine ligand 5 (CXCL5), **f** granulocyte-macrophage colony-stimulating factor (GM-CSF), **g** tumour necrosis factor-alpha (TNFα) and **h** interleukin 1β (IL-1β). **i**, **j** No s**i**gnificant changes of type-I IFNs are shown in BALF of either CS-treated littermate or *cGAS*^*−*^^*/−*^ mice: **i** IFN-β, **j** IL-12. **k**, **l** CS-treated *cGAS*^−*/−*^ mice reveal alleviated emphysema and airflow limitation in lung function tests (supplementary Method 21) as evaluated by: **k** functional residual capacity/body weight (FRC/BW), **l** forced expiratory volume at 100 ms/forced vital capacity (FEV_100_/FVC). **m** Representative images of hematoxylin-eosin (H&E)-stained lung slices display the decreased severity of airway inflammation in the CS-treated *cGAS*^*−/−*^ mice, compared with CS-treated littermate (scale bar: 100 μm, Supplementary Method 23), as summarised in **n** histological score. **o** Representative immunofluorescence images display the decreased infiltration of NETs (co-stained with DNA, myeloperoxidase, and histone H3) in the lung slices of CS-treated *cGAS*^*−/−*^ mice, compared with CS-treated littermate (scale bar: 100 μm, supplementary Method 24), as summarised in (**p**) normalised area of NETs

We then investigated whether the knockout of NETs-DNA sensor cGAS/TLR9 could mitigate long-term CS exposure-induced airway inflammation in an established COPD mouse model (Supplementary Method 19). We exposed *cGAS* and *TLR9* knockout mice (*cGAS*^*−/−*^ and *TLR9*^*−/−*^) and corresponding littermate to CS, and evaluated their severity of airway inflammation and changes in lung functions (Fig. 5a and Supplementary Fig. S7). Compared to CS-littermate (CS-littermate) mice, CS-treated *cGAS*^*−/−*^ mice (CS-*cGAS*^*−/−*^) displayed reduced total cells, neutrophils and lymphocytes in bronchoalveolar lavage fluid (BALF, Fig. 5b–d), decreased NF-κB-dependent cytokines, including CXCL5, TNFα, IL-1β in BALF (Fig. 5e–h), CXCL5, GM-CSF, IL-1β in serum (Supplementary Fig. S9n–p), and CXCL5, IL-1β in lung tissue slices (Supplementary Fig. S9c, d, h–k), suppressed lung tissue NF-κB P65 activation (Supplementary Fig. S9q), improved lung function indicated by reduced FRC/BW (functional residual capacity/body weight) and increased FEV_100_/FVC (forced expiratory volume at 100 ms/forced vital capacity, Fig. 5k, l), and reduced histological score, mucin stain score, and mean linear intercept (MLI) of the alveoli (Fig. 5m, n and Supplementary Fig. S9a, b, f, g). The severity of NETs infiltration, as assessed by immunofluorescence co-staining of main components of NETs (Supplementary Method 24 and Supplementary Fig. S8), decreased in the CS-*cGAS*^*−/−*^ mice (Fig. 5o, p), and was correlated with neutrophil count, histological score, and FEV_100_/FVC in CS-treated mice (Supplementary Fig. S9t–v). However, no change in type-I IFNs was observed in either CS-littermates or CS-*cGAS*^*−/−*^ mice (Fig. 5i, j and Supplementary Fig. S9e, l, m, r, s), potentially due to impaired expression of IFN-α/β receptor subunit-1 (IFNAR1) in hAECs upon CS exposure (Supplementary Fig. S6f). A similar amelioration pattern was seen in CS-*TLR9*^*−/−*^ mice (Supplementary Figs. S10, S11). Collectively, cGAS and TLR9 Knockout independently mitigated long-term CS-induced NF-κB (but not typer-I IFNs)-related airway inflammation.

### Inhibition of NETosis by mitoTEMPO alleviates airway inflammation, NETs infiltration and production of NF-κB-dependent cytokines, but not type-I IFN, in the COPD mouse model

**Fig. 6:**
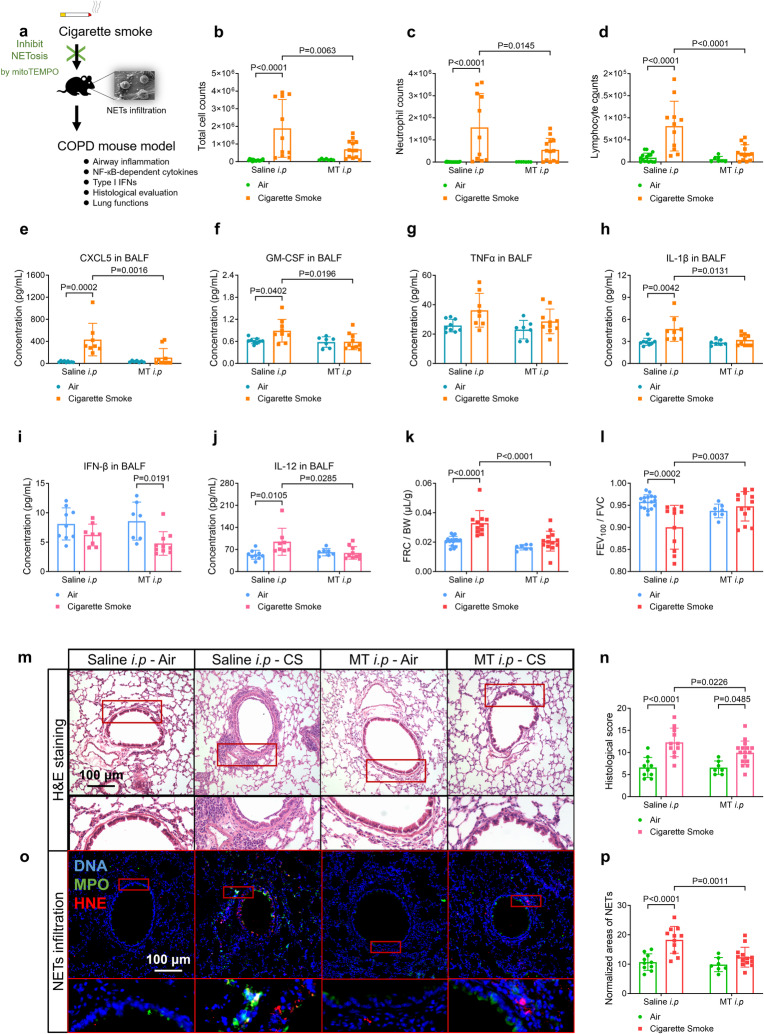
Cigarette smoke (CS)-exposed wild-type mice treated with an intraperitoneal injection (i.p) of mitoTEMPO (MT) reveal decreased productions of nuclear factor kappa B (NF-κB)-dependent inflammatory cytokines, but not type-I interferons (IFNs), alleviated airway inflammation, infiltration of neutrophil extracellular traps (NETs), and improved lung functions, as compared with CS-treated saline i.p mice. Statistical analysis: *n* = 7–16 for each bar in (**b**–**l**, **n**, **p**), data were presented as the mean ± standard deviation; Differences are assessed by the (**b**–**l**, **n**, **p**) two-way ANOVA analysis of variance, followed Tukey’s honest significant test; *P* < 0.05 represents a significant difference, the scattered samples and the *p* values are displayed in figures. **a** A brief outline for the experiments of the COPD mouse model (Supplementary Method 18, 19). **b**–**d** CS-treate**d** MT i.p mice reveal alleviated airway inflammation as reflected by the **b** total cell counts, **c** neutrophil counts and **d** lymphocyte counts in the bronchoalveolar lavage fluid (BALF, Supplementary Method 22). **e**–**h** CS-tr**e**ated MT i.p mice reveal overall reduced production of NF-κB-dependent inflammatory cytokines in the BALF (supplementary Method 22, 26): **e** C-X-C motif chemokine ligand 5 (CXCL5), **f** granulocyte-macrophage colony-stimulating factor (GM-CSF), **g** tumour necrosis factor-alpha (TNFα) and **h** interleukin 1β (IL-1β). **i**, **j** CS-treated MT i.p mice reveal reduced levels of **j** IL-12, but not **i** IFN-β, in the BALF, compared with CS-treated saline i.p mice. **k**, **l** CS-treated MT i.p mice reveal alleviated emphysema and airflow limitation in lung function tests (supplementary Method 21) as evaluated by **k** functional residual capacity/body weight (FRC/BW) and **l** forced expiratory volume at 100 ms/forced vital capacity (FEV_100_/FVC). **m** Representative images of hematoxylin-eosin (H&E)-stained lung slices display the decreased severity of airway inflammation in CS-treated MT i.p mice, compared with CS-treated saline i.p mice (scale bar: 100 μm, supplementary Method 23), as summarised in **n** histological score. **o** Representative immunofluorescence images reveal the decreased infiltration of NETs (co-stained with DNA, myeloperoxidase and histone H3) in lung slices of CS-treated MT i.p mice, compared with CS-treated saline i.p mice (scale bar: 100 μm, supplementary Method 24), as summarised in (**p**) normalised area of NETs

To further investigate the contribution of NETs to long-term CS exposure-induced airway inflammation, we treated the COPD mouse model with mitoTEMPO, the specific mtROS inhibitor that inhibits CSE-induced NETosis as shown above. Compared to CS-treated controls, CS-treated mice receiving intraperitoneal injection of mitoTEMPO (CS-MT i.p mice, Fig. 6a) displayed reduced total cells, neutrophils, and lymphocytes in BALF (Fig. 6b–d), decreased NF-κB-dependent cytokines (CXCL5, GM-CSF and IL-1β) in both BALF (Fig. 6e–h), serum (Supplementary Fig. S12n–p) and lung tissue slices (Supplementary Fig. S12c, d, h–k), suppressed lung tissue NF-κB P65 activation (Supplementary Fig. S12q), improved lung function indicated by reduced FRC/BW and increased FEV_100_/FVC (Fig. 6k, l), and reduced histological score, mucin stain score, and MLI of alveoli (Fig. 6m, n and Supplementary Fig. S12a, b, f, g). The severity of NETs infiltration decreased in the CS-MT i.p mice (Fig. 6o, p), and was correlated with IL-1β and CXCL5 levels, but not IFN-β1 level, in the BALF of CS-treated mice (Supplementary Fig. S12t–v). No changes in type-I IFNs were observed (Fig. 6i, j and Supplementary Fig. S12e, l, m, r, s). Overall, mitoTEMPO alleviated long-term CS-induced NETs infiltration and NF-κB (but not typer-I IFNs)-related airway inflammation.

### Degradation of NETs-DNA by DNase-I alleviates NETs infiltration and emphysema-phenotype in the COPD mouse model

**Fig. 7:**
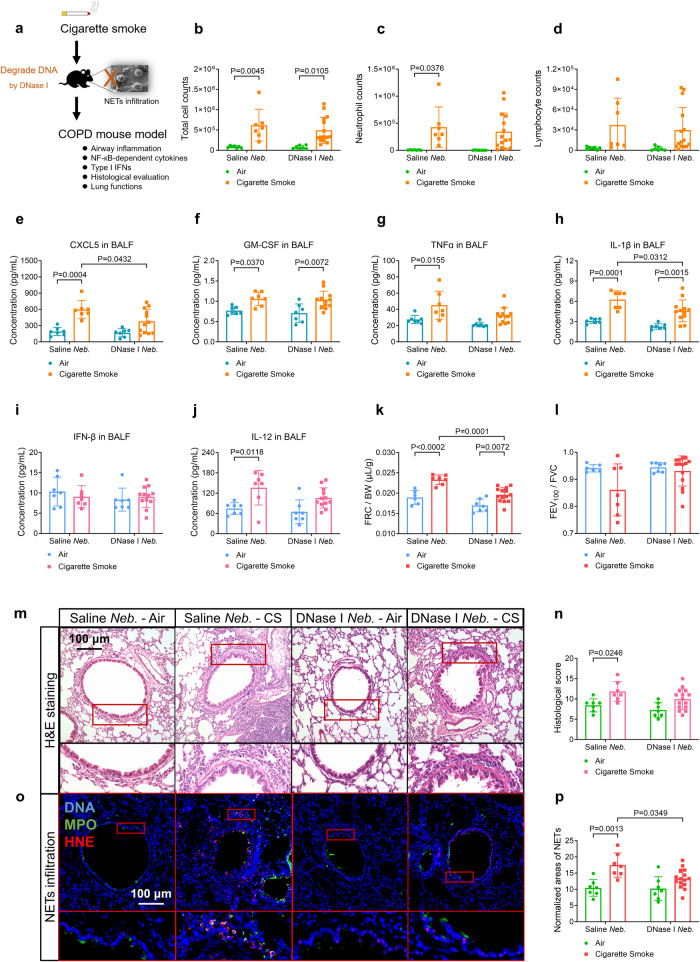
Cigarette smoke (CS)-exposed wild-type mice treated with the nebulization (*Neb*.) of deoxyribonuclease-I (DNase-I) reveal the decreased infiltration of neutrophil extracellular traps (NETs) and improved lung function, as compared with CS-treated saline *Neb*. mice. Statistical analysis: *n* = 7–15 for each bar in (**b**–**l**, **n**, **p**), data were presented as the mean ± standard deviation; Differences are assessed by the (**b**–**l**, **n**, **p**) two-way ANOVA analysis of variance, followed Tukey’s honest significant test; *P* < 0.05 represents a significant difference, the scattered samples and the *p* values are displayed in figures. **a** A brief outline for the experiments of the COPD mouse model (Supplementary Method 18, 19). **b**–**d** No significant changes of cell counts in bronchoalveolar lavage fluid (BALF) of CS-treated DNase-I *Neb*. mice are observed (Supplementary Method 22): **b** total cell counts, **c** neutrophil counts and **d** lymphocyte counts. **e**–**h** CS-tr**e**ated DNase-I *Neb*. mice reveal the decreased levels of **e** C-X-C motif chemokine ligand 5 (CXCL5) and **h** interleukin 1β (IL-1β), but not that of **f** granulocyte-macrophage colony-stimulating factor (GM-CSF) or **g** TNFα (tumour necrosis factor-alpha) in the BALF (supplementary Method 22, 26). **i**, **j** No s**i**gnificant changes of type-I interferons (IFNs) level in the BALF of either CS-treated saline *Neb*. or DNase-I *Neb*. mice are observed: **i** IFN-β, **j** IL-12. **k**, **l** CS-treated DNase-I *Neb*. mice reveal alleviated emphysema, but not airflow limitation, in lung function tests (Supplementary Method 21) as evaluated by: **k** functional residual capacity/body weight (FRC/BW), **l** forced expiratory volume at 100 ms/forced vital capacity (FEV_100_/FVC). **m** Representative images of hematoxylin-eosin (H&E)-stained lung slices reveal that significant changes in the severity of airway inflammation are absent in the CS-treated DNase-I *Neb*. mice (scale bar: 100 μm, Supplementary Method 23), as summarised in **n** histological score. **o** Representative immunofluorescence images display the decreased infiltration of NETs (co-stained with DNA, MPO and Histone H3) in lung slices of the CS-treated DNase-I *Neb*. mice, compared with CS-treated saline *Neb*. mice (scale bar: 100 μm, Supplementary Method 24), as summarised in (**p**) normalised area of NETs

DNase-I has been shown to reduce airway inflammation induced by acute CS exposure.^23^ To explore the therapeutic potential of targeting NETs-DNA to mitigate airway inflammation induced by long-term CS exposure, we employed DNase-I to degrade NETs-DNA in the COPD mouse model. Compared with CS-treated control, CS-treated mice receiving nebulised DNase-I (DNase-I *Neb*. mice, Fig. 7a) showed alleviated emphysema-phenotype, as indicated by reduced FRC/BW (Fig. 7k) and MLI of alveoli (Fig. S13a, f), although there were only downward trends for total and different cell counts in BALF (Fig. 7b–d), histological scores (Fig. 7m, n), and mucin staining scores (Supplementary Fig. S13b, g) were observed in DNase-I *Neb*. mice. Nevertheless, NF-κB-dependent cytokines CXCL5 and IL-1β levels in BALF (Fig. 7e–h) and lung tissue slices (Supplementary Fig. S13c, d, h–k), IL-1β level in serum (Supplementary Fig. S13n–p), and NF-κB P65 activation in lung tissues (Supplementary Fig. S13q) significantly decreased in DNase-I *Neb*. mice. The severity of NETs infiltration also decreased in CS-DNase-I *Neb*. mice (Fig. 7o, p), and was correlated with MLI of alveoli and FRC/BW in CS-treated mice (Supplementary Fig. S13t, u). No changes in type-I IFNs were observed (Fig. 7i, j and Supplementary Fig. S13e, l, m, r, s). Together, DNase-I treatment alleviates NETs infiltration and emphysema features in the COPD mouse model. Supplementary Table S3 summarises the improved indicators seen in CS-*cGAS*^*−/−*^, *TLR9*^−*/−*^, MT i.p, and DNase-I *Neb*. mice.

### Disordered NF-κB-dependent cytokines, but not type-I IFNs, are correlated with MPO and NE activity in the BALF of patients with COPD

**Fig. 8:**
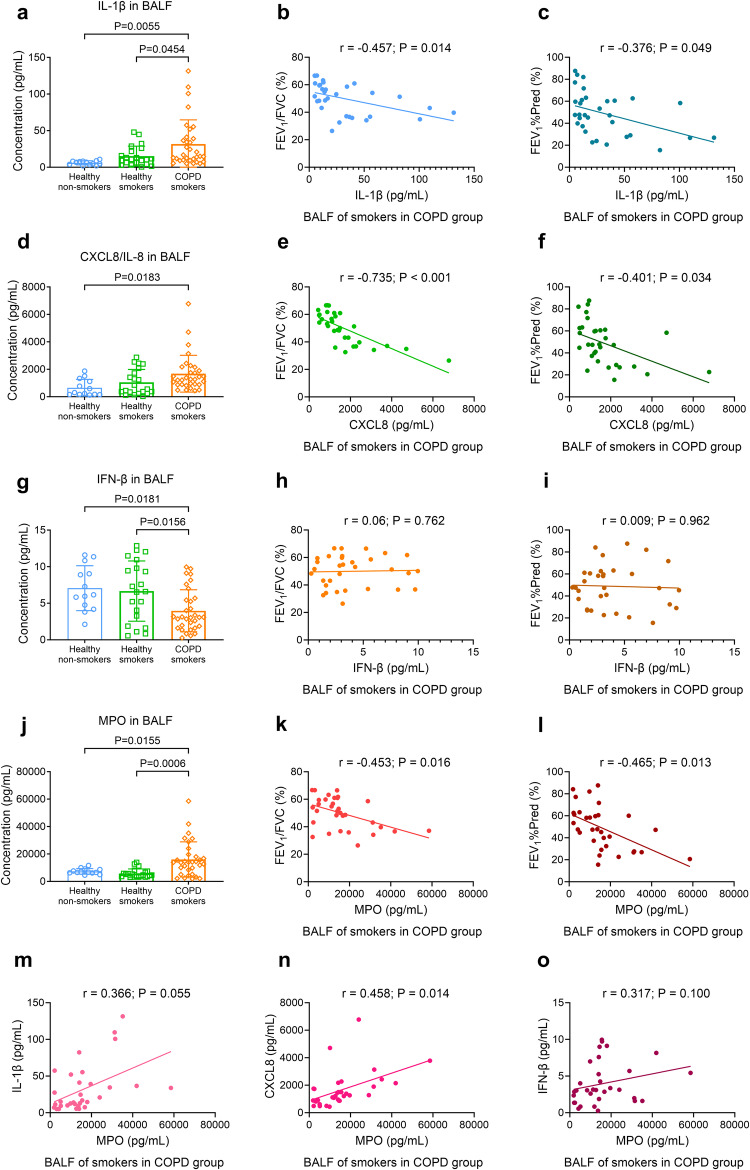
The level of myeloperoxidase (MPO) is correlated with the level of interleukin 1β (IL-1β) and level C-X-C motif chemokine ligand 8 (CXCL8), but not correlated with the level of interferons-β (IFN-β) in bronchoalveolar lavage fluid (BALF) of smokers in the group of patients with COPD, after controlling for their age, sex, body mass index (BMI) and smoking history. Statistical analysis: *n* = 13 non-smokers and 21 smokers in group of healthy participants, *n* = 32 smokers in group of patients with COPD in (**a**, **d**, **g**, **j**), *n* = 32 in (**b**, **c**, **e**, **f**, **h**, **i**, **k**–**o**), data were presented as the mean ± standard deviation; Differences in (**a**, **d**, **g**, **j**) are assessed by one-way analysis of variance, followed Tukey’s honest significant test; In (**b**, **c**, **e**, **f**, **h**, **i**, **k**, **l**, **m**–**o**), Pearson’s partial correlation test are performed by controlling for age, sex, BMI and smoking history of smokers in the group of patients with COPD, followed by the multiple linear regression analysis; *P* < **0.05** represents a significant difference, the scattered samples and the *p* values are displayed in figures. **a** IL-1β level is significantly increased in the BALF of smokers in the group of patients with COPD, compared with that of non-smokers and smokers in the healthy group, and negatively correlated with **b** ratio of FEV_1_ to forced vital capacity (FEV_1_/FVC), and **c** ratio of forced expiratory volume at 1 s (FEV_1_) to predicted FEV_1_ (FEV_1_%Pred, supplementary Method 1, 2, 3, 26), respectively. **d** CXCL8 level is significantly increased in the BALF of smokers in the group of patients with COPD, compared with that of non-smokers in the healthy group, and negatively correlated with **e** FEV_1_/FVC and **f** FEV_1_%Pred, respectively. **g** IFN-β level is significantly decreased in the BALF of smokers in the group of patients with COPD, compared with that of non-smokers and smokers in the healthy group; There is no correlation between IFN-β level and **h** FEV_1_/FVC, or IFN-β level and **i** FEV_1_%Pred**. j** MPO level is significantly increased in the BALF of smokers in the group of patients with COPD, compared with that of non-smokers and smokers in the healthy group, and negatively correlated with **k** FEV_1_/FVC and **l** FEV_1_%Pred, respectively. In the BALF of COPD smokers, MPO level is correlated with the level of **n** CXCL8, but not the level of **m** IL-1β or **o** IFN-β

Patients with COPD, both stable and exacerbated, exhibit increased NETs in induced sputum^17,18^ that correlate with airflow limitation severity and microbiota diversity.^19–21^ However, the relationship between NETs components, NF-κB-dependent cytokines, and type-I IFNs in BALF of COPD patients remains unexplored. We collected BALF from 34 healthy participants (13 without and 21 with smoking history) and 32 COPD patients with smoking history (Supplementary Methods 1–3 and Supplementary Tables S1, S2). In comparison to healthy non-smokers and smokers, patients with COPD showed increased levels of NF-κB-dependent cytokines (IL-1β, CXCL8 in BALF, Fig. 8a, d, and CXCL5, IL-1β in lung tissue slices, Supplementary Fig. S16e–j) and NETs components (MPO, NE activity in BALF, Fig. 8j, Supplementary Fig. S15a), but decreased the level of type-I IFN (IFN-β in BLAF, Fig. 8g, and in lung tissue slices, Supplementary Fig. S16k–m). The levels of IL-1β, CXCL8, and MPO correlated negatively with FEV_1_/FVC and FEV_1_%Pred (the ratio of tested FEV_1_ to predicted FEV_1_), respectively, after adjusting for age, sex, body mass index (BMI), and smoking history (Fig. 8b, c, e, f, k, l). NE activity showed a negative correlation with FEV_1_/FVC (supplementary Fig. S15b, c). Conversely, IFN-β level did not correlate with either FEV_1_/FVC or FEV_1_%Pred (Fig. 8h, i). Notably, MPO level positively correlated with CXCL8 level, and NE activity correlated with IL-1β and CXCL8 levels, respectively (Fig. 8m, n and Supplementary Fig. S15d, e); however, neither MPO nor NE activity correlated with IFN-β level (Fig. 8o and Supplementary Fig. S15f). Additionally, we observed increased nuclear expression of NF-κB P65 protein in airway epithelial cells of COPD patient lung slices, compared with that of non-smokers and smokers in the healthy group (Supplementary Table S4 and Supplementary Fig. S16a–d). Together, NETs components are linked to dysregulated NF-κB-dependent cytokines, but not type-I IFNs, in BALF of COPD patients.

## Discussion

NETs are initially recognised to entrap and kill pathogens via a DNA framework comprising abundant antimicrobial granular proteins, such as MPO and NE.^31^ However, they are immunogenic and have been implicated in several autoimmune and non-infectious diseases.^25,32,33^ In COPD, augmented self-DNA from increased lung cell apoptosis acts as DAMPs, impeding inflammation resolution. NETs, rich in DNA content, have been implicated in the loss of microbiota diversity among patients with COPD^20^ and CS-induced acute airway inflammation in murine models.^23,30^ Nevertheless, the role of NETs in mediating innate and adaptive immune responses to sustain airway inflammation in COPD remains unknown. Herein, we demonstrated the immunomodulatory role of NETs in long-term CS-induced COPD as follows: (1) hPBNs derived from patients with COPD exhibited a stronger ability to release CSE-NETs, which contained high levels of oxidatively-damaged mtDNA and chDNA; (2) CSE-NETs not only stimulated the proliferation and production of both NF-κB-dependent cytokines and type-I IFNs on hAECs, but also promoted the maturation of hDCs; (3) these effects were ameliorated by the silencing/inhibition of cGAS and TLR9 in vitro; (4) in vivo, the knockout of DNA sensors (*cGAS*^*−/−*^ and *TLR9*^*−/−*^ mice), the inhibition of NETs (by mitoTEMPO i.p), and the degradation of NETs (by DNase-I *Neb*.) respectively resulted in alleviated airway inflammation, reduced production of NF-κB-dependent cytokines (but not type-I IFNs), suppressed NF-κB P65 activation, and improved lung function in the long-term CS-induced COPD mouse model; (5) the severity of NETs infiltration was correlated with that of airway inflammation in the CS-treated mice; and (6) In COPD patients, the levels or activity of NETs components (MPO and NE activity) in BALF correlate with disease severity and the abundance of NF-κB-dependent cytokines (IL-1β and CXCL8), but not those of type-I IFN (IFN-β). Taken together, the DNA components in CSE-NETs promoted NF-κB-, but not type-I IFNs-, dependent autoimmunity via cGAS/TLR9 in long-term CS-induced COPD.

The cause of excessive NETosis in COPD is largely unknown;^34^ despite spontaneous NETosis regulated by CXCR2 in patients with COPD,^35^ NETosis induced by CS exposure might be a more likely contributor. Notably, we observed a stronger capacity for NETosis in COPD-neutrophils upon CSE stimulation. Consistently, COPD-neutrophils demonstrated increased NETosis following exposure to diesel exhaust,^36^ thus suggesting greater susceptibility to undergo NETosis following exposure, potentially linked to augmented mtROS release in COPD-neutrophils. Unlike PMA-induced NETosis that relies on NOX, CSE-triggered NETosis responds primarily to mtROS. Consistently, ribonucleoprotein (RNP)-induced NETosis required mtROS instead of NOX in patients with systemic lupus erythematosus,^37^ thereby indicating that NETosis induced by different inducers may rely on dissimilar mechanisms.^38^ Moreover, CSE-NETs consist of abundant oxidatively-damaged DNA, which is more capable of promoting autoimmune reactions^33^ and the formation of autologous NETs.^39^ Furthermore, CSE-NETs comprised a lower ratio of mtDNA to chDNA compared to PMA- and iomomycin-NETs, since mtDNA exhibits different immunoregulatory features from chDNA,^40^ NETs induced by different stimuli may exert diverse cellular effects. This variability could account for the controversial effects of PMA- and IL-8-induced NETs on IL-8/IL-6 expression in hAECs.^25,27,28^ Accordingly, we investigated the effects of CSE-NETs on the function of hAECs, which are essential for maintaining immune homoeostasis in the lungs.^41^ CSE-NETs stimulated proliferation, production of both NF-κB-dependent cytokines and type-I IFNs, and NF-κB (P65 and P50) activation in hAECs. Moreover, both cGAS and TLR9 were required to facilitate these effects, thus emphasising the importance of NETs-DNA sensing on hAECs to mediate an autoimmune response. Previously, CSE-NETs were shown to promote DC maturation to prime T-cell-mediated immune response,^22,24^ and we further showed that inhibition of cGAS and TLR9 suppressed the CSE-NETs-induced maturation of hDCs. Taken together, CSE-NETs triggered hAEC and hDC dysfunction, and the sensing of NETs-DNA by both airway structural cells (such as hAECs) and immune cells (such as hDCs) promoted autoimmune reactions.

TLR4 and TLR9 are critical PPRs involved in COPD pathogenesis.^42–44^ TLR4 recognises exogenous ligands (such as lipopolysaccharide), respiratory syncytial virus fusion protein, and endogenous ligands like heat shock proteins (HSP60, HSP70) and high mobility group box 1 (HMGB1), whereas TLR9 specialises in the detection of unmethylated CpG motifs prevalent in bacterial and viral DNA, as well as in human mtDNA.^45^ cGAS is a crucial innate immune sensor that detects cytoplasmic DNA from pathogens or cellular injury. Both cGAS and TLR9 are reported DNA sensors capable of sensing cytosolic/extracellular mtDNA/chDNA.^46,47^ The recognition of self-DNA by cGAS and TLR9 not only activates the NF-κB signalling pathway to release NF-κB-dependent cytokines but also induces the robust production of type-I IFNs.^15^ These cytokines and IFNs serve as prominent immunomodulators in autoimmune contexts.^48,49^ Excessive activation of cGAS/TLR9 has been linked to allergic and autoimmune diseases.^50,51^ For example, sensing of cytosolic DNA by cGAS in hAECs^52^ and TLR9 in innate lymphoid cells^53^ plays a role in asthma pathogenesis. However, the role of NETs-DNA recognition by cGAS/TLR9 in eliciting NF-κB-dependent cytokines and type-I IFNs response in COPD is unclear. Interestingly, our in vitro data showed that CSE-NETs stimulated type-I IFNs production in hAECs, while in vivo data suggested that CS exposure resulted in decreased type-I IFNs levels in both CS-treated mice and patients with COPD; moreover, only levels of NF-κB-dependent cytokines, but not type-I IFNs, were correlated with disease severity in COPD mouse models and patients with COPD. This controversy between in vitro and in vivo results might stem from the impairment of IFNAR1 expression on hAECs, a crucial receptor of type-I IFNs to facilitate the downstream signalling cascade,^54^ by long-term CS exposure (supplementary Fig. S6f),^55^ thus hindering the overall amplification and production of type-I IFNs. Notably, the decreased expression of type-I IFNs has been previously reported in COPD contexts, the suppressed type-I IFNs in COPD might compromise the antiviral defence ability, thus contributing to persistent airway inflammation and even acute exacerbations.^56,57^ These findings emphasised the key role of NETs-DNA and the associated NF-κB signalling, but not type-I IFNs response, in sustained airway inflammation in COPD.

The interplay and differences between cGAS and TLR9 in their downstream signalling upon DNA recognition remain poorly understood. In hDCs, the pathways regulated by cGAS and TLR9 may exist in parallel and display interdependencies to suppress each other.^58^ Notably, mitochondrial DNA itself has been shown to activate NETosis via the cGAS-stimulator of interferon genes (STING) axis, with enhancing NE production and extracellular DNA release within NETs during sterile inflammation,^39^ suggesting a functional involvement of cGAS-STING in NETosis. Therefore, it is possible that NETs-DNA could stimulate a feedback loop in neutrophils by further promoting NETosis under CS exposure conditions. Consistently, we observed significantly less NETs infiltration in CS-treated cGAS^−/−^ (but not TLR9^−/−^) mice, indicating that cGAS, but not TLR9, participates in CS-induced NETosis. Consequently, *cGAS* deficiency might not only reduce NETs-DNA sensing but also suppress CS-induced NETosis, thus providing potentially superior protection against CS exposure than *TLR9* deficiency. Regarding NETs-DNA sensing in hAECs, although silencing *cGAS* or *TLR9* individually led to varied reductions in CSE-NETs-induced cytokines/IFNs expressions/productions, no significant differences emerged from two-way ANOVA analysis (supplementary Fig. S14). Nevertheless, further investigation into the intricate regulatory roles that cGAS and TLR9 play in DNA sensing and response is warranted, to better clarify their differences in downstream signalling upon DNA recognition.

MitoTEMPO is a superoxide inhibitor that specifically targets the mitochondria.^59^ We observed a desirable control of CS-induced COPD following mitoTEMPO treatment probably owing to the following reasons: (1) mitoTEMPO efficiently inhibits CSE-induced NETosis; (2) mitoTEMPO helps in restoring the equilibrium of oxidant and antioxidant molecules,^60^ which are characterised in CS-induced COPD;^61^ (3) oxidatively-damaged mtDNA/chDNA is more capable of inducing the disordered release of cytokines, which is likely controlled by mitoTEMPO. Previously, DNase-I was shown to protect mice from type-2 immunopathology^33^ and CS-induced acute airway inflammation,^23^ we further showed that the degradation of NETs-DNA by DNase-I treatment alleviated NF-κB-dependent cytokines and emphysema-phenotype in the long-term CS-treated mice, suggesting that DNase-I could offer modest protective effects against the sustained airway inflammation and subsequent alveolar destruction. It should be noted that the therapeutic potential of DNase-I might be limited by the suboptimal nebulised administration methods used in this study. Nevertheless, we further showed that the severity of NETs infiltration was correlated with that of airway inflammation and emphysema, as well as the levels of NF-κB-dependent cytokines in the CS-treated mice, thus supporting the importance of NETs-DNA in CS-induced airway inflammation and alveolar destruction. In summary, our in vivo data demonstrate that sensing-blockage/inhibition/degradation of NETs-DNA confers therapeutic benefits in the long-term CS-induced COPD mouse model. However, when translating these observations to clinical practice, it is essential to acknowledge the complexity of COPD pathophysiology, where targeting NETs or cGAS/TLR9 alone might not be sufficient to fully address the intricate interactions between innate and adaptive immunity, DAMPs, cytokines, and concurrent pathogen challenges. Potential synergistic benefits in reducing inflammation and improving lung function could be achieved through combination therapies targeting multiple aspects of COPD, including but not limited to NETosis and cGAS/TLR9 pathways. Moreover, given the critical role of cGAS/TLR9 signalling in pathogen recognition and antiviral responses, as well as NETs’ function in pathogen clearance and lung microbiota maintenance, targeted interventions must be judiciously designed to minimise disruption of host defence mechanisms.

Our study has limitations: while we establish correlations between NETs components, disease severity, and NF-kB-dependent cytokines levels in BALF of patients with COPD adhering to standard LAMA or ICS + LABA therapies, we lack information on individual pharmacological regimens that might impact MPO and NE levels. This is particularly pertinent given previous studies suggesting an association between NETs abundance and ICS resistance or ICS regular use in patients with asthma,^62,63^ implying potential interactions between NETosis and ICS therapy. Future research could benefit from incorporating detailed clinical records to elucidate how current therapeutic strategies influence CS-induced NETosis.

Herein, we present evidence that CS-induced NETs-DNA sensed by cGAS and TLR9 in AECs and DCs promotes NF-κB-, but not type-I IFNs, dependent autoimmunity in long-term CS-induced COPD. Despite their roles in eliminating pathogens and maintaining microbiota diversity in the lungs, NETs contribute self-DNA as a significant DAMP, promoting disordered production of inflammatory cytokines and thus sustaining airway inflammation in an autoimmune fashion in COPD. Consequently, NETs-DNA and its sensing receptors (cGAS/TLR9) represent potential therapeutic targets to reduce persistent airway inflammation in COPD. Supplementary Fig. S17 illustrates the proposed mechanism.

## Materials and methods

### Study approval and ethics statements

The protocol of human study was reviewed and approved by the Chinese Ethics Committee for Registering Clinical Trials (approval number: ChiCTR900022271). All animal experiments were approved and conducted in accordance with the guidelines of the Animal Ethics Committee of the West China Hospital (approval number: 2018049A).

### Patients and samples

COPD diagnosis adhered to the Global Strategy for the Diagnosis, Management, and Prevention of COPD (GOLD) criteria. From January 2016 to March 2020, the study recruited 45 healthy individuals and 42 COPD patients at West China Hospital, all providing informed consent. Among them, 11 healthy volunteers and 10 COPD patients contributed peripheral blood samples for NETs and DCs experiments. Additionally, 34 healthy participants (divided into 13 non-smokers and 21 smokers) and 32 COPD patients with a history of smoking underwent BALF testing (Supplementary Tables S1, S2). The collections of BALF, circulating neutrophils, and monocytes were described in the supplementary materials.

### Neutrophils isolation and NETs stimulation

Neutrophils from COPD patients and healthy participants were isolated using the MACSxpress Neutrophil Isolation Kit (Miltenyi Biotec, Cat No. 130-104-434) according to the manufacturer’s instructions, with purity confirmed by flow cytometry for CD15 and CD16. Freshly isolated cells (1.0 × 10^6^) were seeded on fibrinogen-coated coverslips and incubated in serum-free RPMI 1640 for adhesion. Subsequently, NETs were induced by varying concentrations of CSE at 37 °C for 4 to 24 h to assess CSE-induced NETosis; PMA and ionomycin stimulation were used as positive control. We administered mitoTEMPO, TTFA, DPI, VAS, DNase-I, and GW as described in the supplementary materials.

### Quantification of NETs-releasing neutrophils

The evaluation of NETs-releasing neutrophils percentage was conducted by immunofluorescence co-staining of the following NETs components: myeloperoxidase (MPO), histone H3, and DNA. Briefly, NETs on coverslips were washed twice with phosphate buffer saline (PBS), permeabilized with ice-cold acetone:methanol (−20 °C), fixed in neutral formalin, then blocked and incubated overnight at 4 °C with primary antibodies for MPO and histone H3. After PBS washes, secondary antibodies were applied for 2 h. Following mounting with DAPI medium, samples were imaged on whole-filed fluorescence microscopy and evaluated as described in the supplementary materials.

### Collection of NETs-containing supernatants

CSE-induced NETs were prepared under 5% CSE for 18 h, which induces approximately 50% NETosis without neutrophil necrosis. After CSE exposure, NETs were thoroughly washed and detached with MNase (Thermo Fisher Scientific, Cat No. 88216, 20 units/mL, 37 °C, 30 min). DNA concentration was quantified using the QuantiFluor dsDNA system (Promega, Cat No. E2670). NETs were enriched and sequenced by next-generation sequencing as described in the supplementary materials.

### Culture and simulation of hAECs

Primary hAECs were obtained from Lifeline Cell Technology (Cat No. FC-0016) and maintained in BrochiaLife Medium Complete Kit (Cat No. LL-0023) on collagen-coated plates. Cells at passages 3–5 were subjected to 6, 12 or 24 μg/mL NETs for 48 or 72 h. RNA and protein extraction ensued using E.Z.N.A. HP Total RNA Isolation Kit (Omega Bio-Tek, Cat No. R6812) and Minut Total Protein Extraction Kit (Invent Biotechnologies, Cat No. SD-001/SN-002), respectively. Proliferation was assessed via Click-iT EdU Assay (Invitrogen, C10499). cGAS and TLR9 expression in hAECs was silenced using small interfering RNA (siRNA) (Life Technologies) transfected with TransIT-TKO Reagent (Mirus Bio, *Cat No*. MIR 2150), with transfection efficiency and cytotoxicity monitored using BLOCK-iT Alexa Fluor Red Control (Invitrogen, Cat No. 14750100). Optimal siRNA sequences (Cat No. S28872 for cGAS, Cat No*.* S41746 for TLR9) were applied prior to NETs stimulation. The supplementary materials described the detailed methods of cell culture, stimulation, siRNA knockdown of *cGAS* and *TLR9*, quantitation of proliferation, reverse-transcription quantitative PCR (RT-qPCR), western blotting on primary hAECs.

### Differentiation and stimulation of peripheral monocytes-derived DCs

Monocytes were isolated from fresh peripheral blood of healthy donors or COPD patients via lymphoprep density gradient medium (StemCell Technologies, *Cat No*. 07801). Briefly, blood diluted with PBS containing 2% FBS (StemCell Technologies, Cat No. 07905) was centrifuged at 800×*g* for 30 min. Monocytes were harvested and differentiated into DCs using the ImmunoCult Dendritic Cell Culture Kit (StemCell Technologies, Cat No. 10985) at 37 °C for 3 days, and further differentiated for 2 days with fresh medium to yield immature DCs. These cells were then stimulated with 12 μg/mL NETs for 24 h at 37 °C for subsequent flow cytometry analysis. ImmunoCult Dendritic Cell Maturation Supplement (StemCell Technologies, Cat No. 10985) was employed as a positive control for DC maturation. To explore cGAS and TLR9’s role in NETs-induced DC maturation, RU.521 (Aobious Inc., *Cat No*. AOB37877, a selective cGAS inhibitor), and ODN 2088 (InvivoGen, tlr1-2088, a recognised TLR9 antagonist disrupting CpG ODNs and TLR9 interaction) were employed. The detailed methods of differentiation, stimulation, and flow cytometry assessment of monocyte-derived DCs were described in the supplementary materials.

### Animal experiments

We obtained cGAS knockout mice (*cGAS*^*−/−*^, *Cat No*. 026554) and TLR9 knockout mice (*TLR9*^*−/−*^, Cat No. 034329) from Jackson Laboratories. The COPD mouse model was established using an established nose-only CS exposure method.^64^ Briefly, 8-week-old, sex-matched mice were placed in custom nose-only exposure tubes (China pattern No. ZL201821367875.5) for smoke inhalation. Smoke from Marlboro cigarettes (1.0 mg nicotine, 11 mg tar) was generated using a smoking machine (CH Technologies) and diluted with fresh air. Mice were exposed to smoke from ~30 cigarettes in two 75-min sessions per day, 5 days a week, for 12 weeks, interspersed with recovery periods. MitoTEMPO (Sigma-Aldrich, Cat No. SML0737) was prepared at 0.5 mg/mL in sterile saline, filtered and administered intraperitoneally (50 μg in 100 μL) to 25 g mice on smoking days, with saline as control. DNase-I (Sigma-Aldrich, Cat No. 11284932001) was dissolved to 2 mg/mL; 4 mL dissolved DNase-I containing 16,000 units was aerosolized over 15 min on smoking days, and saline was used for controls. We then assessed lung function, NETs infiltration, histology, cell counts in BALF, and levels of proinflammatory cytokines in serum and BALF as described in the supplementary materials.

### Quantification of NETs infiltration in lung slice of mouse

The NETs in lung sections of mice were immunofluorescence co-stained with MPO, histone H3 and DNA. Lung sections underwent dewaxing, hydration, and antigen retrieval in citrate buffer at 95 °C. Autofluorescence reduction was achieved with sodium borohydride incubation, followed by PBS washing. Post-blocking, primary antibodies for MPO and histone H3 were applied overnight at 4 °C, followed by secondary antibodies (Alexa Fluor 555 and 647) for 2 h. Slides were washed, mounted with DAPI medium, and stored at 4 °C. Negative controls were included. Imaging was conducted on a Zeiss Imager Z2 microscope. ImageJ was used to analyse fluorescence images. Briefly, the lung areas in images were measured, followed by demarcation of NETs areas and background areas from negative controls. Normalised NETs areas were calculated as the average percentage of NETs areas minus background areas across three random views per slice, with detailed methods described in the supplementary materials.

### Assessment of NF-κB-dependent-cytokines, type-I IFNs levels

Cytokine and interferon levels in mouse serum and BALF were determined using the Bio-Plex Pro Mouse Chemokine Luminex Assay Kit (BioRad, Lot No. 17005875). Human BALF analysis was executed with the Magnetic Luminex Screening Assay Kit (R&D systems, Cat No. LXSAHM) on a Bio-Plex 200 system (BioRad) following standard procedures as detailed in the supplementary materials. In vitro, hAECs were stimulated with TLR9/cGAS siRNA and NETs, and culture supernatants post-centrifugation were stored at −80 °C. ELISA (R&D Systems, Cat No. VAL101, VAL103 and VAL137) quantified IL-1β, CXCL8 and IFN-β levels per manufacturer instructions.

### DNA-binding ELISAs for activated NF-κB P65 and P50

Nuclear extracts of hAECs stimulated with TLR9/cGAS siRNA and/or NETs were prepared with a nuclear extract kit (Active Motif, Cat No. 40410). Whole-cell extracts of frozen lung tissues from *TLR9*^*−/−*^, *cGAS*^*−/*−^ and control mice post-CS exposure were also obtained. Protein content was quantified via Bradford assay (BioRad, Cat No. 5000201). NF-κB P65 and P50 activities were assessed using NF-κB P65 (or P50) Activity Assay (Active Motif, Cat No. 40096 and 41096) according to the manufacturer’s instructions as described in the supplementary materials.

### Statistical analysis

Data are expressed as mean ± SD. Statistical details, including analysis methods, significance thresholds (*P* < 0.05), *n* values, scatter plots, and *p* values, are presented within figure legends and supplementary materials. Briefly, for two-group comparisons, data normality was assessed via Shapiro–Wilk or Kolmogorov–Smirnov tests; normally distributed datasets were analysed by unpaired *t*-tests, while non-normal datasets underwent Mann–Whitney *U*-tests. For multiple-group comparisons, one-way or two-way ANOVA, complemented by Tukey’s honest significant test, were employed. For partial correlation analysis, Pearson’s partial correlation tests controlled for covariates (age, sex, BMI, smoking history of COPD patients), succeeded by multiple linear regression analyses.

### Supplementary information

Supplementary Materials

Supplementary Movie S1

## Data Availability

The raw sequence data have been deposited in the Genome Sequence Archive in National Genomics Data Center, China National Center for Bioinformation/Beijing Institute of Genomics, Chinese Academy of Sciences, under accession number HRA001059 that are publicly accessible at https://ngdc.cncb.ac.cn/gsa-human. Other data were available from the supplemental material, or from the corresponding author upon request.
