# Supplementary material for: DNA of neutrophil extracellular traps promote NF-κB-dependent autoimmunity via cGAS/TLR9 in chronic obstructive pulmonary disease
Source: Signal Transduct Target Ther. 2024 Jun 17;9:163. doi: 10.1038/s41392-024-01881-6 (PMC11180664; doi:10.1038/s41392-024-01881-6)
Supplement: Supplementary file 1 — Supplementary Materials [file 41392_2024_1881_MOESM1_ESM.docx]

Supplementary Materials for

DNA of neutrophil extracellular traps promote NF-κB-dependent autoimmunity via cGAS/TLR9 in COPD

Jun Chen; Tao Wang; Xiaoou Li; Lijuan Gao; Ke Wang; Mengxin Cheng; Zijian Zeng; Lei Chen; Yongchun Shen; Fuqiang Wen

Correspondence to: [wenfuqiang@scu.edu.cn](mailto:wenfuqiang@scu.edu.cn)

**This PDF file includes:**

Materials and Methods

Figures S1 to S18

Tables S1 to S5

Caption for Movie S1

**Other Supplementary Materials for this manuscript include the following:**

Movie S1

Materials and Methods

1. ***Inclusion and exclusion criteria of patients with chronic obstructive lung disease (COPD) and healthy participants***

All protocols were conducted in accordance with the amended Declaration of Helsinki, and approved by the Chinese Ethics Committee of Registering Clinical Trials with an approval number of ChiCTR900022271. COPD was diagnosed according to the Global Initiative for COPD (GOLD) criteria ^1^. While the situations outlined below were excluded: 1), complicated with respiratory diseases other than COPD, including asthma, acute respiratory distress syndrome and lung cancer; 2), received any antibiotics within 8 weeks; 3), unable or unwilling to cooperate or perform the spirometry. Based on the above criteria, a total of 45 healthy participants and 42 patients with COPD were recruited at the West China Hospital from January 2016 to March 2020, with informed consent provided and following characteristics collected and reviewed: age, sex, body mass index (BMI) and smoking history, detailed medical history and a chest CT taken in recent 5 years. Patients with COPD enrolled were adherent to either guideline-directed treatment with a long-acting muscarinic antagonist (LAMA) alone or dual therapy consisting of an inhaled corticosteroid combined with a long-acting beta-2 agonist (ICS + LABA), which are established therapeutic modalities in COPD management. All patients with COPD had no past medical history of prolonged oral glucocorticoid use. The characteristics of all participants were summarized in supplementary Table S1 and S2. Among them, 11 healthy participants and 10 patients with COPD donated peripheral blood for neutrophil extracellular traps (NETs) and dendritic cells (DCs) associated assays, while 34 healthy participations (including 13 non-smokers and 21 smokers) and 32 patients with COPD with smoking history received bronchoalveolar lavage fluid (BALF) tests.

1. ***Spirometry***

All recruits received a standard spirometry according to the European Respiratory Society guideline ^2^. Patients with COPD were diagnosed if: forced expiratory volume in 1s (FEV_1_) < 80% predicted and FEV_1_ / forced vital capacity (FVC) < 70% in the post-bronchodilator spirometry (Fig. 8, supplementary Fig. S15 and supplementary Table S1).

1. ***Collection and preparation of BALF of participations***

BALF specimen was obtained following a standard protocol as previously described ^3^. Briefly, a bronchofiberoscope was wedged in the segmental bronchus of right middle lobe, 4 portions of 0.9% sterile saline were instilled and recollected, then aliquoted and stored immediately at -80℃ for further tests (Fig. 8 and supplementary Fig. S15).

1. ***Isolation and enrichment of peripheral neutrophils from participations***

The neutrophils were isolated from the peripheral blood of patients with COPD and healthy participants using MACSxpress neutrophil isolation kit according to the manufacturer's manual (*Cat No.* 130-104-434, Miltenyi Biotec, Auburn, CA, USA). The magnetic beads-based untouched isolation method avoided possible stimulations of neutrophils. Briefly, the erythrocytes are aggregated and sedimented, the non-target cells are removed by immunomagnetic depletion after incubation with magnetic beads, then yielding untouched and functional neutrophils with high recovery rate (supplementary Fig. S2a). The purity of isolated neutrophils was further confirmed by the flow cytometry with immunofluorescence labeling of antibodies against CD15 and CD16 (supplementary Fig. S2b, the detailed methods were described below in “Flow cytometry”).

1. ***Cigarette smoke extract (CSE) preparation***

CSE was freshly prepared as described previously ^4^. Briefly, three cigarettes (Marlboro, Philips Morris International Inc., Lausanne, Switzerland; 1.0 mg nicotine and 11 mg tar per cigarette) was drawn slowly and bubbled through 10 mL of RPMI 1640 medium (Corning, NY, USA) to yield 100% CSE, which was further titrated to pH 7.4 and sterilized. 1%, 5%, 25%, 50% CSE were diluted by certain volume of serum-free RPMI 1640 medium from 100% CSE.

1. ***Stimulation and inhibition of CSE-induced NETosis***

1.0×10^6^ neutrophils freshly isolated from the peripheral blood of patients with COPD and heathy participants were seeded on coverslip precoated with 2.5 mg/mL fibrinogen (*Cat No.* F3879, Sigma-Aldrich, St. Louis, MO, USA) and incubated with serum-free RPMI 1640 medium in 24-well plate for 30 min for adhesion. Then the culture medium was replaced by 1%, 5%, 25%, 50% CSE respectively, and further incubated at 37 ℃ for 4, 8, 18, 24 h respectively to elucidate the capacity of CSE to induce NETosis, a process of NETs release (Fig. 1, supplementary Fig. S2 and supplementary Movie S1). Alternatively, 50 nM PMA and 4 μM lonomycin, two classic NETs inducers ^5, 6^, were used to stimulate NETosis (incubation for 4 h) as positive control. In some experiments, 50 μM mitoTEMPO (a mitochondrially targeted antioxidant, *Cat No.* SML0737, Sigma-Aldrich), 50 μM thenoyltrifluoroacetone (TTFA, a mitochondrial respiration inhibitor, *Cat No.* 10-4565, Focus Biomolecules, Plymouth Meeting, PA, USA), 50 μM diphenyleneiodonium chloride (DPI, a NADPH oxidase inhibitor, *Cat No.* BML-CN240, Enzo Life Science, Farmingdale, NY, USA), 50 μM VAS2870 (VAS, a NADPH oxidase inhibitor, *Cat No.* SML0273, Sigma-Aldrich), 50 μM GW311616A (a selective human neutrophil elastase inhibitor, *Cat No.* G2364AM, CFW Laboratories Inc., Walnut, CA, USA), 200 units/mL deoxyribonuclease I (DNase-I, an endonuclease for single- or double-stranded DNA, *Cat No.* 11284932001, Sigma-Aldrich) were given respectively to evaluate their effects on the NETosis of peripheral neutrophils (derived from both patients with COPD and healthy participants), which were induced by either CSE 5% or PMA 50 nM at 37 ℃ for 18 h (Fig. 2, supplementary Fig. S3 and S4).

1. ***Quantification of NETosis by immunofluorescence co-staining of NETs components***

The percentage of neutrophils to release NETs was evaluated by immunofluorescence labeling of three main components in NETs: DNA, Myeloperoxidase (MPO) and Histone H3, which was recommended as a reliable approach to distinguish the NETs (Fig. 1a-f, 2a-f, supplementary Fig. S2d-j, S3 and S4a-f) ^7^. Briefly, NETs attached on the coverslip was washed completely with Phosphate Buffer Saline (PBS, Corning) twice, then permeabilized with 1:1 acetone and methyl alcohol (precooled at -20 ℃) for 5 min, and fixed with 10% neutral formaldehyde for 10 min. After washed with PBS, NETs were incubated with blocking buffer (10% donkey serum) at 37 ℃ for 30 min, and stained with primary antibodies against MPO (1:50, *Cat No.* AF3667, R&D Systems, Minneapolis, MN) and histone H3 (1: 50, *Cat No.* ab5103, Abcam, Cambridge, UK) diluted in blocking buffer at 4 ℃ overnight. The coverslips were washed with PBS 3 times and further stained with secondary antibodies Alexa Fluor 555 and Alexa Fluor 647 (1:200, Invitrogen Corporation, Carlsbad, CA, USA) respectively at room temperature for 2 h (supplementary Fig. S2c). Negative control with the absence of either primary antibody or primary + secondary antibody was also prepared to rule out the nonspecific stanning. After washed off the secondary antibodies with PBS 3 times, the coverslip was mounted with anti-fade mounting medium with DAPI (*Cat No.* ab104139, Abcam) on a glass slide, and stored at 4 ℃ for further evaluation. The fluorescence images were obtained by a whole-filed ﬂuorescence microscopy (Imager Z2, Zeiss, Oberkochen, Germany). The representative immunofluorescence co-staining images of CSE-induced NETs (with negative controls) were given in Figure 1a and supplementary Fig. S3a. The representative immunofluorescence co-staining images showing the inhibitory effects of different compounds on NETosis were given in supplementary Fig. S3b.

1. ***Fluorescence staining of oxidatively damaged DNA, mitochondrial outer membrane, and mitochondrial ROS in vitro***

In some experiments, oxidized DNA were labeled by antibody of biotinylated 8-hydroxy-2'-deoxyguanosine (8-OHdG, 1: 50, *Cat No.* ab62623, Abcam), while the mitochondrial outer membrane was labeled by antibody of TOMM20 (1: 50, *Cat No.* ab186734, Abcam) (Fig. 2j-k, supplementary Fig. S4h). The coverslips were washed with PBS 3 times and further stained with secondary antibodies Alexa Fluor 555 and Alexa Fluor 647 (1:200, Invitrogen Corporation, Carlsbad, CA, USA) respectively at room temperature for 2 h. Negative control with the absence of either primary antibody or primary + secondary antibody was also prepared to rule out the nonspecific stanning. After washed off the secondary antibodies with PBS 3 times, the coverslip was mounted with anti-fade mounting medium on a glass slide, and stored at 4 ℃ for further evaluation. The fluorescence images were obtained by a whole-filed ﬂuorescence microscopy (Imager Z2, Zeiss, Oberkochen, Germany), while the laser confocal images were obtained by a laser scanning confocal microscope (A1RMP+, Nikon Corporation, Tokyo, Japan).

For the assessment of mitochondrial ROS induced by CSE (Fig. 2g-i, supplementary Movie S1), neutrophils freshly isolated from the peripheral blood of patients with COPD and heathy participants were treated with CSE 5 % at 37 ℃ for 1, 2 or 4 h as described above, then stained with MitoSOX Red Mitochondrial Superoxide Indicator (*Cat No.* M36008, Invitrogen Corporation) as per the manufacturer’s instruction, and observed and recorded by an inverted ﬂuorescence microscopy (Observer D1/AX10 cam HRC, Zeiss).

Alternatively, neutrophils were co-stained with mitochondrial ROS indicator (MitoSOX Red) and intracellular ROS indicator Dichlorodihydrofluorescein diacetate (DCFH-DA) staining (*Cat No.* S0033S, Beyotime Biotechnology) according to manufacturer’s instruction as previously reported ^8^. The confocal images were obtained by laser scanning confocal microscopy (supplementary Fig. S4g).

1. ***Collection of NETs-containing supernatants in vitro***

CSE-NETs were collected for further stimulation of cells. Human peripheral neutrophils were treated with 5% CSE for 18 h to induce NETs, this experimental condition was selected based on previous observations, wherein incubation with 5% CSE for 18 hours led to NETosis by approximately 50%, without inducing necrosis in the neutrophils. Briefly, NETs were induced by 5% CSE for 18h as described above and washed 3 times carefully with PBS to remove CSE completely. NETs were further detached by incubation with 20 units/mL of micrococcal nuclease (MNase, *Cat No.* 88216, Thermo Fisher Scientific, Waltham, MA, USA) at 37 ℃ for 30 min.

MNase is an enzyme that cleaves inter-nucleosomal linker DNA to fragment chromatin. The length of the fragments depends on the concentration of MNase and the duration of the incubation. It has been reported that both nucleosomes and DNA prepared by MNase induced IL-1β in human monocytes, and the maximal induction was achieved using a 0.2 kb DNA fragment compared to larger fragments ^9^, indicating that cleaved nucleosomes and DNA prepared by MNase are capable of inducing inflammation. In our study, we used a concentration of MNase (20 units/mL) and an incubation time (30 min) consistent with previous studies ^10, 11^.

Specifically, NETs were detached by incubation at 37 ℃ for 30 min with 20 units/mL micrococcal nuclease diluted in either BrochiaLife Basal-Medium supplemented with BronchiaLife Life-Factors (BrochiaLife Medium Complete Kit, Cat No. LL-0023, Lifeline Cell Technology, Frederick, MD, USA) or ImmunoCult-ACF Dendritic Cell medium with differentiation supplement (ImmunoCult Dendritic Cell Culture Kit, Cat No. 10985, StemCell Technologies, Vancouver, Canada). The NETs-containing supernatants were centrifuged at 350 × *g* for 5 min in 4 ℃ and collected as 100% NETs, then stored at -40 ℃ for further usage and analysis.

The concentration of 5% and 10% CSE-NETs in supernatant was quantified by fluorescence quantitative assay (supplementary Fig. S5a) as per the manufacturer’s instruction (QuantiFluor dsDNA system, *Cat No.* E2670, Promega, Madison, WI, USA). Briefly, a standard curve prepared using dsDNA standards, together with 5% or 10% NETs prepared from at least 3 independent assays as described above, were stained with QuantiFluor dsDNA Dye. The intensity of florescence (504nm_EX_/531nm_EM_) was measured to reflect the concentration of dsDNA using a plate reader (SpectraMax i3, Molecular Devices, San Jose, CA, USA).

1. ***Next-generation sequencing of CSE-induced NETs***

High-quality DNA components in NETs was enriched and purified using DNeasy Blood & Tissue Kit (*Cat No.* 69504, Qiagen, Hilden, Germany) as per the manufacturer’s instruction. Samples were sequenced via 150 nt paired-end run on an illumine Hiseq Xten as provided and performed by Basebiotech Co. Ltd (Chengdu, China). The reads were mapped to the human transcriptome. All software was parallelized and run on an internal high performance-computing cluster. Heat map visualization of the sequencing data were generated using the method described previously ^12^. The RPKIM (reads per kilobase per million mapped reads) ratio of mitochondria derived DNA and chromatin DNA were calculated (Fig. 1g-h). The raw sequence data have been deposited in the Genome Sequence Archive ^13^ in National Genomics Data Center ^14^, China National Center for Bioinformation / Beijing Institute of Genomics, Chinese Academy of Sciences, under accession number HRA001059 that are publicly accessible at <https://ngdc.cncb.ac.cn/gsa-human>.

1. ***Cell culture, stimulation, EdU proliferation assay of human airway epithelial cells (hAECs)***

Normal primary hAECs were purchased from Lifeline Cell Technology (*Cat No.* FC-0016) and cultured in BrochiaLife Basal-Medium supplemented with BronchiaLife Life-Factors (BrochiaLife Medium Complete Kit, *Cat No.* LL-0023, Lifeline Cell Technology) in 12, 24 and 96-well plate pre-coated with 80 μg/mL type-I rat tail collagen (*Cat No.* 5153, Advanced Biomatrix, San Diego, CA, USA). 3-5 passages of primary hAECs were treated with 6, 12 and 24 μg/mL NETs for 48 and 72 h respectively, then the total mRNA and protein of hAECs were extracted using E.Z.N.A. HP Total RNA Isolation Kit (*Cat No.* R6812, Omega Bio-Tek, Doraville, GA, USA) and Minut Total Protein Extraction Kit (*Cat No.* SD-001/SN-002, Invent Biotechnologies, Plymouth, MN, USA) according to the manufacturer’s instruction (Fig. 3b-g, 3j-k, Fig. 4b-g, 4j-k, 4n-s, 4v-w, supplementary Fig. S5c-f, S6a, S6c, S6e-f, S14b-g, S14j-k).

Based on the findings of above experiments, we determined that the concentration of 12μg/mL (but not the highest 24 μg/mL) was optimal for the subsequent experiments (supplementary Methods 12), as it significantly enhanced both proliferation and cytokines production of hAECs.

In some experiments, the proliferation ability of hAECs treated with NETs was assessed by Click-i EdU Proliferation Assay for Microplates (*Cat No.* C10499, Invitrogen Corporation) following the manufacturer’s instruction (Fig. 3a, Fig. 4a and 4m, supplementary Fig. S14a).

1. ***Silence of cyclic GMP-AMP synthase (cGAS) and Toll-like receptor 9 (TLR9) on hAECs***

We utilized small interfering RNA (siRNA) to silence the expression of cGAS and TLR9 on hAECs. Briefly, two different sequences for cGAS siRNA (Silencer Select, *Cat No.* S28872 and S28873, Life Technologies, Carlsbad, CA, USA), TLR9 siRNA (Silencer Select, *Cat No.* S41746 and S41747, Life Technologies) and negative control siRNA (Silence Select Negative Control No. 1 siRNA, *Cat No.* 4390843, Life Technologies) were designed and synthesized by Life Technologies. The transfections were carried out using the TransIT-TKO Transfection Reagent (*Cat No.* MIR 2150, Mirus Bio LLC, Madison, WI, USA), and the efficiency and cytotoxic of transfection reagent was verified using BLOCK-iT Alexa Fluor Red Fluorescent Control (*Cat No.* 14750100, Invitrogen Corporation) as per the manufacturer’s instruction. 10 nM of cGAS, TLR9 and negative control siRNA were pre-prepared with transfection reagent and given to the hAECs for 24 h at 37 ℃ before stimulation with NETs-containing supernatants. The most efficient cGAS siRNA (*Cat No.* S28872, Life Technologies) and TLR9 siRNA (*Cat No.* S41746, Life Technologies) was used in the following experiments (Fig. 4a-x, supplementary Fig. S6a-e). The silence efficiency of cGAS and TLR9 siRNA were verified by assessing the mRNA and protein expression of hAECs (supplementary Fig. S5c-f).

1. ***Reverse-transcription quantitative PCR***

The total mRNA and protein of hAECs were extracted using E.Z.N.A. HP Total RNA Isolation Kit (*Cat No.* R6812, Omega Bio-Tek, Doraville, GA, USA). 1 μg RNAs were transcribed reversely into complementary DNAs (cDNA) using PrimeScript RT reagent Kit with gDNA Eraser (*Cat No.* RR047A, Takara Biotechnology, Dalian, China), followed by reverse-transcription quantitative PCR (RT-qPCR) assay with a SYBR Green master mix (Fast Start Essential DNA Green Master, *Cat No.* 6402712001, Roche, Basel, Switzerland) using a Light Cycler 96 real-time PCR system (Roche) as described previously ^15^. Relative levels of each transcript to GAPDH were calculated with 2^-∆Ct^ [∆Ct = Ct _(gene of interest)_ – Ct _GAPDH_]. The primers for most of genes used in this study were designed by PrimerBank (<https://pga.mgh.harvard.edu/primerbank/>) and synthesized by TsingKe Biological Technology (Chengdu, China); the primers for the homo-16s and homo-18s were reported previously ^16, 17^; the specificity and efficiency of primers were tested by real-time PCR experiments before usage, the detailed information of primers can be found in supplementary Table S5.

1. ***Western blot***

The total protein of hAECs were extracted using Minut Total Protein Extraction Kit (*Cat No.* SD-001/SN-002, Invent Biotechnologies, Plymouth, MN, USA) following the manufacturer’s instruction. The quantification of protein expression were described previously ^18^. Briefly, the total proteins were fractionated by electrophoresis with 10% SDS polyacrylamide gel, the fractionated proteins were transferred to polyvinylidene fluoride (PVDF) membranes and blocked with 5 % bovine serum albumin (BSA) in Tris-buffered saline at room temperature for 1 h, then incubated with antibodies against cGAS (1:500, *Cat No.* 15102S, Cell Signaling Technology, Danvers, MA, USA), TLR9 (1:500, *Cat No.* ab134368, Abcam), P65 (1:1000, *Cat No.* 8242S, Cell Signaling Technology), phosphorylated P65 (p-P65, 1:1000, *Cat No.* 3031S, Cell Signaling Technology), P50 (1:500, *Cat No.* 66992-1-Ig, Proteintech), IκB-α (1:1000, *Cat No.* 4814S, Cell Signaling Technology) and IFN-α/β receptor Subunit-1 (IFNAR1, 1:1000, *Cat No.* ab124764, Abcam), Lamin B1, GAPDH and β-actin (1:1000, *Cat No.* 250010, 200306 and 200068, Zen-Bioscience, Chengdu, China) overnight at 4 ℃, then incubated with horseradish peroxidase (HRP)-linked secondary antibodies (1:2000, Cell Signaling Technology). The secondary antibodies were excited by SuperSignal West Pico Chemiluminescent substrate (*Cat No.* 34077, Pierce, Rockford, IL, USA) and visualized by Tanon 5200 Chemiluminescent Imaging System (Tanon Science & Technology, Shanghai, China). The intensity of bands was further quantified (supplementary Fig. S5e-f, S6a, S6c, S6e-f) by ImageJ (National Institutes of Health, Bethesda, MD, USA). Alternatively, the nuclear extracts of hAECs were separated using Nuclear Extract Kit (Cat No. 40410, Active Motif) according to the manufacturer’s instruction. The fractionation efficiency of nuclear extracts was determined by nucleus marker Lamin B1 and cytoplasm marker GAPDH.

1. ***Differentiation and stimulation of peripheral monocytes-derived human DCs***

As the frequency of DCs in peripheral blood was extremely low, the peripheral monocytes were collected and differentiated into DCs by using ImmunoCult Dendritic Cell Culture Kit (*Cat No.* 10985, StemCell Technologies, Vancouver, Canada). Briefly, the mononuclear cells (MNCs) were isolated from fresh peripheral bloods of either healthy donators or patients with COPD using density gradient medium Lymphoprep (*Cat No.* 07801, StemCell Technologies). The diluted blood with equal amount of PBS with 2 % fetal bovine serum (FBS, *Cat No.* 07905, StemCell Technologies) was layered on the top of Lymphoprep and centrifuged at 800 × *g* for 30 min at room temperature. The MNCs were collected at the interface of plasma and Lymphoprep, and incubated with ImmunoCult DC differentiation Medium (ImmunoCult-ACF Dendritic Cell Medium mixed with ImmunoCult ACF Dendritic Cell Differentiation Supplement provided by the kit, *Cat No.* 10985, StemCell Technologies) in 12-well plate at 37 ℃ for 3 days, then further incubated with replaced medium for additional 2 days to obtain the differentiated immature DCs. These cells were further stimulated with 12 μg/mL NETs as described above at 37 ℃ for 24 h, and then harvested for further assessment by flow cytometry (Fig. 3m-n). Meanwhile, the ImmunoCult Dendritic Cell Maturation Supplement provided by the kit (*Cat No.* 10985, StemCell Technologies) was also given to induce maturation of DCs as a positive control.

1. ***Inhibition of cGAS and TLR9 activity on hDCs***

To investigate the role of cGAS and TLR9 in the maturation of DCs induced by NETs, we used a published selective inhibitor of cGAS ^19^, RU.521 (*Cat No.* AOB37877, Aobious Inc., Gloucester, MA, USA), to inhibit the activity of cGAS, and a well-known TLR9 antagonist, ODN 2088 (*Cat No.* tlr1-2088, InvivoGen, San Diego, CA, USA), to disrupt the colocalization of CpG ODNs with TLR9 ^20^. RU.521 was firstly dissolved in dimethyl sulfoxide (DMSO, *Cat No.* D2650, Sigma-Aldrich), and then prepared at 5 μM solution in ImmunoCult DC differentiation Medium (*Cat No.* 10985, StemCell Technologies) with 0.1% DMSO as final concentration; ODN 2088 was prepared at 2 μM solution in ImmunoCult DC differentiation Medium directly. The dose selections were based on the preliminary studies and literature ^20, 21^. The solutions of inhibitors were given to the immature DCs derived from healthy donators for 2 h, then the DCs were further stimulated with 12 μg/mL NETs in the presence of RU.521 and ODN 2088 for 24 h (Fig. 4y-z). The control group were treated with the culture medium with or without 0.1% DMSO. The NETs-treated DCs with or without inhibitors were harvested for further assessment by flow cytometry as described below.

1. ***Flow cytometry***

The maturation of DCs was characterized by flow cytometry (Fig. 3m-n, 4y-z) as described previously ^22^. Briefly, the harvested DCs were incubated with 2.5 μg Human BD Fc Block (*Cat No.* 564220, BD Biosciences, San Diego, CA, USA) in staining buffer for 10 min at room temperature, then co-incubated with antibodies against surface molecules CD304 (Alexa Fluor 647, *Cat No.* 566047, BD Biosciences), HLADR (PerCP-Cy 5.5, *Cat No.* 560652, BD Biosciences), CD40 (BB515, *Cat No.* 565258, BD Biosciences) and CD86 (phycoerythrin, C, *Cat No.* 555665, BD Biosciences) protected from light for 30 min at 4 °C; the single-stained cells, unstained cells, and cells stained with corresponding isotype controls (*Cat No.* 557691, 550927, 564416, 555743, BD Biosciences) were prepared as controls; the nonviable cells were excluded by 7-AAD Viability Staining Solution (*Cat No.* 420403, BioLegend, Cambridge, UK). The flow cytometry assays were performed on FACSAria II (BD Biosciences).

In other experiments, to assess the purity of neutrophils (supplementary Fig. S2b), the isolated neutrophils were treated with RBC Lysis Buffer (*Cat No.* 420301, BioLegend), then co-incubated with antibodies against CD15 (PerCP-Cy 5.5, *Cat No.* 323020, BioLegend) and CD16 (PE, *Cat No.* 302008, BioLegend), the isotype controls (*Cat No.* 400149, 400113, BioLegend) were also used to ensure the specificity of signals.

In some cases, to assess the expression of mitochondrial ROS on neutrophils (Fig. 2g-i), the isolated neutrophils treated with or without 5 % CSE for 2 h were stained with MitoSO Red Mitochondrial Superoxide Indicator (PE, *Cat No.* M36008, Invitrogen Corporation) as per the manufacturer’s instruction. The data analysis and plotting were further performed using FlowJo software (Tree Star Inc., USA).

1. ***Animals***

cGAS knockout (*cGAS^-/-^*, *Cat No.* 026554) and TLR9 knockout (*TLR9^-/-^*, *Cat No.* 034329) mice were obtained from Jackson Labs (Bar Harbor, ME, USA), the knockout efficacy of target gene in these strains have been widely verified (See the Jackson Labs website: https://www.jax.org/strain/026554 and https://www.jax.org/strain/014534); The wild type C57Bl/6J mice were obtained from GemPharmatech Co. Ltd (Nanjing, Jiangsu, China). Mice were allocated randomly into different groups by an experimenter who did not know the details of experiments and was not involved in the following experiments. The groups allocation, experiment conduction and data analysis were performed by different experimenters. All mice were age and sex-matched and bred in a specific pathogen free environment at the Animal Experimental Center of West China hospital.

All animal experiments were approved and conducted in accordance with the guideline of Animal Ethics Committee of West China hospital with an approval number: 2018049A. The sample size of animal experiments meets the statistical requirement, and varies from 7 to 20 as some mice died during long-term CS exposure, all mice survived were included in the final statistical analysis, the detailed sample size for each experiment were reported in figure legends.

1. ***Cigarette smoke (CS) induced COPD mouse model***

A well-established COPD mouse model was obtained by a nose-only CS exposure method (treated for 12 weeks), this mouse model presents clearly airflow limitation- and emphysema-phenotype similar to that induced by whole-body CS exposure for 6 months ^3, 23, 24^. The pathological manifestations and the detailed apparatus and methods used for the establishment of this mouse model has been described previously ^3^ and widely acknowledged among different studies ^15, 25, 26^.

Briefly, 8 weeks-old and sex-matched mice were restrained in a custom-designed nose-only exposure tubes (China Pattern number: ZL201821367875.5), which allow smoke or air going through the nose of mouse in a one-way flow. A commercially available cigarette (Marlboro, Philips Morris, USA; 1.0 mg nicotine and 11 mg tar per cigarette) was lit by a cigarette-smoking machine (CH Technologies, West-Wood, NJ, USA) to generate the cigarette smoke, which was further diluted by fresh air with positive pressure using a flow-fixed pump (CH Technologies, West-Wood, NJ, USA). The concentration of smoke was monitored regularly and determined by the ratio of total particulate matter weight in smoke to the total flow volume (mg/m^3^). The mice were subjected to 75 min exposure of smoke generated from ~30 cigarettes for each session, 2 sessions a day separated by a recovery period, 5 days a week, for total 12 weeks. The control mice were exposed to fresh air using the same protocol.

1. ***Administration of mitoTEMPO and DNase-I in vivo***

For *in vivo* administration of mitoTEMPO (*Cat No.* SML0737, Sigma-Aldrich), mitoTEMPO was firstly dissolved in sterile saline to prepare 0.5 mg/mL mitoTEMPO solution, which was further filtrated with 0.22 μm filters (*Cat No.* 16541, Sartorius Stedim, Goettingen, Germany). For a mouse with 25 g bodyweight, 50 μg mitoTEMPO (100 μL solution) was given via intraperitoneal injection (*i.p*) on the days of cigarette smoking. The control mice were treated with the same amount of saline.

For *in vivo* nebulisation of DNase-I (*Cat No.* 11284932001, Sigma-Aldrich), DNase-I (2000 units/mg) was firstly dissolved in sterile saline to prepare 2 mg/mL DNase-I solution. 4 mL DNase-I solution containing 16000 units DNase-I was nebulised to mice for 15 min on the days of cigarette smoking by using an atomization system specifically designed for mouse (YLS-8B, Yiyan Scientific Co. Ltd, Shandong, China). The control mice were treated with the same amount of saline by using similar method. The outline and grouping for the experiments of COPD mouse model were summarized in supplementary Fig. S7.

1. ***Assessment of*** ***mouse lung functions***

Mice were anesthetized with 50 mg/kg pentobarbital sodium (*Approved No.* F 20020915, SCRC-Hushi, Shanghai, China) via *i.p*, then tracheostomized and set up in a forced pulmonary maneuver system (Buxco Max II, Buxco Electronics, NY, USA). The maneuver of Boyle’s Low functional residual capacity (FRC), Quasistatic pressure-volume (PV) and Fast Flow Volume were performed and repeated at least three time ^27^. Briefly, the FRC was determined by the Boyle’s Law FRC maneuver; the total lung capacity (TLC) were acquired by the Quasistatic PV maneuver; the forced vital capacity (FVC), forced expiratory volume (FEV) at 100 ms (FEV_100_) were recorded in the Fast Flow Volume maneuver (Fig. 5k-l, 6k-l, 7k-l, supplementary Fig. S10k-l).

1. ***Collection and preparation of mouse serum and BALF, and cell counts in BALF***

The protocol has been described in detail previously ^3, 18^. Briefly, the mouse was exsanguinated from the right ventricle to collect the blood into a coagulation-promoting tube, which was centrifugated at 3000 × g for 10 min, then the serum was collected and immediately frozen at −80 °C for further assessment. The right lung of mouse was lavaged with 0.5 mL sterile ice-cold PBS supplemented with a Protease Inhibitor Cocktail (*Cat No.* HY-K0011, MCE, Monmouth Junction, NJ, USA) for 3 times to collect BALF, which was centrifuged at 1000 × g at 4 °C for 5 min, the supernatant was immediately frozen at −80 °C for further assessment. The cell pellet was treated with Red Blood Cell Lysis Buffer (*Cat No.* C3702, Biyuntian Biotechnology, Shanghai, China), and further resuspended with 500 μL PBS. The total cell counts in BALF were performed by a hemocytometer (Moxi Z mini cell counter, Orflo Technologies, Ketchum, ID, USA), while the differential cell counts were determined by cell smears stained with Wright–Giemsa Stain (*Cat No.* D010, Jiancheng Biotech, Nanjing, China), and 5 random views in each slice were recorded by using Nikon 80i microscope (Fig. 5b-d, 6b-d, 7b-d, supplementary Fig. S10b-d).

1. ***Histology of mouse lung slices***

The left lung of mouse without lavage was fixed by 4% phosphate buffered paraformaldehyde, embedded in paraffin, and sectioned into 4-µm-thick slices, which were stained with hematoxylin and eosin (H&E) solution for morphological examination, and alcian blue-periodic acid schiff stain for mucin evaluation. The histologic inflammatory score and mucin stain score were evaluated by an individual blinded to the details of experimental grouping (Fig. 5m-n, 6m-n, 7m-n, supplementary Fig. S9b, S10m-n, S11b, S12b, S13b). The histologic inflammatory score for each mouse was determined by evaluating the perivascular infiltration, peribronchial infiltration, parenchymal infiltration, and epithelial damage: from no inflammation (score = 0) to profound infiltration/damage (score = 5) for each parameter as described previously ^28^; The mucin stain score for each mouse was determined by evaluating the level of staining: from no staining (score = 0) to high level of staining (score = 5) as described previously ^29^. The alveolar enlargement was determined by mean linear intercept (MLI), a ratio of total length of alveoli to the number of alveoli per field (supplementary Fig. S9a, S11a, S12a, S13a).

1. ***Immunofluorescence co-staining and quantification of NETs in lung slice of mouse***
2. ***Antigen retrieval and autofluorescence quenching***

The method for the immunofluorescence co-staining of NETs in mouse lung slice has been reported previously ^30^. The paraffin embedded lung slices were firstly dewaxed, deparaffinized, rehydrated and subjected to antigen retrieval treatment by incubation in 0.01M citrate buffer (pH 6.0) at 95°C for 10min, then treated with sodium borohydride (SB, *Cat No.* 686018, Sigma-Aldrich), an effective quencher to diminish the autofluorescence of lung slices ^31^. The lung slices were immersed in the SB solution prepared at 1 mg/mL in pre-cooled PBS for 3 consecutive 10 min without intermediary wash, and finally washed by PBS.

1. ***Immunofluorescence co-staining of NETs in lung slice of mouse***

After incubated with blocking buffer (10% donkey serum) for 2 h at room temperature, the lung slices were stained with primary antibodies against MPO (1:50, *Cat No.* AF3667, R&D Systems) and histone H3 (1: 50, *Cat No.* ab5103, Abcam) diluted in blocking buffer at 4 ℃ overnight. The coverslips were washed with PBS 3 times and further stained with secondary antibodies Alexa Fluor 555 and Alexa Fluor 647 (1:200, Invitrogen Corporation) at room temperature for 2 h (supplementary Fig. S2c). After washed off the secondary antibodies with PBS 3 times, the lung slices were mounted with anti-fade mounting medium with DAPI (*Cat No.* ab104139, Abcam), and stored at 4 ℃. A similar slide from consecutive serial sections of each mouse lung was prepared with the absence of primary antibody, and regarded as a negative control to rule out the background stanning. 3 random views in each lung slice of mouse, together with 3 similar views in corresponding negative control lung slice, were obtained by a whole-filed ﬂuorescence microscopy (Imager Z2, Zeiss, Oberkochen, Germany) using the same exposure parameters. The representative immunofluorescence co-staining images of NETs in the mouse lung slice was given in supplementary Fig. S8.

1. ***Quantification of NETs*** ***in lung slice of mouse***

The fluorescence images were further analyzed by ImageJ. Briefly, the whole areas of lung structure as indicated by phase contrast image of lung slice were firstly calculated (as “Whole areas”). Then the co-staining areas were extracted and regarded as the areas of infiltrated NETs (as “NETs areas”). The areas in negative control lung slices obtained under the same exposure parameters and extraction method were also recognized and regarded as “Background areas”. Therefore, the normalized areas of infiltrated NETs in lung slices were calculated (Fig. 5o-p, 6o-p, 7o-p, supplementary Fig. S10o-p) as followed:

Average percentage of NETs areas = 100% × (NETs areas _view1_ / Whole areas _view1_ ＋ NETs areas _view2_ / Whole areas _view2_ ＋ NETs areas _view3_ / Whole areas _view3_) / 3

Average percentage of background areas = 100% × (Background areas _view1_ / Whole areas _view1_ ＋ Background areas _view2_ / Whole areas _view2_ ＋ Background areas _view3_ / Whole areas _view3_) / 3

Normalized areas of NETs = Average percentage of NETs areas － Average percentage of background areas

1. ***Immunofluorescence and immunohistochemical staining of NF-κB P65, CXCL5, IL-1β and IFN-β in lung tissue slices of human participants and mouse model***

6 Healthy participants (including 3 nonsmokers and 3 smokers) and 3 patients with COPD (with smoking history) who underwent solitary pulmonary nodule (SPN) resection with confirmed benign pathology were recruited at the West China Hospital from April 2023 to July 2023. All recruits meet the inclusion and exclusion criteria as described in Method 1, and patients with COPD were diagnosed as described in Method 2, the characteristics of participants were summarized in supplementary Table S4. Sample of lung parenchyma located at least 5 cm from the nodular lesion were obtained and collected immediately in ice-cold 4% phosphate buffered paraformaldehyde for fixation, followed by paraffin embedding and slicing.

Immunofluorescence staining was performed by using a commercial Treble-Fluorescence immunohistochemical mouse/rabbit kit (*Cat No.* RS0035, Immunoway) with principle of tyramide signal amplification (TSA) as previously described ^32^. Briefly, lung slices underwent dewaxing, rehydration and antigen retrieval as described in Method 24, then permeabilized with 1:1 acetone and methyl alcohol (precooled at -20 ℃) for 5 min. Unspecific binding of antibodies was blocked by peroxidase blocking solution for 30 min before each staining cycle. Primary antibody against P65 (1:400, *Cat No.* 8242S, Cell Signaling Technology) were given to lung slices for 2 h, followed by incubation with HRP-labeled rabbit secondary antibodies for 20 min, then TSA opal fluorophore (Opal 647) for 10 min at room temperature. Subsequently, anti-Uteroglobin (CC10, 1:1000, *Cat No.* ab307666, Abcam) conjugated to Opal 488 was used to identify airway epithelial cells. The antibody-TSA complex was removed by a heat-mediated antigen retrieval process using Tris/EDTA buffer between staining cycles. Then lung slices were counterstained with DAPI (*Cat No.* P36931, Invitrogen). Negative control was prepared by staining lung slice with vehicle instead of primary antibodies to rule out spontaneous and non-specific signals. The immunofluorescence confocal images (supplementary Fig. S16a) were obtained by laser scanning confocal microscope (A1RMP+, Nikon Corporation, Tokyo, Japan). The ratio of DAPI and P65 co-stained area (indicating expression of P65 in nucleus) to the airway epithelium area for each lung slice was quantified by ImageJ (supplementary Fig. S16b).

Alternatively, immunohistochemical staining for proteins of P65, CXCL5, IL-1β and IFN-β were performed using ImmunoCruz ABC Staining System Kit (*Cat No.* sc-2018, Santa Cruz Biotechnology) as previously described ^33^. Briefly, lung tissues from human participants (n = 3 for each group as described above and summarized in supplementary Table S4) and COPD mouse model with or without treatment (n = 6 for each group of animal experiments, supplementary Fig. S7, 6 archived lung tissues were randomly selected from each group) were sectioned and prepared according to manufacturer’s instruction. Lung slices were stained with antibody for P65 (1:50, *Cat No.* 8242S for human only, Cell Signaling Technology, supplementary Fig. S16c), CXCL5 (ENA-78, 1:150, *Cat No.* YT5961 for both human and mouse, Immunoway, supplementary Fig. S9c, S11c, S12c, S13c and S16e), IL-1β (1:300, *Cat No.* 26048-1-AP for both human and mouse, Proteintech, supplementary Fig. S9d, S11d, S12d, S13d and S16h), IFN-β (1:200, *Cat No.* 27506-1-AP for both human and mouse, Proteintech, supplementary Fig. S9e, S11e, S12e, S13e and S16k), respectively, as per the manufacture’s instruction. Thress random views (under 60 X) focusing on airway epithelium in each slice were recorded by using Nikon 80i microscope. The area of airway epithelium was extracted and measured by using deconvoluted DAB image in ImageJ as previously reported ^34^, then normalized across groups. Airway epithelial cells with staining of P65, CXCL5, IL-1β and IFN-β respectively in nucleus were considered as positive cells. Average “positive cell counts per normalized area of airway epithelium” in each slice was calculated, and quantified by using 3 (for human participations) or 6 (for mice) slices in each group. Similarly, the gray values of the immunohistochemical staining of CXCL5, IL-1β and IFN-β were measured in deconvoluted DAB image by ImageJ, then divided by the normalized area of airway epithelium and presented as “mean gray value per normalized area of airway epithelium”.

1. ***Assessment of cytokines, interferons levels and neutrophil elastase (NE) activity***

The cytokines and interferons levels in serum and BALF of mice were assessed by a customized Bio-Plex Pro Mouse Chemokine Luminex Assays Kit (*Lot No.* 17005875, BioRad) as per the manufacture’s instruction (Fig. 5e-j, 6e-j, 7e-j, supplementary Fig. S9n-p, S9r-s, S10e-j, S11n-p, S11r-s, S12n-p, S12r-s, S13n-p, S13r-s); The cytokines and interferons levels in BALF of participations were measured using a customized Magnetic Luminex Screening Assay Kit (*Cat No.* LXSAHM, R&D systems) on a Bio-Plex 200 Luminex Analyzer System (BioRad, Munich, Germany) as per the manufacture’s instruction (Fig. 8). Briefly, the analyte-specific antibodies are pre-coated onto magnetic microparticles to bind the cytokines and interferons of interest. After washing, a biotinylated antibody cocktail specific to the analytes of interest were added, followed by incubation with streptavidin-PE conjugate, which binds to the biotinylated antibody. The microparticles were then resuspended in buffer and read by Bio-Plex 200 Luminex Analyzer System. All fluorescence emissions from each microparticle were recognized and analyzed in a high-throughput, multiplex way.

NE activity in BALF of participations were measured by Neutrophil Elastase Activity Assay (*Cat No.* ab204730, Abcam) according to manufacturer’s instruction (supplementary Fig. S15a-f). Briefly, a fresh set of elastase enzyme standards and diluted BALF samples were prepared. The ability of NE was assessed by proteolytically cleaving synthetic substrate to release fluorophore. The outputs were recorded at Ex/Em = 380/500 nm in a kinetic mode, every 3 minutes, for 21 minutes at 37℃ by using a fluorescent microplate reader (SpectraMax i3, Molecular Devices, San Jose, CA, USA). △Relative fluorescence unit (RFU) = (RFU _Time 2_ – RFU _Background 2_) – (RFU _Time 1_ – RFU _Background 1_). The standard curve was built according to the concentrations of elastase enzyme standards and corresponding △RFU_380/500 nm_. The activity of NE is then calculated by: NE activity = The amount of NE / original sample volume (ng/mL).

Alternatively, to assess cytokine and interferon levels in cell culture supernatants, primary hAECs were treated with or without TLR9/cGAS siRNA and/or NETs. Cell culture supernatants were collected and centrifuged at 300 g for 10 min, then stored at -80℃. Concentration of IL-1β, CXCL8 and IFN-β in supernatants were measured by human IL-1β/CXCL8/IFN-β Valukine Enzyme-linked Immunosorbent Assay (ELISA) kit (*Cat No.* VAL101, VAL103 and VAL137, R&D Systems) according to manufacturer’s instructions (Fig. 3h-i, 3l, 4h-i, 4l, 4t-u, 4x, supplementary Fig. S14h-i, S14l).

1. ***DNA-binding ELISAs for activated*** ***NF-κB transcription factors***

Primary hAECs treated with or without TLR9/cGAS siRNA and/or NETs were lysed, and nuclear extracts were collected using nuclear extract kit (*Cat No.* 40410, Active Motif); Alternatively, whole-cell extracts from frozen lung tissues of *TLR9^-/-^*or *cGAS^-/-^* mice or corresponding littermates treated with or without CS exposure were collected. Protein concentration of nuclear extracts and whole-cell extracts were measured by Bradford protein assay (*Cat No.* 5000201, BioRad). The activity of NF-κB transcription factors P65 and P50 were quantified using the TransAM NF-κB P65 (or P50) Activity Assay (*Cat No.* 40096 and 41096, Active Motif) according to manufacturer’s instructions (supplementary Fig. S5b, S6b, S6d, S9q, S11q, S12q and S13q). Briefly, active NF-κB P65 or P50 in nuclear extracts (10 μg protein/sample) or whole-cell extracts (20 μg protein/sample) were captured by a consensus binding sequence of oligonucleotide coated on a 96-well plate, then further detected by primary anti-P65 or anti-P50 antibody, followed by a secondary antibody conjugated to HRP, and finally quantified as colorimetric readout by a spectrophotometry (SpectraMax i3, Molecular Devices, San Jose, CA, USA).

1. ***Statistical analysis***

Supplementary Fig. S1 depicts a schematic of the overall study design. Statistical parameters including statistical analysis, statistical significance, and n values are stated in the figure legends and supplementary figure legends, the scattered samples and the p values are also displayed in the figures. All data were reported as mean ± SD; significant different was considered when P<0.05.

The data were analyzed and plotted by IBM SPSS Statistics Version 26.0.0.0 (IBM Corp., Armonk, NY, USA) or GraphPad Prism Version 9.00 (GraphPad Software Inc., San Diego, CA, USA) as described below or in the figure legends and supplementary figure legends:

1. For the analysis of two group comparison, the distribution of data was firstly tested by Shapiro-Wilk normality test or KS normality test, then the data were compared using Unpaired t test or Mann-Whitney test according to the distributions.
2. For multiple comparisons, one-way or two-way ANOVA analysis of variance followed Tukey’s honestly significant difference analysis (Tukey’s HSD) were performed.
3. For partial correlation analysis in human cohort and animal model, Pearson’s partial correlation test was performed by controlling for age, sex, body mass index (BMI), and smoking history of the patients with COPD, followed by multiple linear regression analysis.

**Reference**

1 Global Strategy for the Diagnosis, Management, and Prevention of COPD. Available from <http://goldcopd.org/>. *Global Initiative for Chronic Obstructive Lung Disease* (2016).

2 Miller M*, et al.* Standardisation of spirometry. *Eur Respir J*; **26:** 319-338 (2005).

3 Chen J*, et al.* The elevated CXCL5 levels in circulation are associated with lung function decline in COPD patients and cigarette smoking-induced mouse model of COPD. *Ann Med*; **51:** 314-329 (2019).

4 Guo L*, et al.* WNT/β-catenin signaling regulates cigarette smoke-induced airway inflammation via the PPARδ/p38 pathway. *Lab Invest*; **96:** 218-229 (2016).

5 Kenny E*, et al.* Diverse stimuli engage different neutrophil extracellular trap pathways. *eLife*; **6:** e24437 (2017).

6 Hoppenbrouwers T*, et al.* In vitro induction of NETosis: Comprehensive live imaging comparison and systematic review. *PLoS One*; **12:** e0176472 (2017).

7 Jorch S, Kubes P. An emerging role for neutrophil extracellular traps in noninfectious disease. *Nat Med*; **23:** 279-287 (2017).

8 Ding MG*, et al.* Mfn2-mediated mitochondrial fusion alleviates doxorubicin-induced cardiotoxicity with enhancing its anticancer activity through metabolic switch. *Redox Biol*; **52:** 1 (2022).

9 Tsourouktsoglou TD*, et al.* Histones, DNA, and Citrullination Promote Neutrophil Extracellular Trap Inflammation by Regulating the Localization and Activation of TLR4. *Cell Rep*; **31:** 107602 (2020).

10 Barrientos L*, et al.* Neutrophil extracellular traps downregulate lipopolysaccharide-induced activation of monocyte-derived dendritic cells. *J Immunol*; **193:** 5689-5698 (2014).

11 Qiu S*, et al.* Neutrophil extracellular traps induced by cigarette smoke activate plasmacytoid dendritic cells. *Thorax*; **72:** 1084-1093 (2017).

12 Pavlidis P, Noble W. Matrix2png: a utility for visualizing matrix data. *Bioinformatics*; **19:** 295-296 (2003).

13 Wang Y*, et al.* GSA: Genome Sequence Archive. *Genomics, Proteomics Bioinf*; **15:** 14-18 (2017).

14 DRNGDC. Database Resources of the National Genomics Data Center, China National Center for Bioinformation in 2021. *Nucleic Acids Res*; **49:** D18-D28 (2021).

15 Yang T*, et al.* Serotonin receptors 5-HTR2A and 5-HTR2B are involved in cigarette smoke-induced airway inflammation, mucus hypersecretion and airway remodeling in mice. *Int Immunopharmacol*; **81:** 106036 (2020).

16 Reithofer M*, et al.* Alum triggers infiltration of human neutrophils ex vivo and causes lysosomal destabilization and mitochondrial membrane potential-dependent NET-formation. *FASEB J*; **34:** 14024-14041 (2020).

17 Apel F*, et al.* The cytosolic DNA sensor cGAS recognizes neutrophil extracellular traps. *Sci Signaling*; **14:** eaax7942 (2021).

18 Yang D*, et al.* Mitoquinone ameliorates cigarette smoke-induced airway inflammation and mucus hypersecretion in mice. *Int Immunopharmacol*; **90:** 107149 (2021).

19 Vincent J*, et al.* Small molecule inhibition of cGAS reduces interferon expression in primary macrophages from autoimmune mice. *Nat Commun*; **8:** 750 (2017).

20 Römmler F*, et al.* Guanine modification of inhibitory oligonucleotides potentiates their suppressive function. *J Immunol*; **191:** 3240-3253 (2013).

21 Wiser C, Kim B, Vincent J, Ascano M. Small molecule inhibition of human cGAS reduces total cGAMP output and cytokine expression in cells. *Sci Rep*; **10:** 7604 (2020).

22 He S*, et al.* Bronchial epithelial cell extracellular vesicles ameliorate epithelial-mesenchymal transition in COPD pathogenesis by alleviating M2 macrophage polarization. *Nanomedicine*; **18:** 259-271 (2019).

23 Jones B*, et al.* Animal models of COPD: What do they tell us? *Respirology*; **22:** 21-32 (2017).

24 Fricker M, Deane A, Hansbro PM. Animal models of chronic obstructive pulmonary disease. *Expert Opin Drug Discov*; **9:** 629-645 (2014).

25 Beckett EL*, et al.* A new short-term mouse model of chronic obstructive pulmonary disease identifies a role for mast cell tryptase in pathogenesis. *J Allergy Clin Immunol*; **131:** 752-762 (2013).

26 Shu J*, et al.* Comparison and evaluation of two different methods to establish the cigarette smoke exposure mouse model of COPD. *Sci Rep*; **7:** 15454 (2017).

27 Vanoirbeek J*, et al.* Noninvasive and invasive pulmonary function in mouse models of obstructive and restrictive respiratory diseases. *Am J Respir Cell Mol Biol*; **42:** 96-104 (2010).

28 Chen J*, et al.* A novel peptide ADAM8 inhibitor attenuates bronchial hyperresponsiveness and Th2 cytokine mediated inflammation of murine asthmatic models. *Sci Rep*; **6:** 30451 (2016).

29 Linden SK, Florin TH, McGuckin MA. Mucin dynamics in intestinal bacterial infection. *PLoS One*; **3:** e3952 (2008).

30 Toussaint M*, et al.* Host DNA released by NETosis promotes rhinovirus-induced type-2 allergic asthma exacerbation. *Nat Med*; **23:** 681-691 (2017).

31 Davis A*, et al.* Characterizing and Diminishing Autofluorescence in Formalin-fixed Paraffin-embedded Human Respiratory Tissue. *J Histochem Cytochem*; **62:** 405-423 (2014).

32 Zeng ZJ*, et al.* Inherent differences of small airway contraction and Ca^2+^ oscillations in airway smooth muscle cells between BALB/c and C57BL/6 mouse strains. *Front Cell Dev Biol*; **11** (2023).

33 Yang T*, et al.* Quercetin attenuates airway inflammation and mucus production induced by cigarette smoke in rats. *Int Immunopharmacol*; **13:** 73-81 (2012).

34 Cizkova K, Foltynkova T, Gachechiladze M, Tauber Z. Comparative Analysis of Immunohistochemical Staining Intensity Determined by Light Microscopy, ImageJ and QuPath in Placental Hofbauer Cells. *Acta Histochem Cytochem*; **54:** 21-29 (2021).


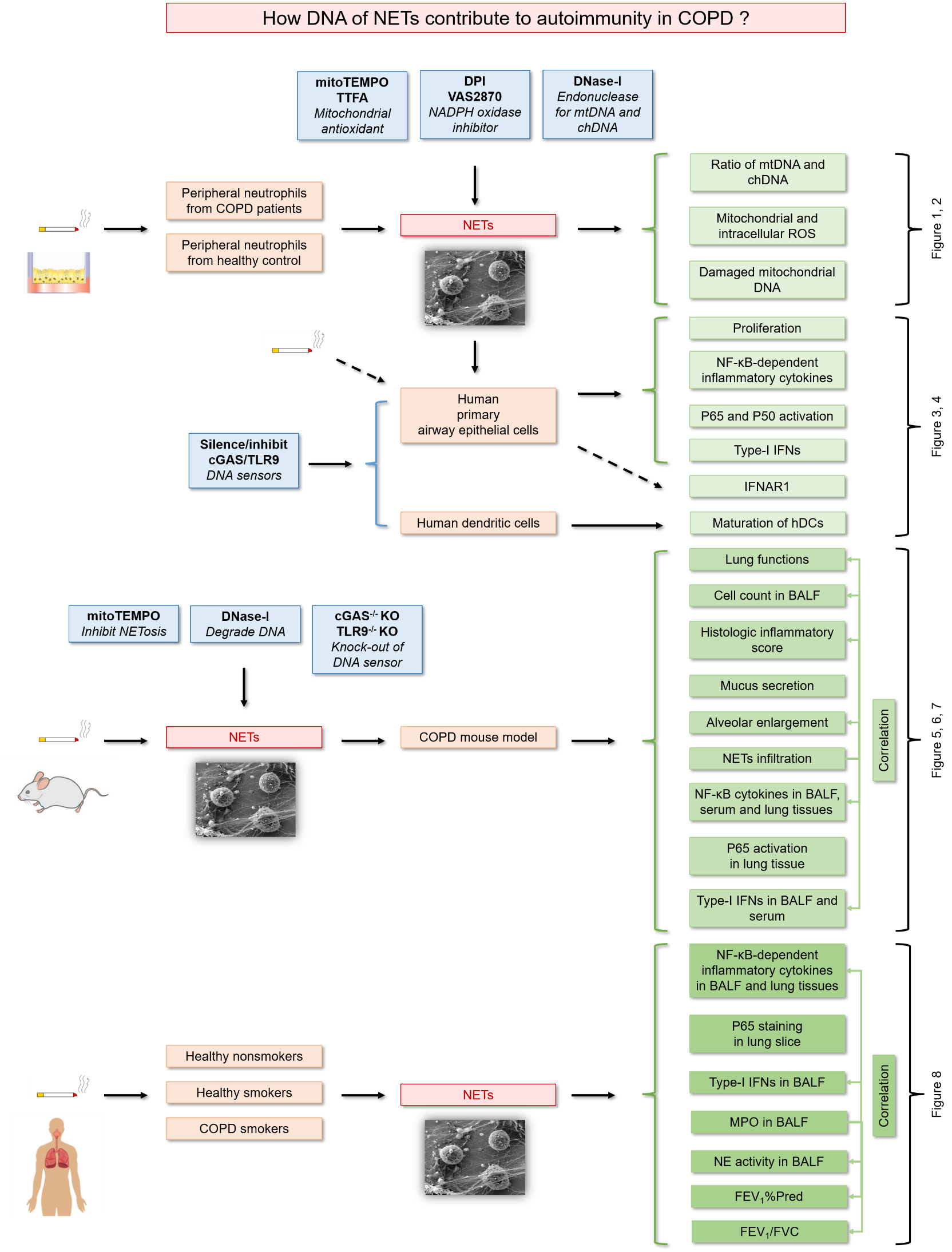
**Supplementary figures S1 to S18**

**Figure S1.** A schematic diagram displays the overall design of study.


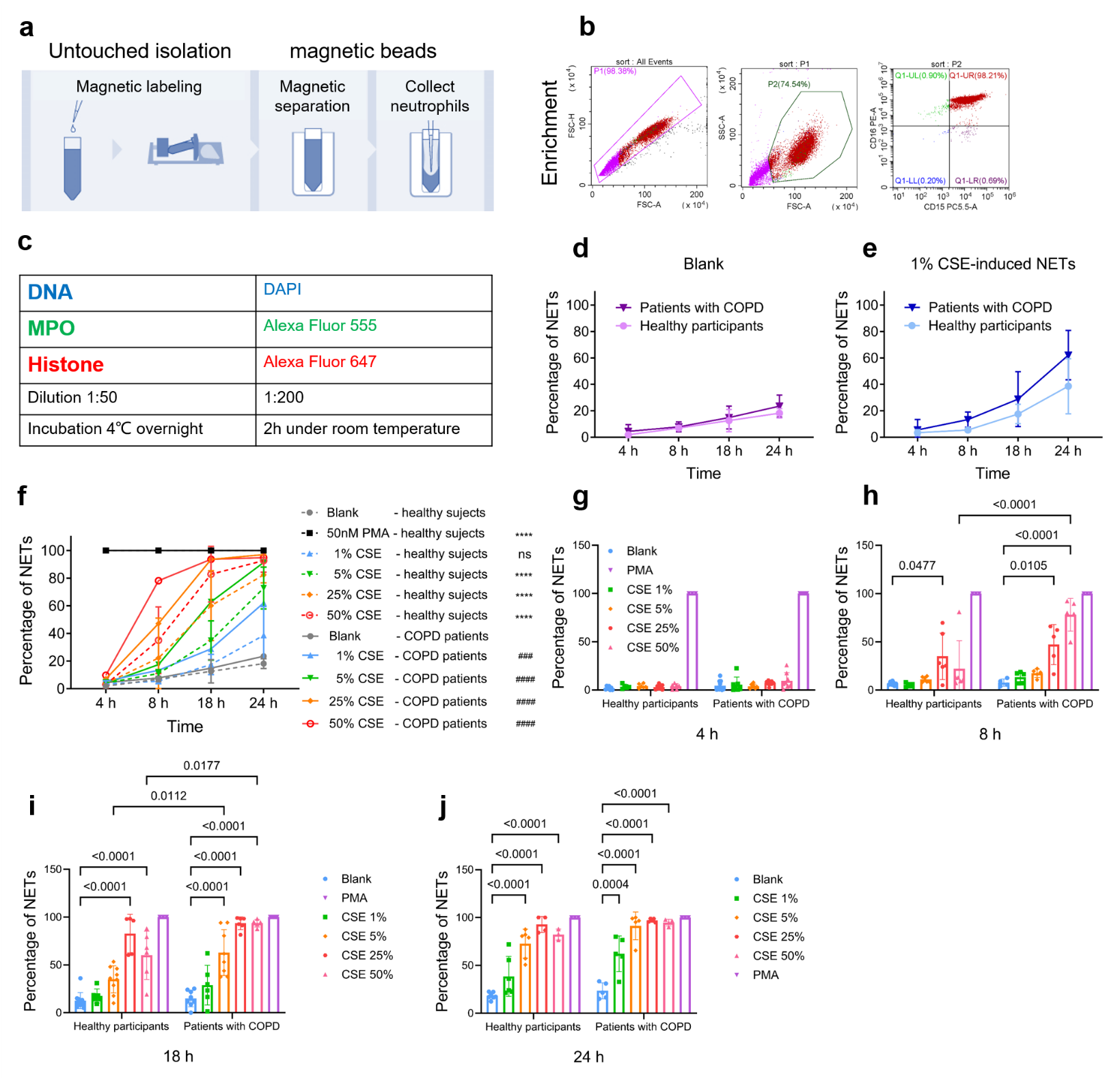


**Figure S2.** Isolation of human peripheral blood neutrophils, identification and quantification of neutrophil extracellular traps (NETs) by immunofluorescence staining of NETs components. ***Statistical analysis***: ***n = 3 – 11*** for each point or bar in (d-j) from ***3 - 11*** healthy participants and ***3 - 10*** patients with COPD, respectively, data are presented as mean±standard deviation; Differences with p value are tested by (d-f) two-way and (g-j) one-way ANOVA analysis of variance followed Tukey’s honestly significant difference analysis; **** P<0.05, *** P<0.001, **** P<0.0001, ^#^ P<0.05, ^###^ P<0.001,*** and ***^####^ P<0.0001*** represent significant difference from the group of blank, the scattered samples and the p values are displayed in (g-j). **(a)** Schematic diagram of magnetic beads-based untouched isolation of human peripheral blood neutrophils (hPBNs, Method 4, image derived from [www.miltenyibiotec.com](http://www.miltenyibiotec.com/)). **(b)** Enrichment of human peripheral neutrophils using the method described in (a), as assessed by flow cytometry (Method 17). **(c)** Immunofluorescence co-staining strategy to identify and quantify NETs (Method 7). **(d, e)** Quantified percentage of (d) spontaneous NETosis and (e) NETosis induced by 1% CSE (Method 7). **(f)** Combined percentage of NETosis induced by increasing doses of CSE in neutrophils derived from both patients with COPD and healthy participants (Method 7). **(g-j)** Quantified percentage of NETosis induced by increasing doses of CSE at (g) 4 h, (h) 8 h, (i) 18 h and (j) 24 h (Method 7).


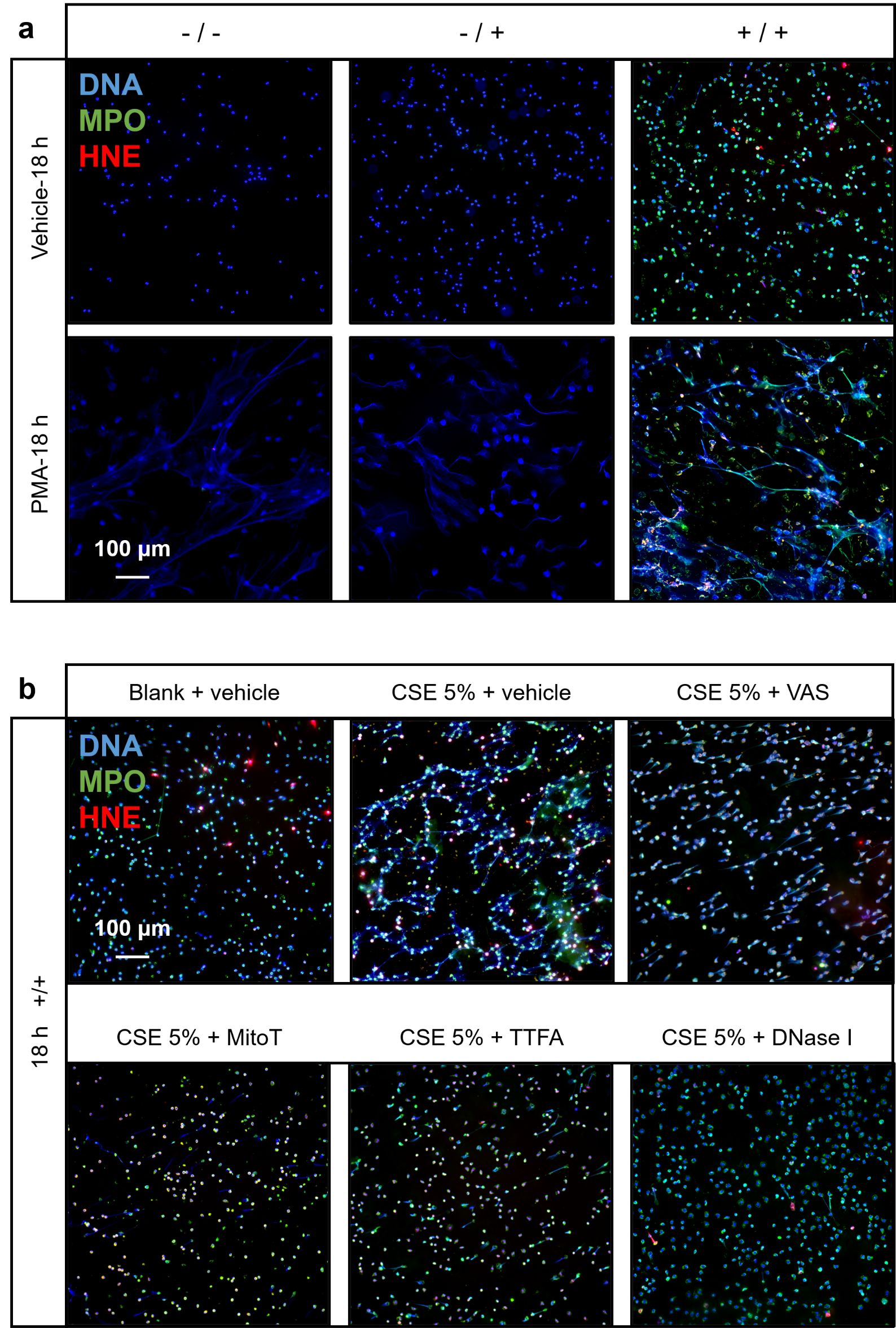


**Figure S3.** Representative immunofluorescence co-staining images of neutrophil extracellular traps (NETs) induced by phorbol-12-myristate-13-acetate (PMA) and cigarette smoke extract (CSE) (scale bar: 100 μm). **(a)** NETs were labeled by immunofluorescence antibodies against myeloperoxidase (MPO, green) and histone H3 (red), DNA was stained by DAPI, as described in Method 7; “+/+” represents staining with primary and second antibodies, “-/+” represents nonspecific control stained with only second antibodies, “-/-” represents no staining of primary and second antibodies. **(b)** Representative immunofluorescence co-staining images of NETosis induced by 5% CSE with or without treatment of VAS2870 (VAS, a NADPH oxidase inhibitor), mitoTEMPO (MitoT, a mitochondrially targeted antioxidant), thenoyltrifluoroacetone (TTFA, a mitochondrial respiration inhibitor) and deoxyribonuclease-I (DNase-I, an endonuclease for single- and double-stranded DNA).


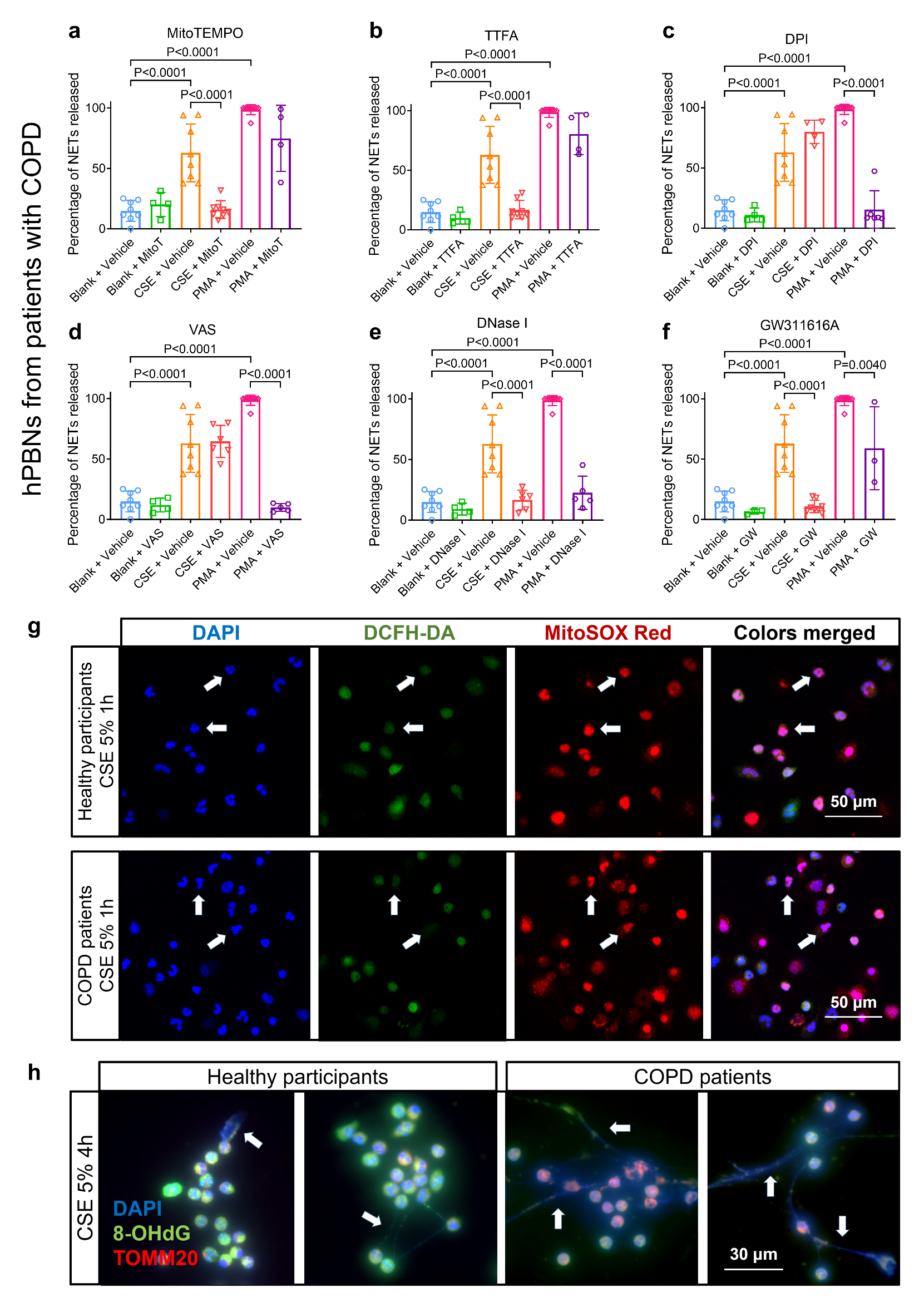


**Figure S4.** Additional evidences to support that cigarette smoke extract (CSE)-induced NETosis requires mitochondrial reactive oxygen species (ROS) and mitochondrial respiratory chain, but not nicotinamide adenine dinucleotide phosphate (NADPH) oxidase. ***Statistical analysis***: ***n = 3 - 11*** for each bar in (a-f) from ***3-11*** healthy participants or ***3-10*** patients with COPD in, data are presented as mean ± standard deviation; Differences with p value are tested by (a-f) one-way ANOVA analysis of variance followed Tukey’s honestly significant difference analysis; ***P < 0.05*** represents significant difference, the scattered samples and the p values are displayed in figures. **(a-f)** Effects of different chemicals on NETosis of human peripheral blood neutrophils (hPBNs, derived from patients with COPD) treated with CSE 5 % or PMA 50 nM for 18 h: (a) mitoTEMPO 50 μM, a mitochondrially targeted antioxidant), (b) thenoyltrifluoroacetone (TTFA) 50 μM, a mitochondrial respiration inhibitor, (c) diphenyleneiodonium chloride (DPI) 50 μM, a NADPH oxidase inhibitor, (d) VAS2870 (VAS) 50 μM, a NADPH oxidase inhibitor, (e) deoxyribonuclease I (DNase-I) 200 IU/mL, an endonuclease for single- and double-stranded DNA, and (f) GW311616A 50 μM, a selective human neutrophil elastase inhibitor. The percentage of NETosis was characterized by immunofluorescence co-staining of NETs components (Method 7). **(g)** Representative fluorescence images display MitoSOX-stained mitochondrial ROS (red) and Dichlorodihydrofluorescein diacetate (DCFH-DA) stained cellular ROS (green) in hPBNs (derived from either healthy participants or patients with COPD) treated with CSE 5% for 1h (Method 8), white arrows and merged results (purple) indicate the majority of cellular ROS originated from mitochondria. **(h)** Representative fluorescence images display colocalization of DNA and 8-OHdZG on NETs (derived from either healthy participants or patients with COPD) induced by CSE 5% (indicated by white arrow, scale bar: 10 μm), suggesting oxidatively damaged DNA in NETs (Method 8)*.*


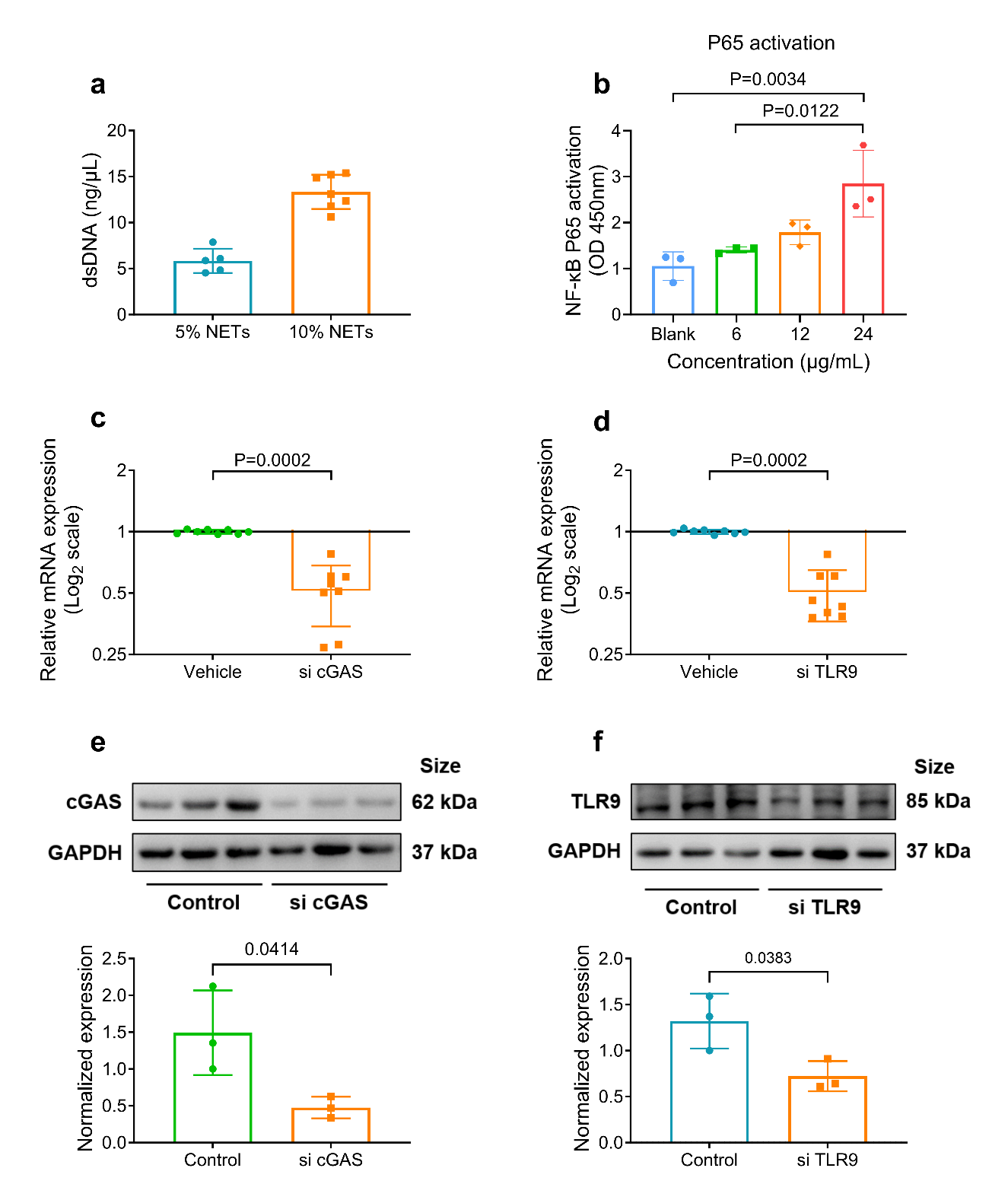


**Figure S5. (a)** Quantification of DNA concentration in NETs-containing supernatants by using fluorescence quantitative assay (Method 9); **(b)** Primary hAECs treated with increasing concentrations of NETs showed increased NF-κB P65 activations as assessed by the level of DNA-binding for activated P65 (Method 27); **(c, d)** Quantification of (c) *cGAS* and (d) *TLR9* mRNA expressions in human airway epithelial cells (hAECs) with or without silence of *cGAS* and *TLR9*, respectively (Method 12, 13); **(e, f)** Quantification of (e) cGAS and (f) TLR9 protein expressions in hAECs with or without silence of *cGAS* and *TLR9*. ***Statistical analysis***: ***n = 5 - 7*** in (a) from ***5*** healthy participants; ***n = 8 - 10*** for each bar in (c) and (d), ***n = 3*** for each bar in (b), (e) and (f) from 3 independent experiments, data are presented as mean ± standard deviation; Differences with p value are tested by (a, c-f) unpaired t-test or Mann-Whitney test according to the normal distribution examined by the Shapiro-Wilk normality test, and (b) one-way ANOVA analysis of variance followed Tukey’s honestly significant difference analysis. ***P < 0.05*** represents significant difference, the scattered samples and the p values are displayed in figures.


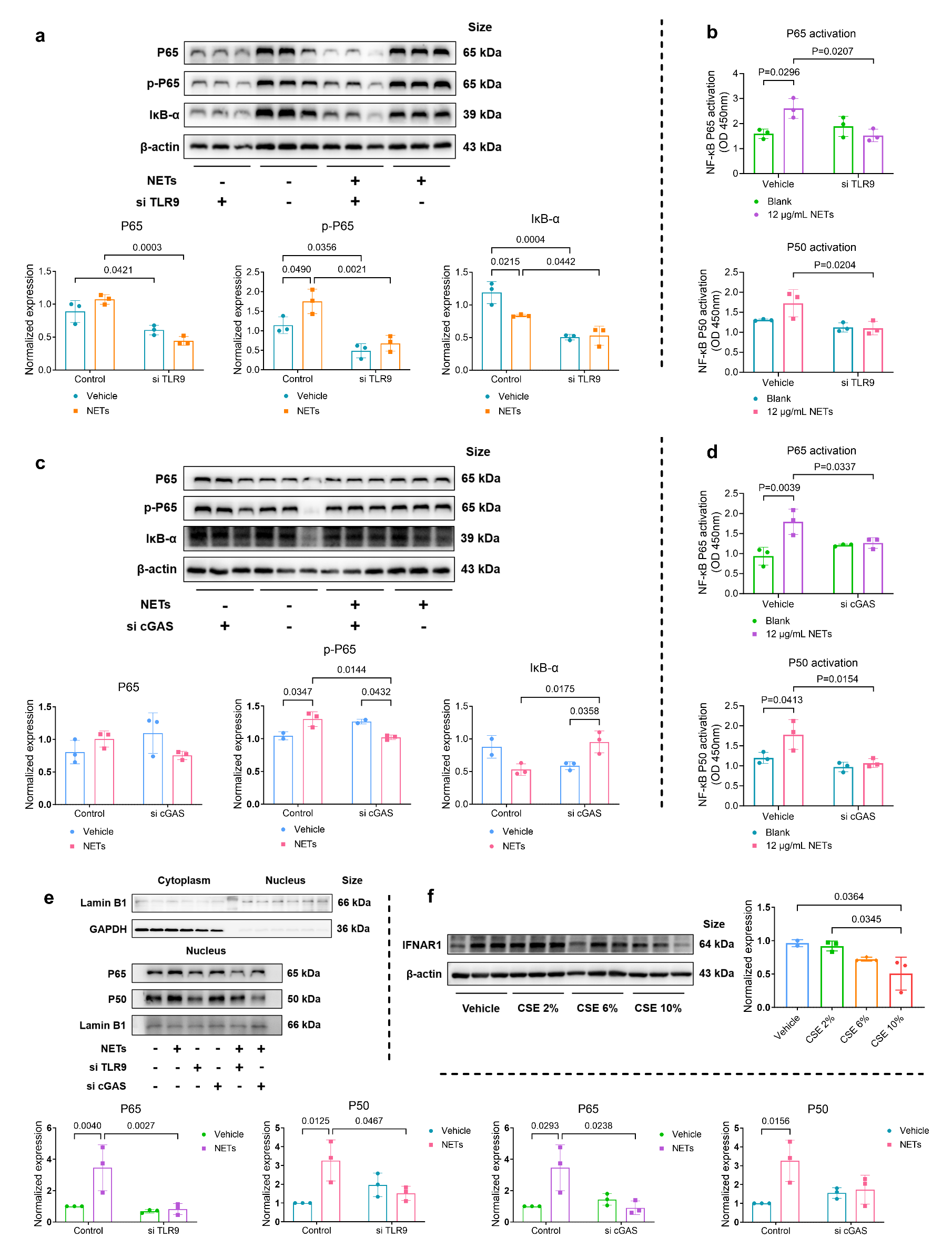


**Figure S6.** Treatment of 12 μg / mL NETs on human airway epithelial cells (hAECs) for 48 h activates nuclear factor kappa B (NF-κB) signaling pathway, which is inhibited by the silence of TLR9 and cGAS respectively (Method 11, 12); Treatment of increasing dose of CSE on hAECs for 48 h results in decreased protein-expression of IFN-α/β receptor Subunit-1 (IFNAR1). ***Statistical analysis***: ***n = 3*** for each bar in (a-f) from at least ***3*** independent experiments, data are presented as mean ± standard deviation; Differences with p value are tested by (a-e) two-way and (f) one-way ANOVA analysis of variance followed Tukey’s honestly significant difference analysis; ***P < 0.05*** represents significant difference, the scattered samples and the p values are displayed in figures. **(a, c)** Relative protein levels of P65, phosphorylated P65 (p-P65) and IκB-α are assessed by western blot and quantified by densitometry (Method 14); The activation of NF-κB signaling is suggested by the increased level of p-P65 and decreased level of IκB-α, as normalized to the level of β-actin. The activation of NF-κB is inhibited by the silence of TLR9 and cGAS respectively. **(b, d**) NF-κB P65 and P50 activations are assessed by the level of DNA-binding for activated P65 and P50 (Method 27). NETs-DNA induce activation of NF-κB P65 and P50, which are inhibited by the silence of TLR9 and cGAS respectively. **(e)** Protein levels of P65 and P50 in nucleus of hAECs are assessed by western blot and quantified by densitometry (Method 14); The nuclear extracts of hAECs were separated and confirmed by the nucleus marker Lamin B1 and the cytoplasm marker GAPDH; The activation of NF-κB signaling is suggested by the increased protein levels of P65 and P50 in nucleus of hAECs. **(f)** The relative protein level of IFNAR1 (normalized to the level of β-actin) is assessed by western blot and quantified by densitometry (Method 14).


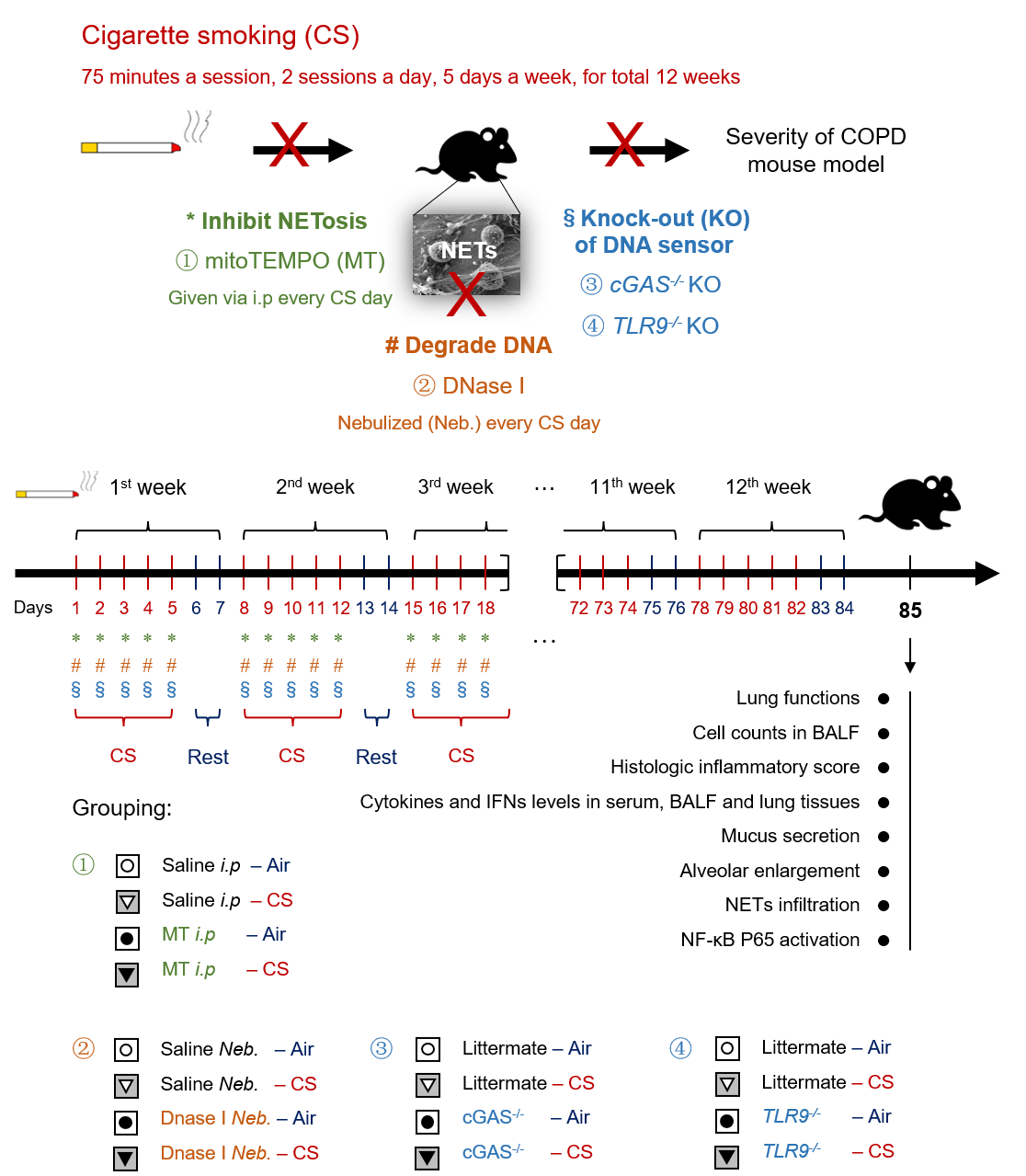


**Figure S7.** An outline of experimental design for the COPD mouse model. A well-established COPD mouse model was obtained by an acknowledged nose-only CS exposure method, this mouse model presents clearly airflow limitation- and emphysema-phenotype similar to that induced by whole-body CS exposure for 6 months: briefly, the mice were subjected to 75 min exposure of smoke generated from ~30 cigarettes for each session, 2 sessions a day separated by a recovery period (~2 h), 5 days a week, for total 12 weeks. The control mice were exposed to fresh air using the same protocol. The pathological manifestations and the detailed apparatus and methods used for the establishment of this mouse model has been described previously (Method 19). cGAS knockout (*cGAS^-/-^* KO) and TLR9 knockout (*TLR9^-/-^* KO) mice were obtained from Jackson Labs, the knockout efficacy of target gene in these strains have been widely verified (Method 18). The treatment of mitoTEMPO via intraperitoneal injection (*i.p*), DNase-I via nebulization (*Neb.*), were given respectively on the days of cigarette smoking (Method 20). The mice were scarified on day 85 and further evaluated for the severity of airway inflammation, airflow limitation, emphysema, neutrophil extracellular traps (NETs) infiltration and production of NF-кB-dependent cytokines and type-I interferons in serum, bronchoalveolar lavage fluid (BALF) and lung tissues of mice (Method 21, 22, 23, 24, 25 and 26).


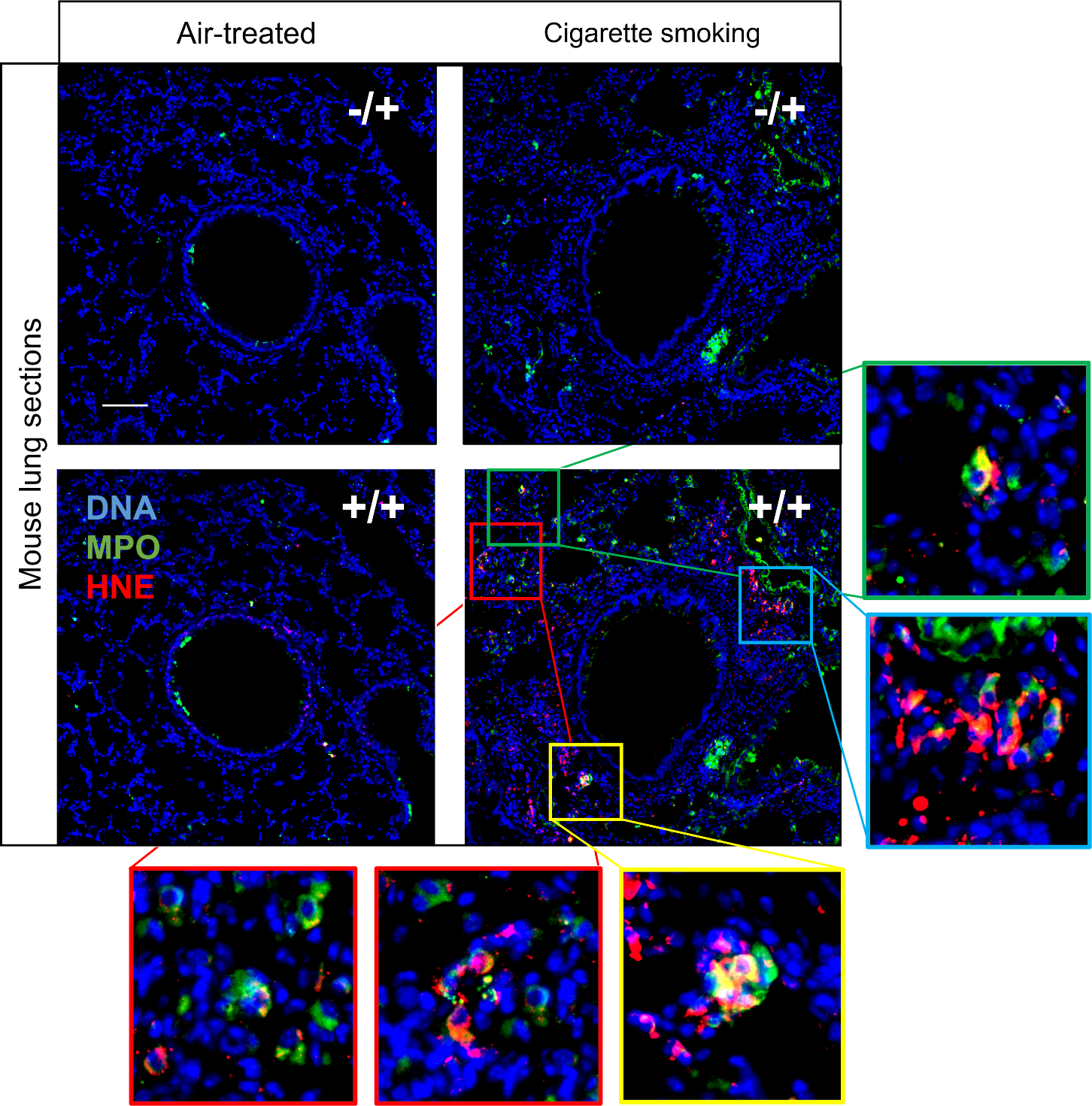


**Figure S8.** Representative immunofluorescence co-staining images of neutrophil extracellular traps (NETs) in lung slices of mice (scale bar: 100 μm). NETs were labeled by immunofluorescence antibodies against myeloperoxidase (MPO, green) and histone H3 (red), DNA was stained by DAPI. The detailed immunofluorescence co-staining and quantifications of NETs in lung slices of mice were described in Figure S2c and Method 24. +/+ represents staining with primary and second antibody, -/+ represents nonspecific control stained with only second antibody. The amplified colored panels display typical co-stained images of NETs in lung slices of mice.


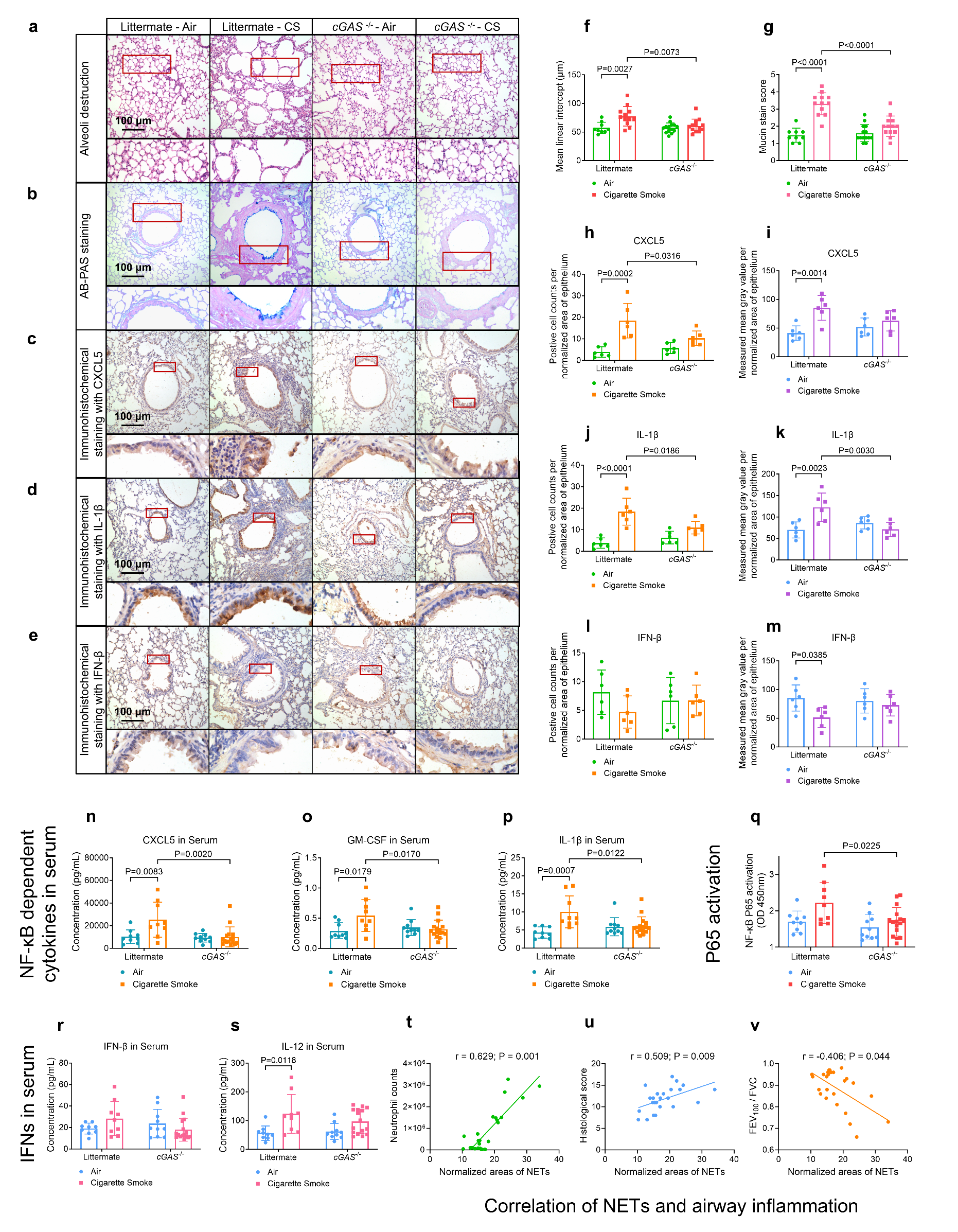


**Figure S9.** cGAS knock out (*cGAS^-/-^*) mice treated with cigarette smoke (CS) exposure display reduced mucus secretion, alveolar enlargement, production of nuclear factor kappa B (NF-κB)-dependent cytokines in serum and lung tissues, NF-κB P65 activation in lung tissues, compared to CS-treated littermate, while no significant change of type-I interferons (IFNs) in serum and lung tissues are observed; The severity of NETs infiltration is correlated with the severity of neutrophil inflammation, the histological pathology and the airflow-limitation in COPD mouse model. ***Statistical analysis***: ***n = 9 – 20*** for each bar in (f, g) and (n-s), ***n = 6*** for each bar in (h-m), ***n = 25*** in (t-v), data are presented as mean ± standard deviation; Differences with p value are tested by (f-s) two-way ANOVA analysis of variance followed Tukey’s honestly significant difference analysis; The correlation analysis (t-v) with p value is performed by using Pearson’s correlation analysis, followed by multiple linear regression analysis; ***P < 0.05*** represents significant difference, the scattered samples and the p values are displayed in figures. **(a-m)** Representative images of (a) hematoxylin-eosin (H&E)-stained lung slices, (b) Alcian Blue-Periodic Acid Schiff (AB-PAS)-stained lung slices, immunohistochemical staining for (c) CXCL5 (C-X-C motif chemokine ligand 5), (d) IL-1β (interleukin 1β) and (e) IFN-β in lung slices, display that the severity of (f) alveolar enlargement (mean linear intercept) and (g) mucus secretion (Method 23), the levels of (h, i) CXCL5 and (j, k) IL-1β (evaluated by positive cell counts and measured mean gray value per normalized area of airway epithelium, Method 25) decreases in CS-treated *cGAS^-/-^* mice compared to CS-treated littermate (scale bar: 100 μm), while no significant change of (l, m) IFN-β in lung slice of either CS-treated littermate or CS-treated cGAS-/- mice is observed. **(n-p)** CS-treated *cGAS^-/-^* mice display overall reduced production of NF-κB dependent inflammatory cytokines in serum: (n) CXCL5, (o) GM-CSF (granulocyte macrophage colony-stimulating factor) and (p) IL-1β. **(r, s)** No significant change of type-I IFNs in serum of either CS-treated littermate or CS-treated *cGAS^-/-^* mice is observed: (r) IFN-β, (s) IL-12 (Method 22, 26). **(q)** CS exposure induces increased NF-κB P65 activation as assessed by DNA-binding activity of P65 in lung tissue of mice, while CS-treated *cGAS^-/-^* mice display reduced P65 activation (Method 27). **(t-v)** The severity of NETs infiltration (evaluated by the normalized area of NETs in immunofluorescence antibodies stained lung slices) is correlated with (t) neutrophil counts in BALF, (u) histological score (evaluated based on the histological changes in H&E stained lung slices), and negatively correlated with (v) severity of airflow limitation (evaluated by the forced expiratory volume at 100 ms / forced vital capacity, FEV_100_ / FVC) in CS-treated *cGAS^-/-^* mice and CS-treated littermate (Method 21, 22, 23, 24, 26).


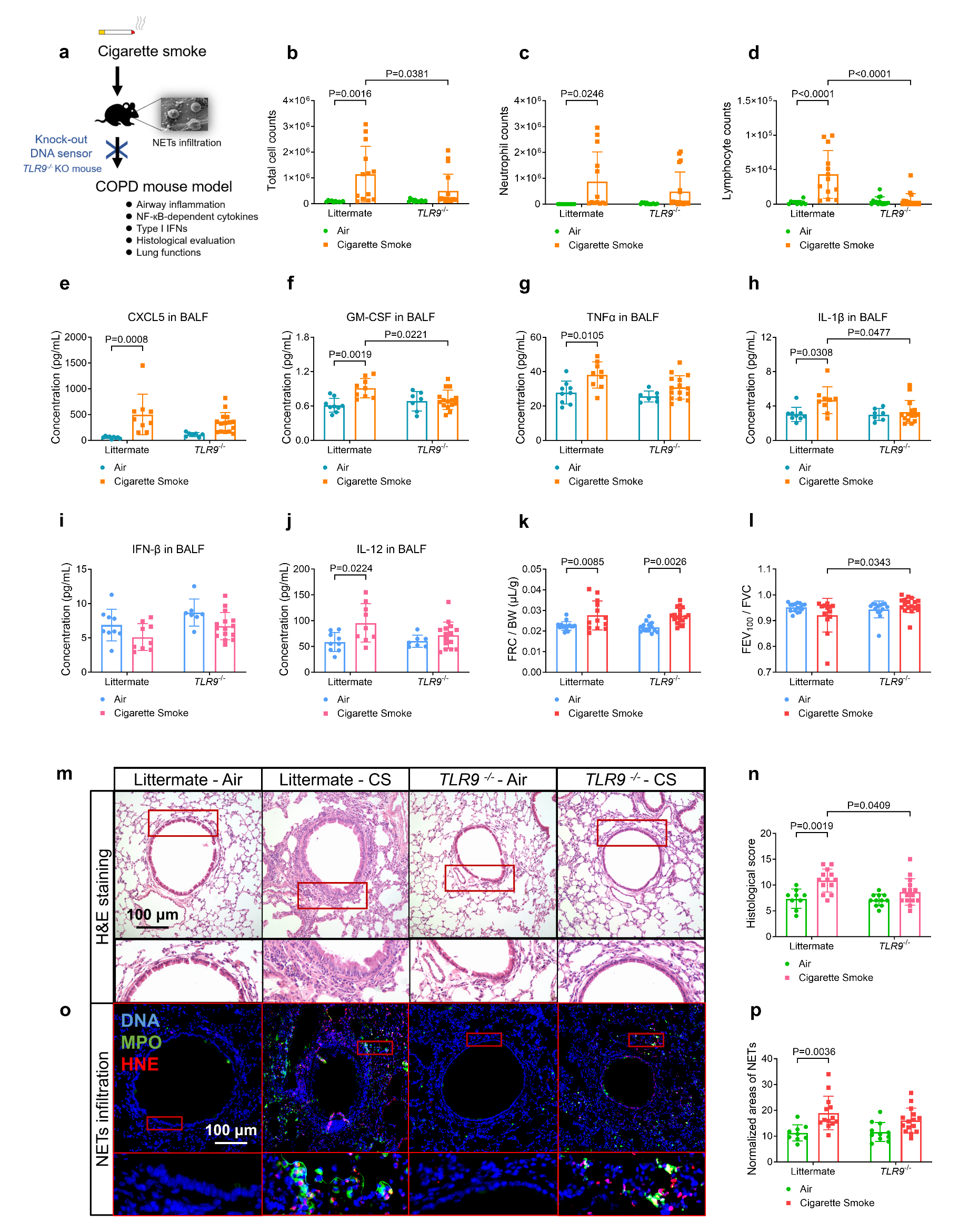


**Figure S10.** TLR9 knock out (*TLR9^-/-^*) mice treated with cigarette smoke (CS) exposure display decreased airway inflammation, production of nuclear factor kappa B (NF-κB)-dependent inflammatory cytokines, improved airflow limitation compared to CS-treated littermate. ***Statistical analysis***: ***n = 7 - 18*** mice for each bar in (b-l, n, p), data are presented as mean ± standard deviation; Differences with p value were tested by (b-l, n, p) two-way ANOVA analysis of variance followed Tukey’s honestly significant difference analysis; ***P < 0.05*** represents significant difference, the scattered samples and the p values are displayed in figures. **(a)** A brief outline for the experiments of COPD mouse model (Method 18, 19). **(b-d)** CS-treated *TLR9^-/-^* mice display overall alleviated airway inflammation as reflected by: (b) total cell counts, (c) neutrophil counts, (d) lymphocyte counts in bronchoalveolar lavage fluid (BALF, Method 22). **(e-h)** CS-treated *TLR9^-/-^* mice display overall reduced production of NF-κB dependent inflammatory cytokines in BALF (Method 22, 26): (e) CXCL5 (C-X-C motif chemokine ligand 5), (f) GM-CSF (granulocyte macrophage colony-stimulating factor), (g) TNFα (tumor necrosis factor alpha), (h) IL-1β (interleukin 1β). **(i, j)** No significant change of type-I interferons (IFNs) in BALF of *TLR9^-/-^* mice is observed: (i) IFN-β, (j) IL-12. **(k, l)** CS-treated *TLR9^-/-^* mice display alleviated airflow limitation, but not emphysema, in lung function test (Method 21) as evaluated by: (k) FRC / BW (functional residual capacity / body weight), (l) FEV_100_ / FVC (forced expiratory volume at 100 ms / forced vital capacity). **(m)** Representative images of hematoxylin-eosin (H&E)-stained lung slices displayed the decreased severity of airway inflammation in CS-treated *TLR9^-/-^* mice compared to CS-treated littermate (scale bar: 100 μm, Method 23), as summarized in **(n)** histological score. **(o)** Representative immunofluorescence images display no significant change of NETs (co-stained with DNA, MPO and Histone H3) infiltration in CS-treated *TLR9^-/-^* mice (scale bar: 100 μm, Method 24), as summarized in **(p)** normalized area of NETs.


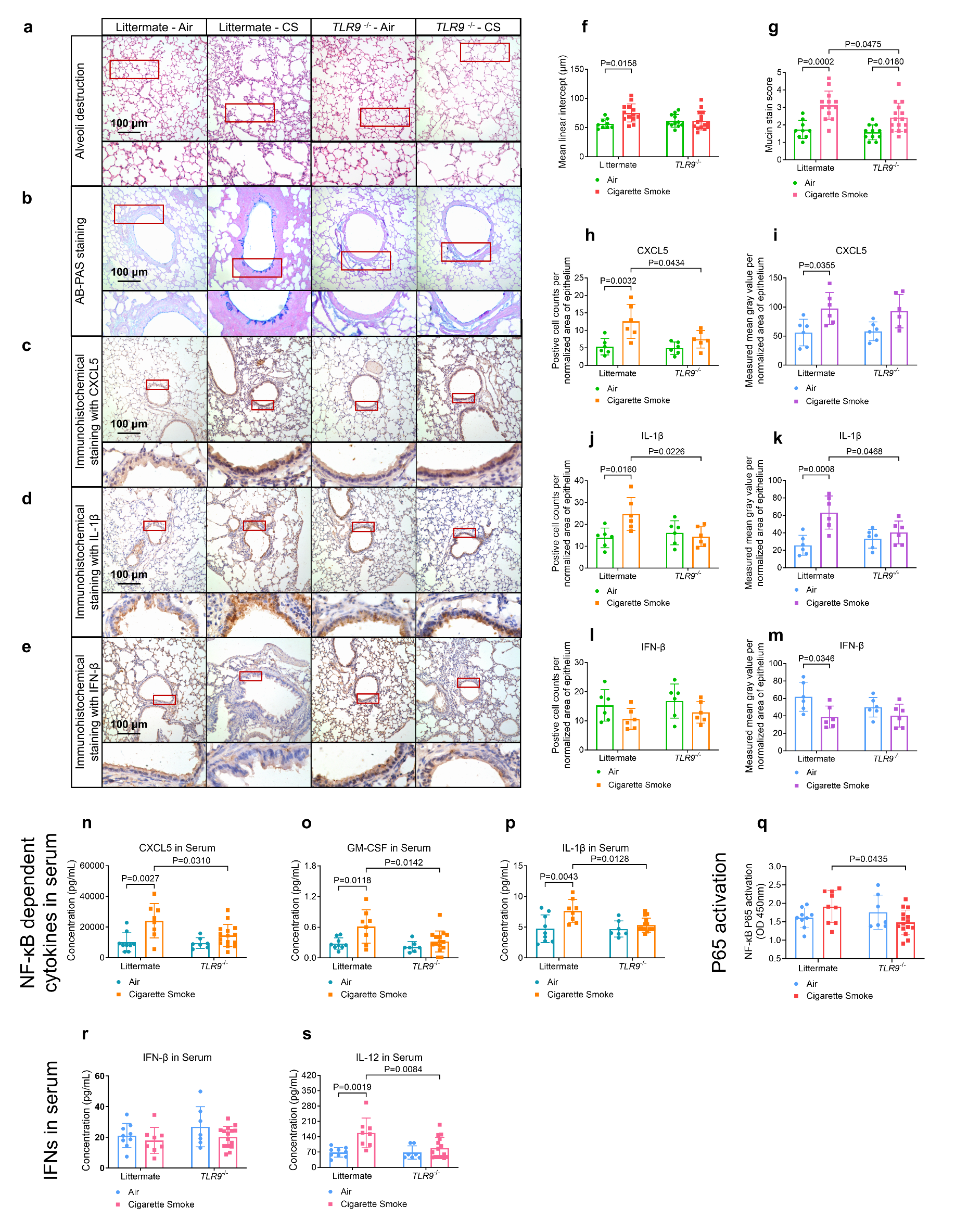


**Figure S11.** TLR9 knock out (*TLR9^-/-^*) mice treated with cigarette smoke (CS) exposure display reduced mucus secretion, alveolar enlargement, production of nuclear factor kappa B (NF-κB)-dependent cytokines in serum and lung tissues, NF-κB P65 activation in lung tissues, compared to CS-treated littermate. ***Statistical analysis***: ***n = 7 – 18*** for each bar in (f, g) and (n-s), ***n = 6*** for each bar in (h-m), data are presented as mean ± standard deviation; Differences with p value are tested by (f-s) two-way ANOVA analysis of variance followed Tukey’s honestly significant difference analysis; ***P < 0.05*** represents significant difference, the scattered samples and the p values are displayed in figures. **(a-m)** Representative images of (a) hematoxylin-eosin (H&E)-stained lung slices, (b) Alcian Blue-Periodic Acid Schiff (AB-PAS)-stained lung slices, immunohistochemical staining for (c) CXCL5 (C-X-C motif chemokine ligand 5), (d) IL-1β (interleukin 1β) and (e) IFN-β (interferons-β in lung slices, display that the severity of (g) mucus secretion (Method 23), the levels of (h, i) CXCL5 and (j, k) IL-1β (evaluated by positive cell counts and measured mean gray value per normalized area of airway epithelium, Method 25) decreases in CS-treated *TLR9^-/-^* mice compared to CS-treated littermate (scale bar: 100 μm), while no significant change of (f) alveolar enlargement (mean linear intercept) and (l, m) IFN-β in lung slice of either CS-treated littermate or CS-treated *TLR9^-/-^* mice is observed. **(n-p)** CS-treated *TLR9^-/-^* mice display overall reduced production of NF-κB dependent inflammatory cytokines in serum: (n) CXCL5, (o) GM-CSF (granulocyte macrophage colony-stimulating factor) and (p) IL-1β. **(r, s)** CS-treated *TLR9^-/-^* mice display reduced level of (s) IL-12, but not (r) IFN-β in serum, compared to CS-treated littermate (Method 22, 26). **(q)** CS exposure induces increased NF-κB P65 activation as assessed by DNA-binding activity of P65 in lung tissue of mice, while CS-treated *TLR9^-/-^* mice display reduced P65 activation (Method 27).


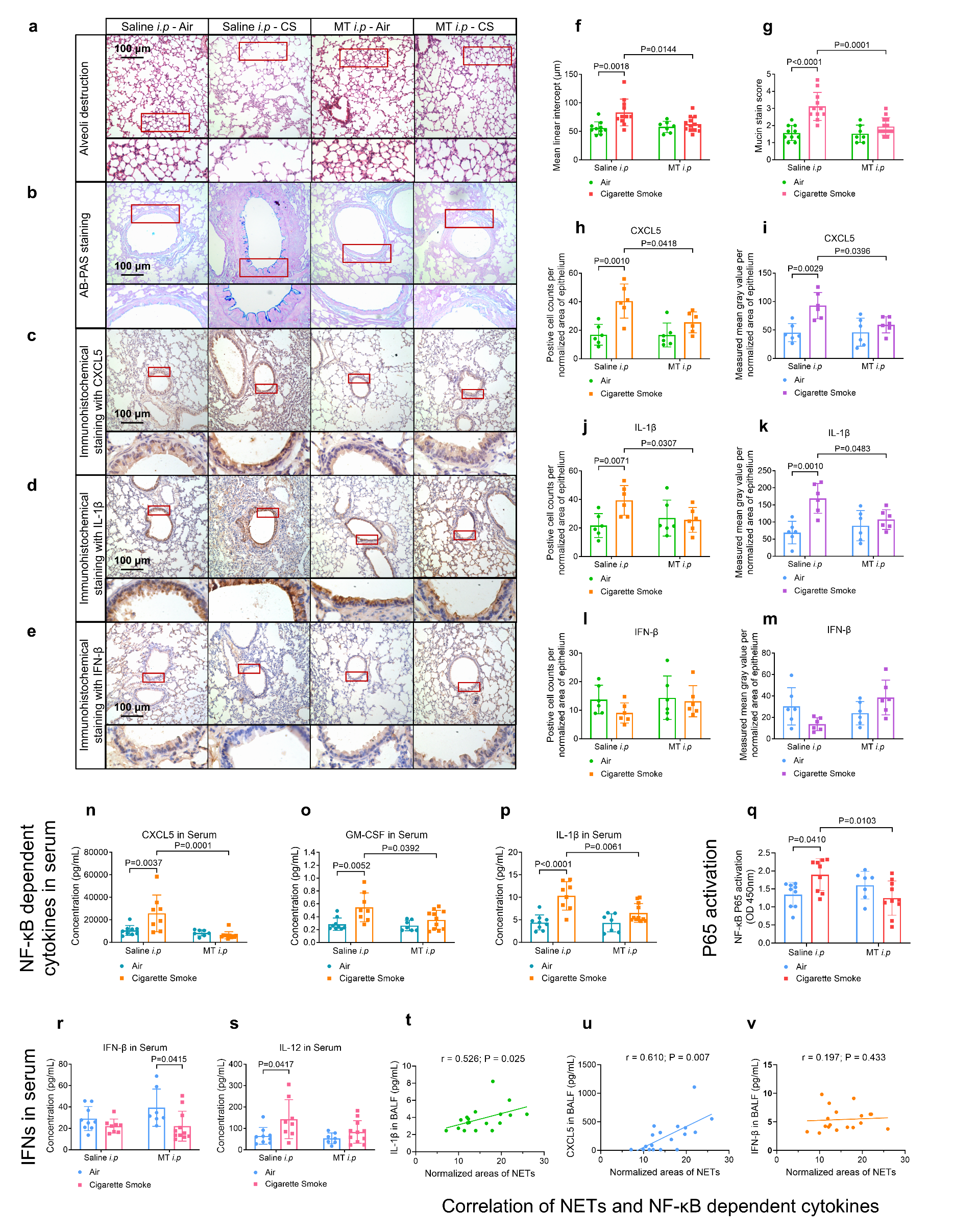


**Figure S12.** Cigarette smoke (CS)-exposed wild type mice treated with intraperitoneal injection (*i.p*) of mitoTEMPO (MT) display reduced mucus secretion, alveolar enlargement, production of nuclear factor kappa B (NF-κB)-dependent cytokines in serum and lung tissues, NF-κB P65 activation in lung tissues, compared to CS-treated saline *i.p* mice, while no significant change of type-I interferons (IFNs) in serum and lung tissues are observed; The severity of NETs infiltration is correlated with the levels of NF-κB dependent inflammatory cytokines, but not correlated with the levels of type-I interferons (IFNs, specifically IFN-β) in the BALF of CS-treated mice. ***Statistical analysis***: ***n = 7 – 16*** for each bar in (f, g) and (n-s), ***n = 6*** for each bar in (h-m), ***n = 18*** in (t-v), data are presented as mean ± standard deviation; Differences with p value are tested by (f-s) two-way ANOVA analysis of variance followed Tukey’s honestly significant difference analysis; The correlation analysis (t-v) with p value is performed by using Pearson’s correlation analysis, followed by multiple linear regression analysis; ***P < 0.05*** represents significant difference, the scattered samples and the p values are displayed in figures. **(a-m)** Representative images of (a) hematoxylin-eosin (H&E)-stained lung slices, (b) Alcian Blue-Periodic Acid Schiff (AB-PAS)-stained lung slices, immunohistochemical staining for (c) CXCL5 (C-X-C motif chemokine ligand 5), (d) IL-1β (interleukin 1β) and (e) IFN-β in lung slices, display that the severity of (f) alveolar enlargement (mean linear intercept) and (g) mucus secretion (Method 23), the levels of (h, i) CXCL5 and (j, k) IL-1β (evaluated by positive cell counts and measured mean gray value per normalized area of airway epithelium, Method 25) decreases in CS-treated MT *i.p* mice compared to CS-treated saline *i.p* mice (scale bar: 100 μm), while no significant change of (l, m) IFN-β in lung slice of either CS-treated saline *i.p* mice or MT *i.p* mice is observed. **(n-p)** CS-treated MT *i.p* mice display overall reduced production of NF-κB dependent inflammatory cytokines in serum: (n) CXCL5, (o) GM-CSF (granulocyte macrophage colony-stimulating factor) and (p) IL-1β. **(r, s)** No significant change of type-I interferons (IFNs) level in serum of either CS-treated saline *i.p* mice or MT *i.p* mice is observed: (r) IFN-β, (s) IL-12 (Method 22, 26). **(q)** CS exposure induces increased NF-κB P65 activation as assessed by DNA-binding activity of P65 in lung tissue of mice, while CS-treated MT *i.p* mice display reduced P65 activation (Method 27). **(t-v)** The severity of NETs infiltration (evaluated by the normalized area of NETs in immunofluorescence antibodies-stained lung slices) is correlated with the level of (t) IL-1β and (u) CXCL5 respectively, but not correlated with the level of (v) IFN-β in the BALF of CS-treated mice (Method 22, 24, 26).


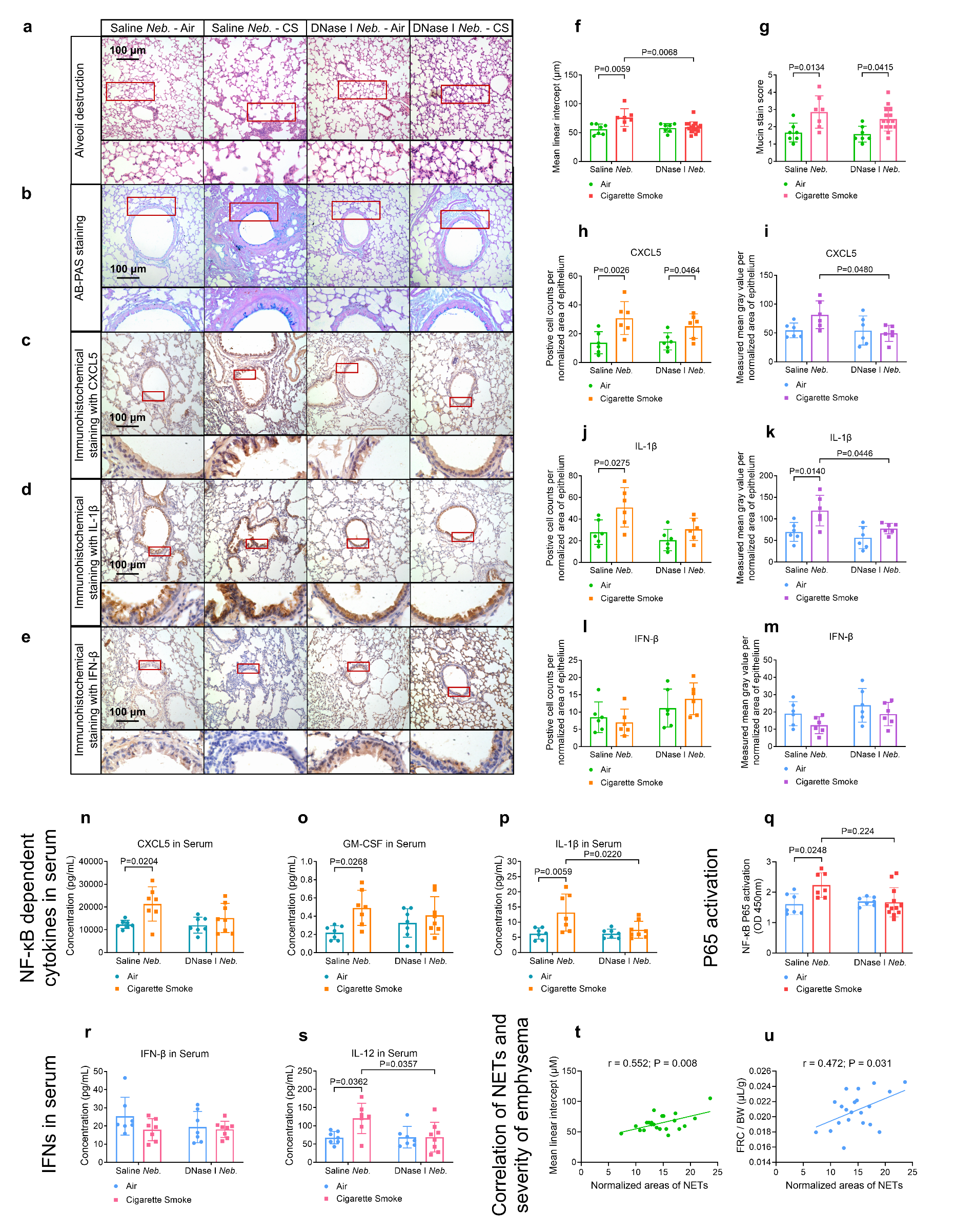


**Figure S13.** Cigarette smoke (CS)-exposed wild type mice treated with nebulization (*Neb.*) of deoxyribonuclease I (DNase-I) display reduced alveolar enlargement, production of nuclear factor kappa B (NF-κB)-dependent cytokines in serum and lung tissues, NF-κB P65 activation in lung tissues, compared to CS-treated saline *Neb.* mice. The severity of NETs infiltration is correlated with the severity of emphysema in CS-treated mice. ***Statistical analysis***: ***n = 7 - 15*** for each bar in (f, g) and (n-s), ***n = 6*** for each bar in (h-m), ***n = 22*** in (t-v), data are presented as mean ± standard deviation; Differences with p value are tested by (f-s) two-way ANOVA analysis of variance followed Tukey’s honestly significant difference analysis; The correlation analysis (t-v) with p value is performed by using Pearson’s correlation analysis, followed by multiple linear regression analysis; ***P < 0.05*** represents significant difference, the scattered samples and the p values are displayed in figures. **(a-m)** Representative images of (a) hematoxylin-eosin (H&E)-stained lung slices, (b) Alcian Blue-Periodic Acid Schiff (AB-PAS)-stained lung slices, immunohistochemical staining for (c) CXCL5 (C-X-C motif chemokine ligand 5), (d) IL-1β (interleukin 1β) and (e) IFN-β (interferons-β) in lung slices, display that the severity of (f) alveolar enlargement (mean linear intercept, Method 23), the levels of (h, i) CXCL5 and (j, k) IL-1β (evaluated by positive cell counts and measured mean gray value per normalized area of airway epithelium, Method 25) decreased in CS-treated DNase-I *Neb.* mice compared to CS-treated saline *Neb.* mice (scale bar: 100 μm), while no significant change of (g) mucus secretion and (l, m) IFN-β in lung slice of either CS-treated saline *Neb.* or CS-treated DNase-I *Neb.* mouse is observed. **(n-p)** CS-treated DNase-I *Neb.* mice display reduced production of (p) IL-1β, but not (n) CXCL5 or (o) GM-CSF (granulocyte macrophage colony-stimulating factor) in serum, compared to that of CS-treated saline *Neb.* mice. **(r, s)** CS-treated DNase-I *Neb.* mice display reduced level of (s) IL-12, but not (r) IFN-β in serum, compared to that of CS-treated saline *Neb.* mice (Method 22, 26). **(q)** CS exposure induces increased NF-κB P65 activation as assessed by DNA-binding activity of P65 in lung tissue of mice, while CS-treated DNase-I *Neb.* mice display reduced P65 activation (Method 27). **(t, u)** The severity of NETs infiltration (reflected by the normalized area of NETs in immunofluorescence-stained lung slices) is correlated with the severity of emphysema, as evaluated by (t) mean linear intercept and (u) FRC / BW (functional residual capacity / body weight) in CS-treated mice (Method 21, 22, 23, 24, 26).


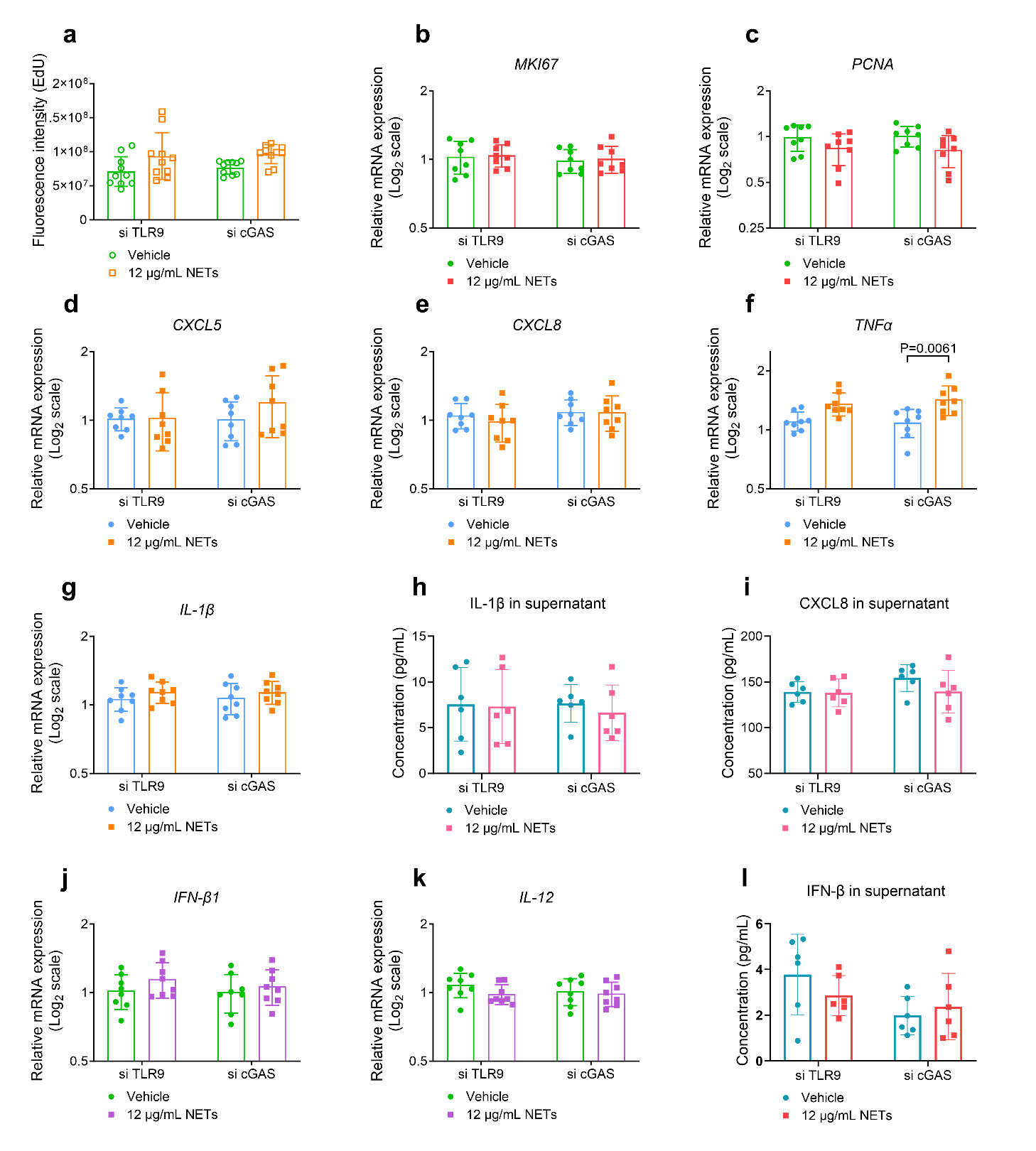


**Figure S14.** No significant differences are observed between effects of *cGAS* silence and *TLR9* silence on proliferation, productions of inflammatory cytokines and type-I interferons (IFNs) of human airway epithelial cells (hAECs) treated with 12 μg / mL NETs. ***Statistical analysis: n= 6 - 10*** for each bar in (a-l) from at least three independent experiments, data are presented as the mean ± standard deviation; Differences are assessed by the (a-l) two-way ANOVA analysis of variance, followed Tukey’s honest significant test; P<0.05 represents a significant difference, the scattered samples and the p values are displayed in figures. **(a-c)** Effects of *cGAS* and *TLR9* silencing on 12 μg/mL NETs-induced proliferation of hAECs, as assessed by the (a) EdU proliferation assay (Method 11), and the mRNA expression of (b) MKI67 and (c) PCNA (both are markers of proliferation, Method 13). **(d-l)** Effects of *cGAS* and *TLR9* silencing on 12 μg/mL NETs-induced mRNA expression and soluble levels of NF-κB-dependent inflammatory cytokines and type-I IFNs in hAECs (Method 13, 26): (d) mRNA expression of *CXCL5* (C-X-C motif chemokine ligand 5), (e) mRNA expression of *CXCL8*, (f) mRNA expression of *TNFα* (tumour necrosis factor alpha), (g) mRNA expression of *IL-1β* (interleukin 1β), (h) soluble levels of IL-1β in cell-culture supernatants, (i) soluble levels of CXCL8 in cell-culture supernatants, (j) mRNA expression of *IFN-β1*, (k) mRNA expression of *IL-12*, and (l) soluble levels of IFN-β in cell-culture supernatants.


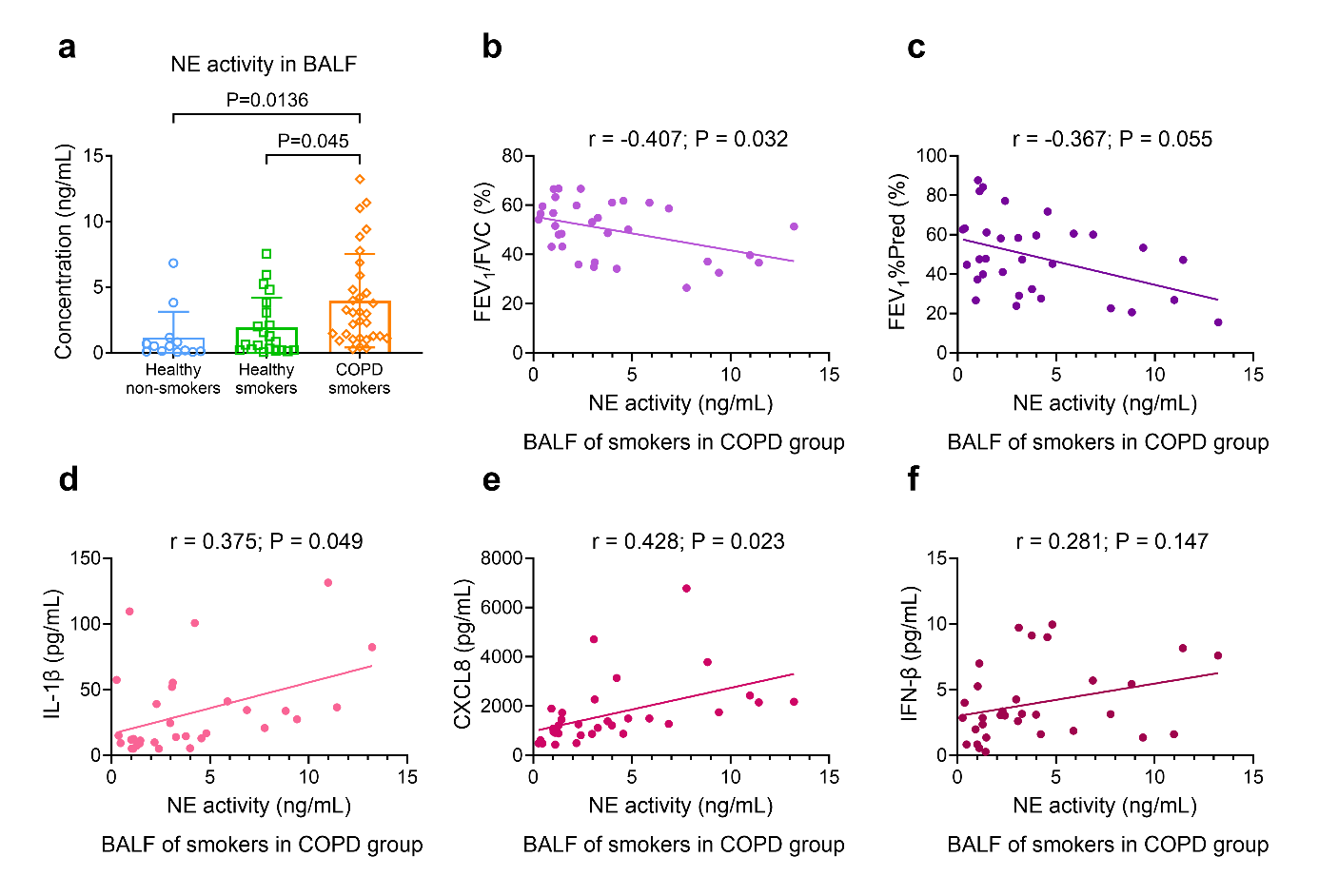


**Figure S15.** The level of neutrophil elastase (NE) activity is correlated with level of interleukin 1β (IL-1β) and C-X-C motif chemokine ligand 8 (CXCL8), but not correlated with the level of interferons-β (IFN-β) in bronchoalveolar lavage fluid (BALF) of smokers in the group of patients with COPD, after controlling for their age, sex, body mass index (BMI), and smoking history. ***Statistical analysis***: ***n = 13*** *non-smokers and* ***21*** smokers in group of healthy participants, ***n= 32*** smokers in group of patients with COPD in (a), ***n = 32*** in (b-f), data are presented as the mean ± standard deviation; Difference in (a) is assessed by one-way ANOVA analysis of variance, followed Tukey’s honest significant test; In (b-f), Pearson’s partial correlation test are performed by controlling for age, sex, BMI, and smoking history of smokers in the group of patients with COPD, followed by the multiple linear regression analysis; ***P<0.05*** represents a significant difference, the scattered samples and the p values are displayed in figures. **(a)** NE activity level is significantly increased in the BALF of smokers in group of patients with COPD, compared with that of non-smokers and smokers in healthy group, and negatively correlated with **(b)** ratio of FEV_1_ to forced vital capacity (FEV_1_/FVC), but not **(c)** ratio of forced expiratory volume at 1 s (FEV_1_) to predicted FEV_1_ (FEV_1_%Pred, Method 1, 2, 3, 26). NE activity level is correlated is correlated with level of **(d)** IL-1β and level of **(e)** CXCL8, but not level of **(f)** IFN-β.


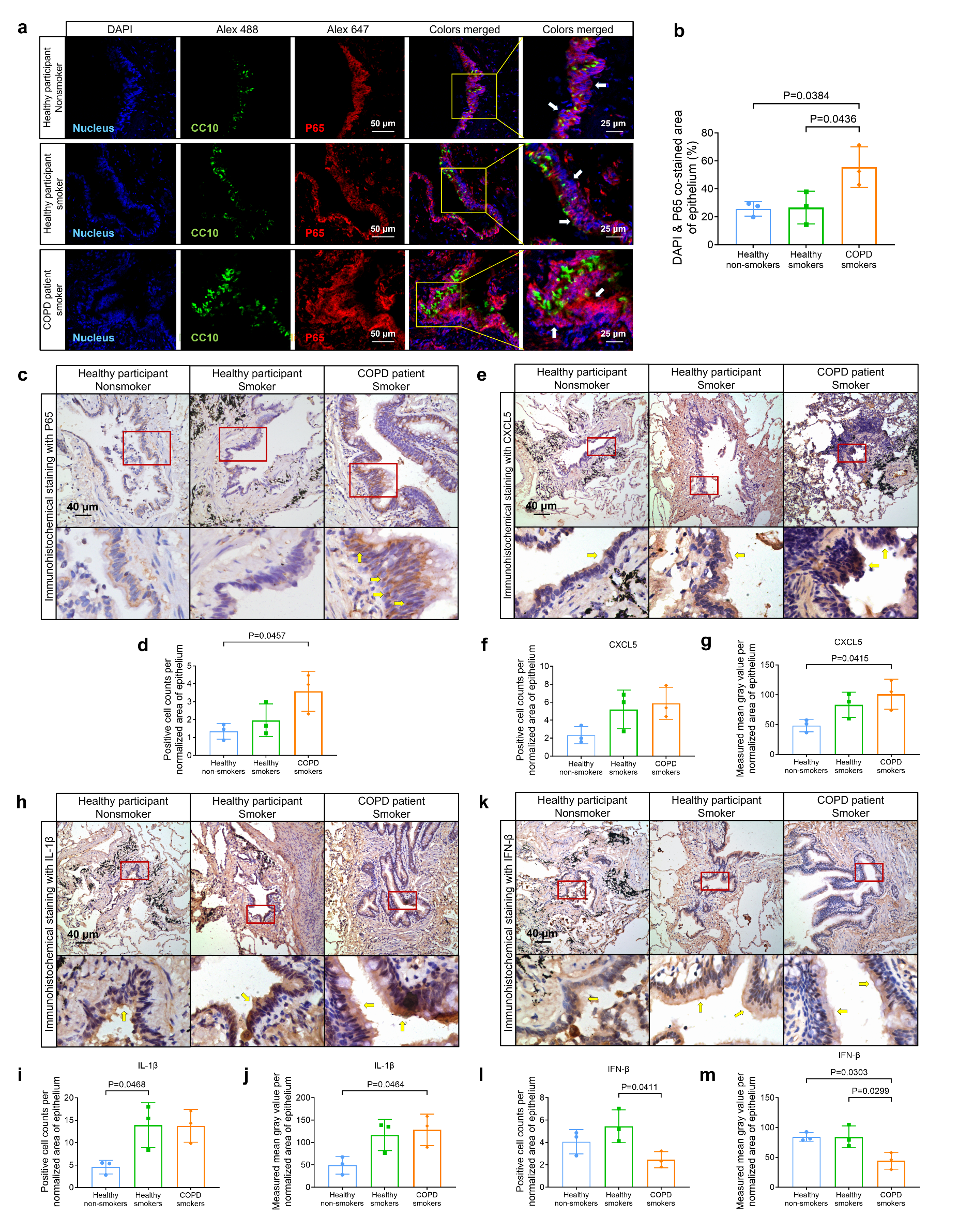


**Figure S16.** Expression of NF-κB P65 protein in nucleus of airway epithelial cells is increased in lung tissue slices of patients with COPD, compared with that of non-smokers and smokers in healthy group; Expression of NF-κB dependent inflammatory cytokines C-X-C motif chemokine ligand 5 (CXCL5) and interleukin 1β (IL-1β), but not interferons-β (IFN-β), are increased in airway epithelial cells of patients with COPD. ***Statistical analysis***: ***n = 3*** for each bar, data are presented as mean ± standard deviation; Differences with p value are tested by one-way ANOVA analysis of variance, followed Tukey’s honestly significant difference analysis; ***P < 0.05*** represents significant difference, the scattered samples and the p values are displayed in figures. **(a)** Representative immunofluorescence confocal images of airway epithelium (derived from healthy non-smoker, healthy smoker and patients with COPD) co-stained with DAPI (nucleus indicator, blue), CC10 (epithelial cell marker, green) and NF-κB P65 protein (red, Method 25). White arrows in merged images indicate increased P65 translocated into nucleus of airway epithelial cells (thus increased activation of NF-κB signaling) derived from patient with COPD, as compared with that of non-smoker and smoker in healthy group (note the separated location of P65 and nucleus), which is quantified as **(b)** co-stained area of DAPI and P65 per area of airway epithelium. **(c)** Representative immunohistochemical images of airway epithelium (derived from healthy non-smoker, healthy smoker and patients with COPD) stained with NF-κB P65 protein (brown, Method 25). Yellow arrows indicate increased staining of P65 in nucleus site of airway epithelial cells (indicating activation of NF-κB signaling) derived from patient with COPD, as compared with that of non-smoker and smoker in healthy group, which is quantified as **(d)** positive cell counts per normalized area of epithelium. **(e, h, k)** Representative immunohistochemical images of airway epithelium (derived from healthy non-smoker, healthy smoker and patients with COPD) stained with CXCL5, IL-1β and IFN-β protein, respectively (brown, Method 25). Yellow arrows indicate increased or decreased staining signals in airway epithelial cells derived from patient with COPD or smoker in healthy group, as compared with that of healthy non-smoker, which are quantified as **(f, I, l)** positive cell counts per normalized area of epithelium and **(g, j, m)** mean gray value per normalized area of airway epithelium (Method 25).


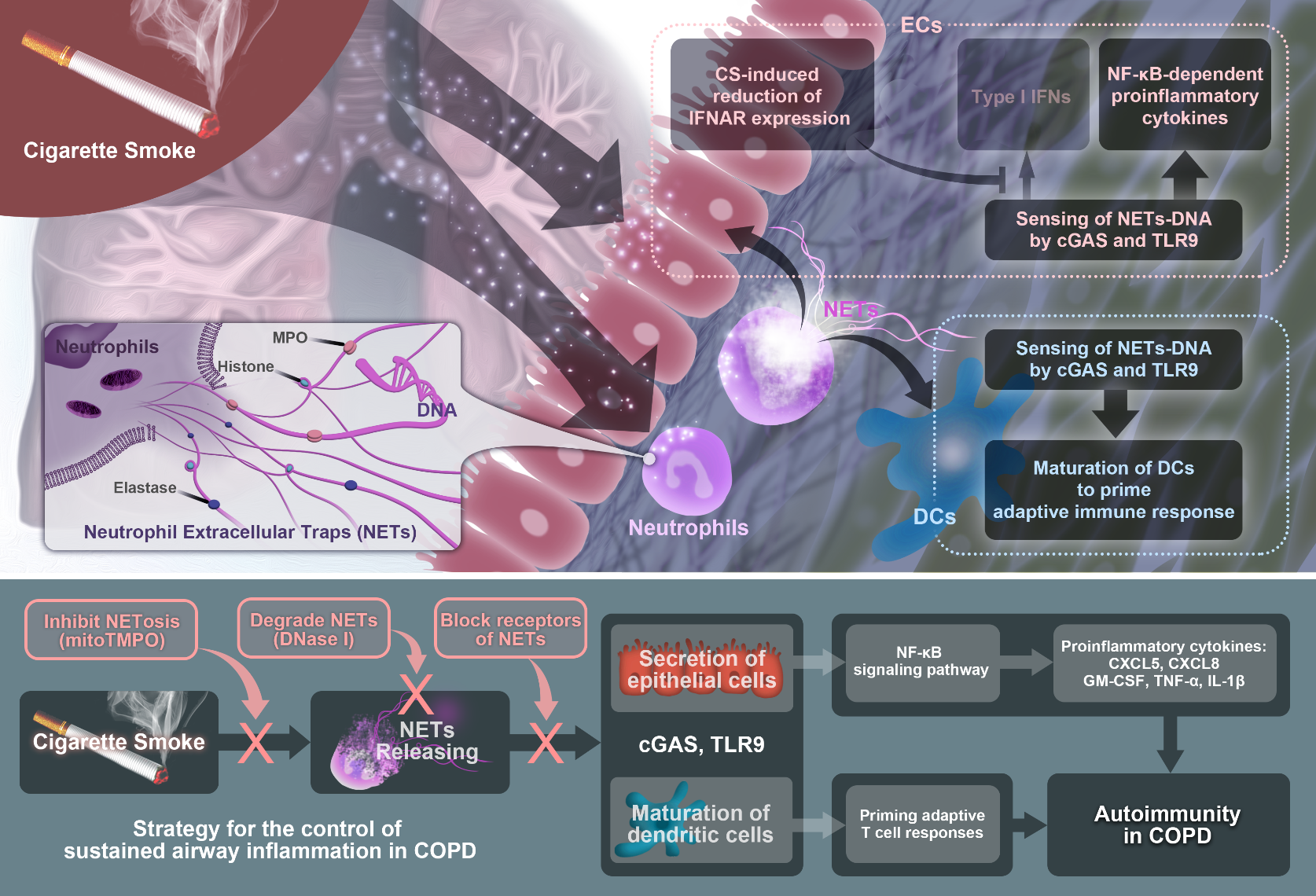


**Figure S17.** A schematic diagram displays the mechanism how neutrophil extracellular traps (NETs) contribute to the autoimmunity in the pathogenies of cigarette smoking (CS)-induced chronic obstructive pulmonary disease (COPD): CS induces neutrophils to release NETs, which contain high level of oxidatively damaged mtDNA and chDNA. The DNA components of NETs (NETs-DNA) not only stimulate the proliferation and production of nuclear factor kappa B (NF-κB)-dependent proinflammatory cytokines on airway epithelial cells (ECs), but also promote the maturation of dendritic cells (DCs) to prime adaptive response of T cells, by binding to cGAS and TLR9 (the DNA receptors on both ECs and DCs), thus contributes to the sustained airway inflammation in an autoimmune fashion in COPD. Although CS extract (CSE) stimulate type-I interferons (IFNs) production on ECs *in vitro*, the type-I IFNs productions is hampered by the long-term CS exposure *in vivo*, as CS exposure impairs the expression of IFN-α/β receptor Subunit-1 (IFNAR), a crucial receptor to facilitate the downstream signaling cascade of type-I IFNs. Abbreviations: MPO, myeloperoxidase; cGAS, cyclic guanosine monophosphate-adenosine monophosphate synthase; TLR9, toll-like receptor 9; IL-6, interleukin 6; TNFα, tumor necrosis factor alpha; CXCL5, C-X-C motif chemokine ligand 5.


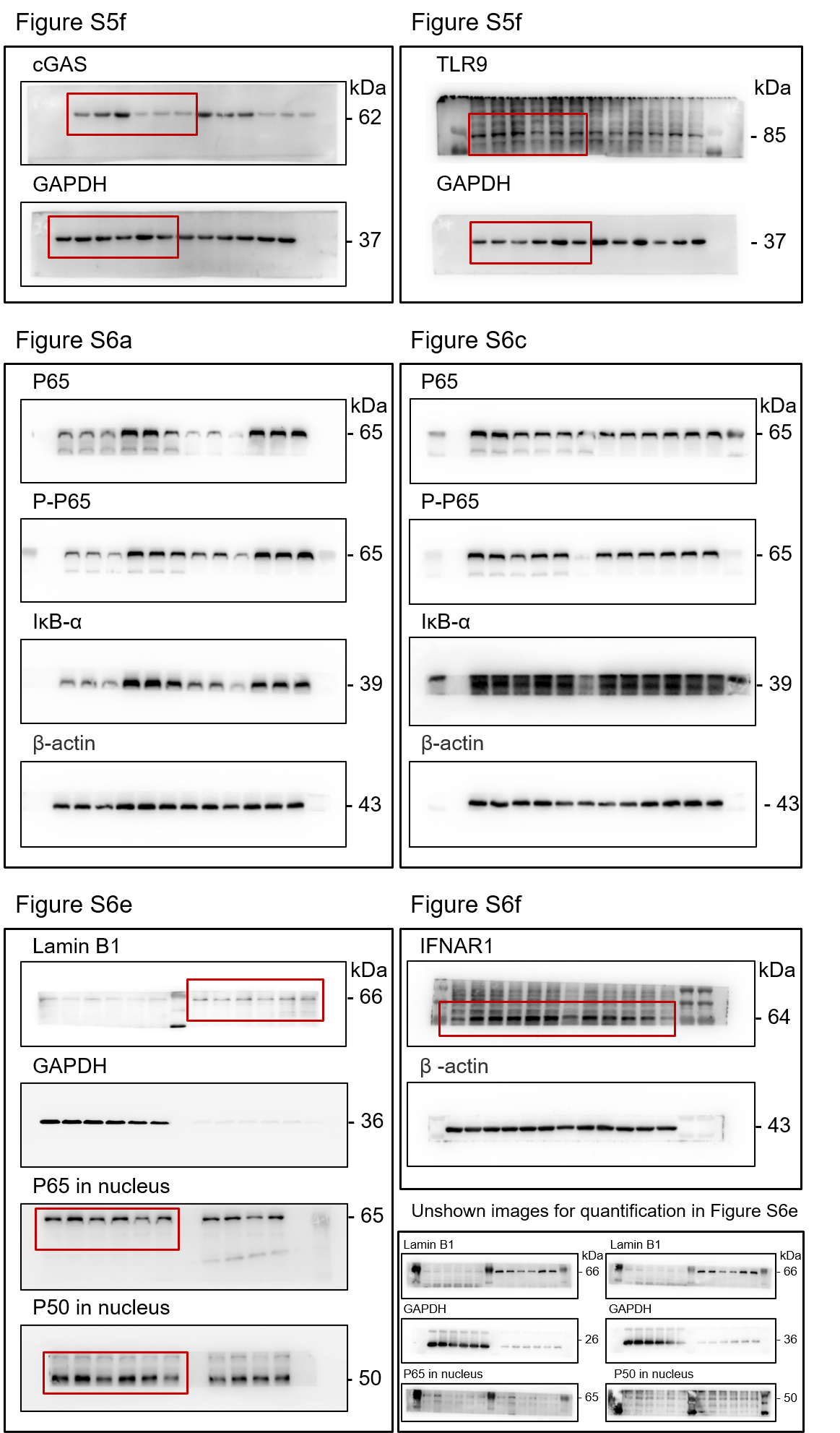


**Figure S18.** Original and uncropped images of Western blots

**Supplementary table S1 to S5**

**Table S1**. Characteristics of healthy participants and patients with COPD receiving bronchoalveolar lavage fluid (BALF) test, and providing peripheral blood for neutrophil extracellular traps (NETs) and dendritic cells (DCs) assay.

**Table S2**. CT parameters and comorbidities of healthy participants and patients with COPD receiving bronchoalveolar lavage fluid (BALF) test, and providing peripheral blood for neutrophil extracellular traps (NETs) and dendritic cells (DCs) assay.


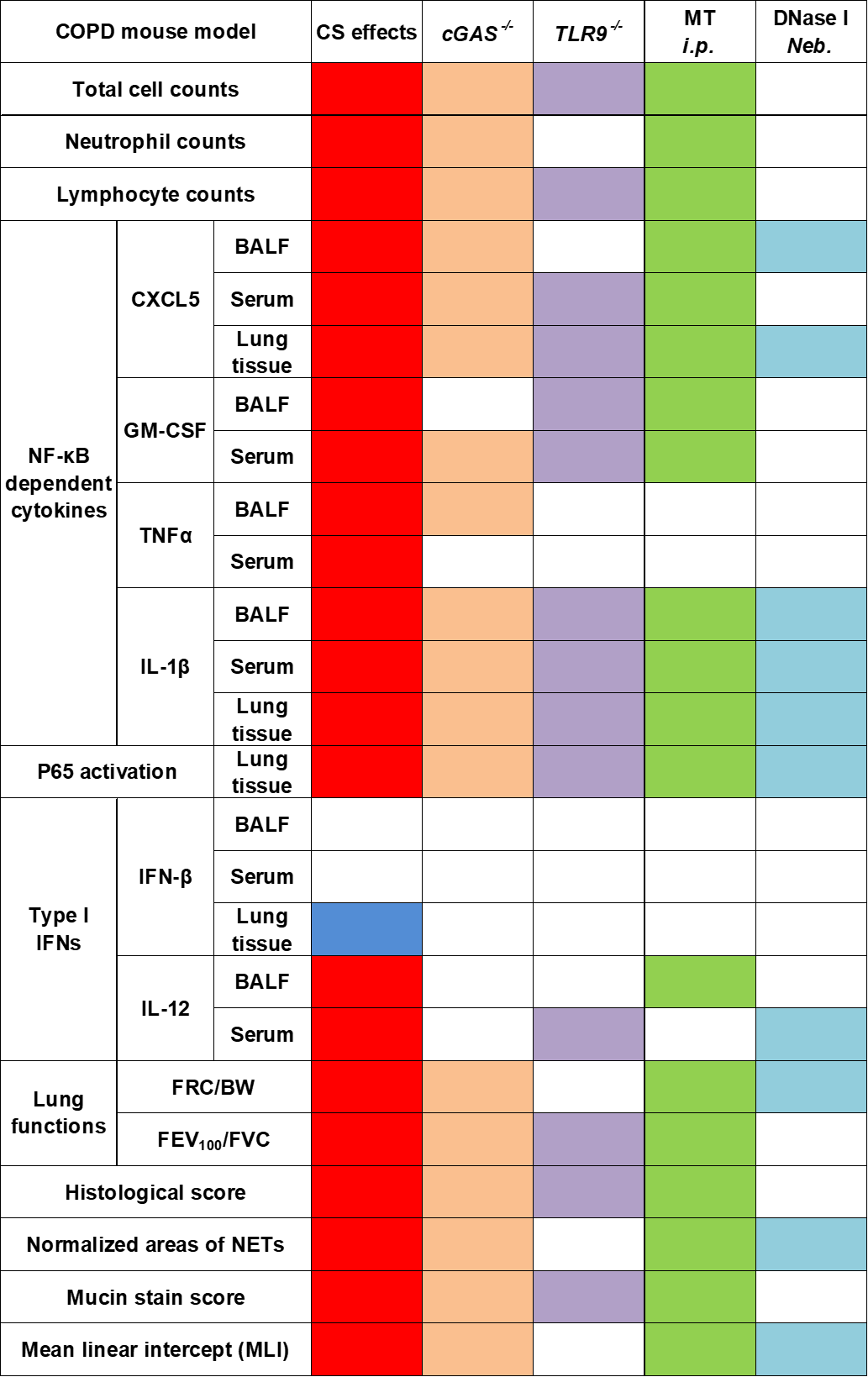


**Table S3**. Summary of improved indicators displayed in cigarette smoke (CS)-treated *cGAS* knock out (*cGAS^-/-^*) mice, *TLR9* knock out (*TLR9^-/-^*) mice, mice treated with mitoTEMPO and deoxyribonuclease I (DNase-I), compared to the CS-treated control mice.

**Table S4**. Characteristics of healthy participants and patients with COPD providing lung tissues for immunofluorescence and immunohistochemical staining (Method 25).

**Table S5**. Primer sequence.

**Supplementary movie S1**


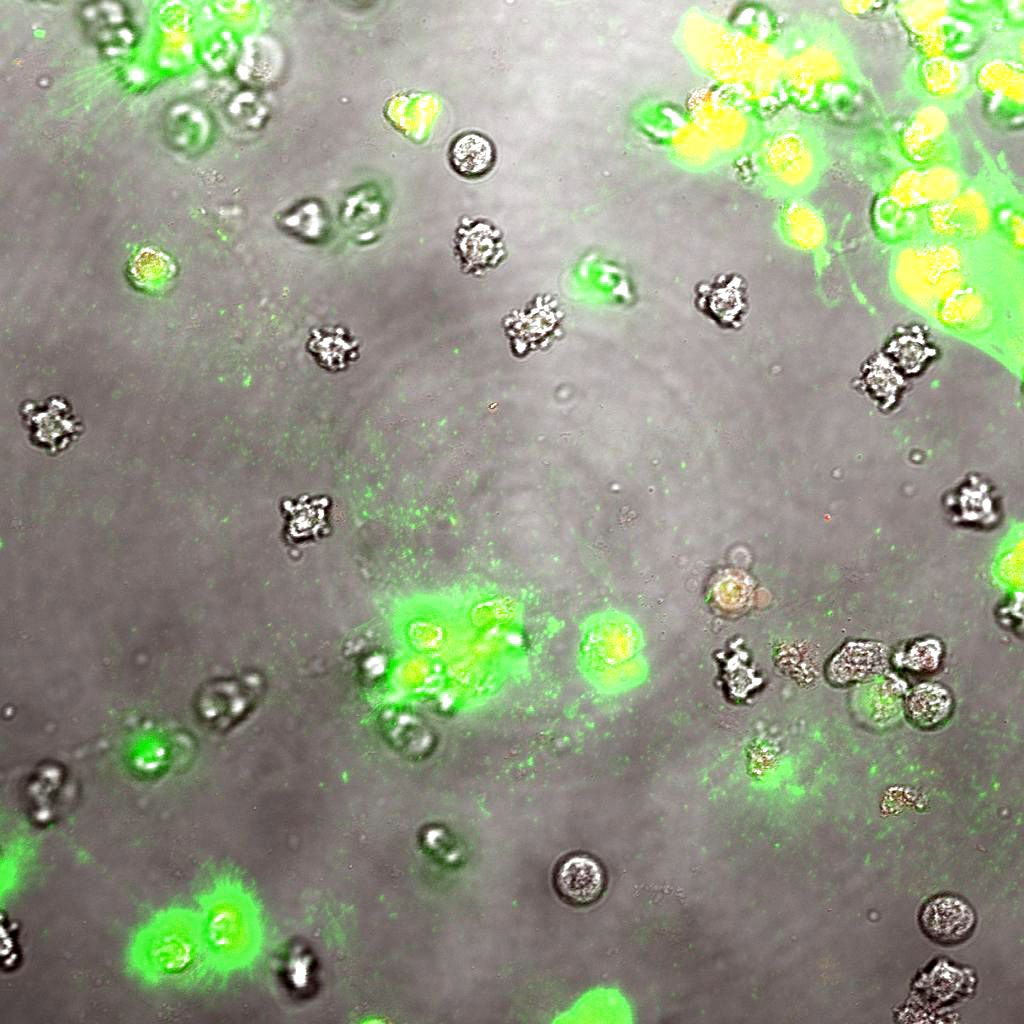


**Movie S1.** A video displays a releasing process of neutrophil extracellular traps induced by 50% cigarette smoke extract, companied by an increased mitochondrial ROS on human circulated neutrophils. The neutrophils were stained with MitoSOX Red (red color) to indicate the level of mitochondrial ROS, the released DNA was stained with SYTOX Green (green color). The video is sampled at 1 frame / 10 s for 30 min, and played back at speed of 200 ×.
